# Supplementary material for: A slow-fast trait continuum at the whole community level in relation to land-use intensification
Source: Nat Commun. 2024 Feb 10;15:1251. doi: 10.1038/s41467-024-45113-5 (PMC10858939; doi:10.1038/s41467-024-45113-5)
Supplement: Supplementary file 1 — Supplementary Information [file 41467_2024_45113_MOESM1_ESM.pdf]

A slow-fast trait continuum at the whole community level in relation to land use intensification

Supplementary Information

Margot Neyret, Gaëtane Le Provost, Andrea Larissa Boesing, Florian D. Schneider, Dennis Baulechner, Joana Bergmann, Franciska de Vries, Anna Maria Fiore-Donno, Stefan Geisen[9], Kezia Goldmann, Anna Merges, Ruslan A. Saifutdinov, Nadja K. Simons, Joseph A. Tobias, Andrey S. Zaitsev, Martin M. Gossner, Kirsten Jung, Ellen Kandeler, Jochen Krauss, Caterina Penone, Michael Schloter, Stefanie Schulz, Michael Staab, Volkmar Wolters, Antonios Apostolakis, Klaus Birkhofer, Steffen Boch, Runa S. Boeddinghaus, Ralph Bolliger, Michael Bonkowski, Francois Buscot, Kenneth Dumack, Markus Fischer, Huei Ying Gan, Johannes Heinze, Norbert Hölzel, Katharina John, Valentin H. Klaus, Till Kleinebecker, Sven Marhan, Jörg Müller, Swen C. Renner, Matthias Rillig, Noëlle V. Schenk, Ingo Schöning, Marion Schrumpf, Sebastian Seibold, Stephanie Socher, Emily F. Solly, Miriam Teuscher, Mark van Kleunen, Tesfaye Wubet, Pete Manning

Supplementary Methods ______ ***2***
Supplementary Tables ______ ***3***
Supplementary Figures ______ ***46***
Supplementary references ______ ***68***

Supplementary Methods

We tested the sensitivity of our results to different parameters.

Role of species turnover versus abundance gradients in driving the results

In addition, we conducted a few additional analyses to assess whether our results were due to species turnover (i.e. species with certain sets of traits disappearing from one end of the land-use intensity gradient to the other) or driven mostly by changes in the abundance few species. For this, we first calculated the turnover and nestedness components of the abundance-based bray-Curtis beta diversity (package betapart, function beta.multi.abund). This was done both for the whole land-use intensity gradient (including all plots) and between the 10 highest-LUI and lowest-LUI plots. This indicated that for most guilds, taxonomic turnover was the main component of species abundance dissimilarities (Table S3).
In addition, we also conducted all the analyses on non-weighted community trait values (i.e. all species present in each plot across the multiple sampling years were given equal abundance of 1). These analyses overall shower weaker, but consistent, results as shown in the main text Tables S11-S14 and Figures S10-S14). This indicates that both abundance changes and species turnover were responsible for the slow-fast community responses observed at the level of the entire community.

Correction for environmental covariates

We also ran the analyses without conducting the environmental corrections on the traits or functions. The community-level results were mostly similar to the main results, except for soil bacterial and fungal communities whose traits strongly differed across regions (Figure S15). The functioning of the system was also strongly affected by region (Figure S19). However, the entire-community slow-fast axis was still identified (Figures S16, S17) and also drove the ecosystem functions slow-fast axis, although less strongly than in the main results (Tables S15-S18, Figures S19, S20). The structure SEM to quantify the relative importance of direct and trophically-mediated LUI effects on the different trophic levels was not as well supported by the data as in the main results, probably due to the effect of the environmental covariates that were not accounted for.

Exclusion of body size data

While there were strong hypothetical reasons to expect body size to be a key trait driving fast-slow variation at the community level, it can respond to a range of drivers and drive life history variation in the absence of other trait responses. As a result, the effect of body mass is often removed before identifying life history trade-offs. We therefore conducted analyses in which we excluded all body size data (resulting in the exclusion of bacterivores and predator protists as other trait data were not available for these groups). We were still able to identify a strong guild-level slow-fast axis for most groups, except collembola. This resulted in somewhat weaker, but still consistent results regarding the synchrony of slow-fast axes across guilds and the effect of the whole community slow-fast axis on ecosystem functioning (Figures S7-S9, Tables S8-S10)

Supplementary Tables

List of supplementary Tables

Table S 1. List of traits, ecosystem functions and environmental covariates used. ... 4
Table S 2. Trait-specific hypothesis testing: expected response of each trait to resource availability (fertilisation) and disturbance (mowing/grazing); overall coverage (number of individual with available trait data / total number of individual for each plot, median (min-max)); number of taxa with data available / total number of taxa; Expected correlation with land-use intensity; test of the hypothesised response. P-values (two-sided t-tests) were corrected for multiple testing (false detection rate, n = 47). ... 5
Table S 3. Abundance-based nestedness (balanced variation) and turnover (abundance gradient) components of Bray-Curtis dissimilarity. The components of dissimilarity were calculated either across all plots, or by first aggregating the 10 highest- and lowest-LUI plots and calculating the dissimilarity between the two groups. Dissimilarities were then calculated using the beta.multi.abund function (package betapart). Colours indicate trophic level (pale to dark colours) and position above (blue) or belowground (brown). ... 15
Table S 4. Comparison of the effect of multiple drivers on the ecosystem functions slow-fast axis, obtained from linear models with the function slow-fast axis as a response and the indicated variables as explanatory variables. P-values (two-sided t-tests) were corrected for multiple testing within these different model (i.e. correction for false discovery rates, R function p.adjust, n = 7). ... 16
Table S 5. Variance partitioning of land use intensity and multivariate trait community weighted mean across sampling plot and years. The partitioning was done using the varpart function (package vegan). Only groups with more than one sampling year are included. For bacteria and fungi, year 2017 was excluded because only one trait was available (% pathogen fungi) which did not allow us to properly partition the variance. Negative shared variance (a common artifact when using varpart) are shown as 0* (or <0.01* if between 0 and -0.01). ... 17
Table S 6. SEM path parameters for the belowground model (fitted with lavaan, boostrapped with 300 iterations). Colours indicate trophic level (pale to dark colours). P-values were extracted using lavaan standardizedSolution function (two-sided t-tests); no further adjustment for multiple testing was made. ... 18
Table S 7. SEM path parameters for the belowground model (fitted with lavaan, boostrapped with 300 iterations). Colours indicate trophic level (pale to dark colours). P-values were extracted using lavaan standardizedSolution function (two-sided t-tests); no further adjustment for multiple testing was made. ... 20
Table S 8. SEM path parameters for the belowground model (fitted with lavaan, boostrapped with 300 iterations). **All body size- and mass-related data were excluded.** Note that the model fit is low, likely due to the influence of environmental variables that were not accounted for. Colours indicate trophic level (pale to dark colours). P-values were extracted using lavaan standardizedSolution function (two-sided t-tests); no further adjustment for multiple testing was made. ... 22
Table S 9. SEM path parameters for the belowground model (fitted with lavaan, boostrapped with 300 iterations). **All body size- and mass-related data were excluded.** Colours indicate trophic level (pale to dark colours). P-values were extracted using lavaan standardizedSolution function (two-sided t-tests); no further adjustment for multiple testing was made. ... 24
Table S 10. Comparison of the effect of multiple drivers on the ecosystem functions slow-fast axis, obtained from linear models with the function slow-fast axis as a response and the indicated variables as explanatory variables. **All body size- and mass-related data were excluded.** P-values (two-sided t-test) were corrected for multiple testing within these different model (i.e. correction for false discovery rates, R function p.adjust, n = 7). ... 25
Table S 11. Trait-specific hypothesis testing: expected response of each trait to resource availability (fertilisation) and disturbance (mowing/grazing) (see Table S2 for detailed hypotheses). Expected correlation with land-use intensity; test of the hypothesised response. P-values (two-sided t-test) were corrected for multiple testing (false detection rate). Empty cells indicate that no specific response to the corresponding driver is expected. Number of taxa with available trait data is considered after extrapolation (see Methods). Total number of taxa includes taxa identified only at higher level (e.g. Genus sp.). *for bacteria, includes genera from which data was extrapolated from other genera in the same Order. Colours indicate trophic level (pale to dark colours) and position above (blue) or belowground (brown). **In contrast to the results shown in Table S2, community-level trait data (CWM) was not weighted by taxa abundance.** ... 26
Table S 12. SEM path parameters for the belowground model (fitted with lavaan, boostrapped with 300 iterations). **Community-level trait data (CWM) was not weighted by taxa abundance.** Colours indicate trophic level (pale to dark colours). P-values were extracted using lavaan standardizedSolution function (two-sided t-tests); no further adjustment for multiple testing was made. ... 31
Table S 13. SEM path parameters for the belowground model (fitted with lavaan, boostrapped with 300 iterations). **Community-level trait data (CWM) was not weighted by taxa abundance**. Note the weaker model fit than in the main results. Colours indicate trophic level (pale to dark colours). P-values were extracted using lavaan standardizedSolution function (two-sided t-tests); no further adjustment for multiple testing was made. ... 33
Table S 14. Comparison of the effect of multiple drivers on the ecosystem functions slow-fast axis, obtained from linear models with the function slow-fast axis as a response and the indicated variables as explanatory variables. **Community-level trait data (CWM) was not weighted by taxa abundance.** P-values (two-sided t-tests) were corrected for multiple testing within these different model (i.e. correction for false discovery rates, R function p.adjust, n = 7). ... 35

Table S 15. Trait-specific hypothesis testing: expected response of each trait to resource availability (fertilisation) and disturbance (mowing/grazing) (see Table S2 for detailed hypotheses). Expected correlation with land-use intensity; test of the hypothesised response. P-values (two-sided t-test) were corrected for multiple testing (false detection rate). Empty cells indicate that no specific response to the corresponding driver is expected. Number of taxa with available trait data is considered after extrapolation (see Methods). Total number of taxa includes taxa identified only at higher level (e.g. Genus sp.). *for bacteria, includes genera from which data was extrapolated from other genera in the same Order. Colours indicate trophic level (pale to dark colours) and position above (blue) or belowground (brown). **Community-level traits (CWM) were not corrected for environmental covariates.** 36
Table S 16. SEM path parameters for the belowground model (fitted with lavaan, boostrapped with 300 iterations). **Community-level traits (CWM) were not corrected for environmental covariates.** Note that the model fit is low, likely due to the influence of environmental variables that were not accounted for. Colours indicate trophic level (pale to dark colours). P-values were extracted using lavaan standardizedSolution function (two-sided t-tests); no further adjustment for multiple testing was made. 41
Table S 17. SEM path parameters for the belowground model (fitted with lavaan, boostrapped with 300 iterations). **Community-level traits (CWM) were not corrected for environmental covariates.** Colours indicate trophic level (pale to dark colours). P-values were extracted using lavaan standardizedSolution function (two-sided t-tests); no further adjustment for multiple testing was made. 43
Table S 18. Comparison of the effect of multiple drivers on the ecosystem functions slow-fast axis, obtained from linear models with the function slow-fast axis as a response and the indicated variables as explanatory variables. **Functions and community-level trait data were not corrected for environmental covariates.** P-values (two-sided t-test) were corrected for multiple testing within these different model (i.e. correction for false discovery rates, R function p.adjust, n = 7). 45

Table S 1. List of traits, ecosystem functions and environmental covariates used.

List of traits per functional guild	
Birds (tertiary consumers)	Generation length, body mass, maximum number of broods per year, incubation time
Bats (tertiary consumers)	Lifespan, body mass, maximum number of offspring
Herb- and litter-dwelling arthropods (secondary consumers)	Dispersal ability, body size
Lepidoptera (primary consumers)	Feeding generalism, length of the flight period, size, voltinism, wintering stage
Other herb- and litter-dwelling arthropods (primary consumers)	Dispersal ability, feeding generalism, voltinism, body size
Vascular plants (primary producers)	Leaf dry matter content, leaf nitrogen content, leaf phosphorus content, root tissue density, seed mass, specific leaf area
Microbial communities (decomposers)	Fungi:bacteria ratio, bacterial genome size, bacterial cell volume, % fungal pathodens, oligotrophic: copiotrophic ratio
Protists (primary consumers)	% of the total protist community
Protists (bacterivores)	Cell size
Protists (secondary consumers)	Cell size
Collembola (omnivores)	Depth preference, voltinism, size, sexual reproduction
Oribatid mites (omnivores)	Days to maturity, feeding specialisation, habitat specialisation, body mass, sexual reproduction
Other belowground arthropods (primary consumers)	Dispersal ability, body mass, feeding generalism
Other belowground arthropods (secondary consumers)	Dispersal ability, body mass
Ecosystem functions	Denitrification Enzyme activity (DEA), activity of urease, abundance of ammonia oxidation gene of archaea, abundance of ammonia oxidation gene of bacteria, abundance of nitrogen fixation gene in soil bacteria, abundance of nitrite oxidation gene of Nitrobacterb acteria, abundance of nitrite oxidizing bacteria, potential nittrification, dung decomposition, root decomposition, litter decomposition, soil respiration, activity of Xylosidase, N-Acetyl beta glucosaminidase, beta-glucosidase, plant above-ground biomass production
Environmental covariates	pH and soil texture, TWI, mean annual temperature

Table S 2. Trait-specific hypothesis testing: expected response of each trait to resource availability (fertilisation) and disturbance (mowing/grazing); overall coverage (number of individual with available trait data / total number of individual for each plot, median (min-max)); number of taxa with data available / total number of taxa; Expected correlation with land-use intensity; test of the hypothesised response. P-values (two-sided t-tests) were corrected for multiple testing (false detection rate, n = 47).

Empty cells indicate that no specific response to the corresponding driver is expected.
Number of taxa with available trait data is considered after extrapolation (see Table 3). Total number of taxa includes taxa identified only at higher level (e.g. Genus sp.). *for bacteria, includes genera from which data was extrapolated from other genera in the same Order. Colours indicate trophic level (pale to dark colours) and position above (blue) or belowground (brown).

Trophic guild	Trait	Expected response to resource availability (fertilisation)	Expected disturbance response to (mowing / grazing)	Plot-level coverage, i.e. proportion (in abundance) with species available trait data)<br><br>median (min-max)	Number of taxa with available trait data /number of taxa identified; number of plots with available data	Expectation: high trait values correspond to fast or slow strategies	Observed response to LUI (slope estimate; 95% confidence interval; adjusted p-value	Response as expected?
Plants (primary producers)	Specific leaf area	High resource availability favours fast-growing species, with rapid resource capture and fast turnover of organs. This translates into high SLA, which allows rapid growth and high resource acquisition but short-lived leaves and hence poor conservation of resources (Grime, 1979; Lavorel and Garnier, 2002; Wright et al., 2004).		99 (61-100)	Taxa: 283/362<br><br>Plots: 150/150	Fast	0.31 (0.17 - 0.46)<br><br>P = 0.00017<br><br>R^2 = 0.10	Yes
	Seed mass		Seed mass reflects a trade-off between colonisation ability and seedling survival (Díaz et al., 2016; Lavorel and Garnier, 2002). Colonisation ability is particularly important at high disturbance to allow for	99 (61-100)	Taxa: 334/362<br><br>Plots: 150/150	Slow	-0.31 (-0.44 – -0.18)<br><br>P = 0.00003<br><br>R^2 = 0.12	Yes

			recolonisation. Further, low seed mass is usually associated with short plant height (Díaz et al., 2016) which is selected by grazing and mowing disturbance.					
	Leaf dry matter content	LDMC is a correlate of specific leaf area (negative correlation), associated with slow growth and good resource conservation. It is a negatively associated with soil fertility (Hodgson et al., 2011).		100 (61-100)	Taxa: 330/362<br><br>Plots: 150/150	Slow	-0.28 (-0.42 – -0.13)<br><br>P = 0.00075<br><br>R^2 = 0.08	Yes
	Leaf nitrogen	High resource availability translates into high leaf nutrient content of fast-growing species (Lavorel and Garnier, 2002).		98 (61-100)	Taxa: 252/362<br><br>Plots: 150/150	Fast	0.46 (0.32 – 0.59)<br><br>P < 10^{-8}<br><br>R^2 = 0.22	Yes
	Leaf phosphorus	High resource availability translates into high leaf nutrient content of fast-growing species (Lavorel and Garnier, 2002).		97 (57-100)	Taxa: 197/362<br><br>Plots: 150/150	Fast	0.51 (0.38 – 0.64)<br><br>P < 10^{-8}<br><br>R^2 = 0.28	Yes
	Root tissue density	Low resource availability favours slow-growing roots with slow turnover that better conserve nutrients (Bergmann et al., 2020; Weigelt et al., 2021).		95 (27-100)	Taxa: 231/362<br>Plots: 150/150	Slow	-0.22 (-0.37 – -0.07)<br><br>P = 0.0066<br><br>R^2 = 0.06	Yes
Lepidoptera (primary consumers, aboveground)	Flight period		An early emergence (i.e. long flight season) allows for growth and reproduction before the start of the disturbance (mowing) (Börschig et al., 2013).	100 (83-100)	Taxa: 90/97<br><br>Plots: 136/150	Fast	0.31 (0.21 – 0.42)<br><br>P < 10^{-8}<br><br>R^2 = 0.20	Yes
	Voltinism		High reproductive rates can compensate for mortality due to disturbance and favour fast	100 (83-100)	Taxa: 88/97	Fast	0.21 (0.06 - 0.37)<br><br>P = 0.014	Yes

			recolonisation (Börschig et al., 2013). In addition disturbance selects for early flowering plants which provide resources earlier in the year and promote multiple generations per year (Börschig et al., 2013).		Plots: 136/150		$R^2 = 0.05$	
	Hibernation stage		A later hibernation stage allows individuals to reproduce before early disturbances and better recolonise a habitat (Börschig et al., 2013).	100 (83-100)	Taxa: 90/97<br><br>Plots: 136/150	Fast	0.27 (0.15 – 0.39)<br><br>$P = 0.00003$<br><br>$R^2 = 0.14$	Yes
	Size (wing size)		Larger wings translate into higher dispersal abilities (which facilitates recolonisation) but larger body sizes makes it more difficult to survive disturbance (Birkhofer et al., 2017; Hanson et al., 2016).	100 (83-100)	Taxa: 90/97<br><br>Plots: 136/150	Fast or slow	-0.07 (-0.22 – 0.08)<br><br>$P = 0.43$<br><br>$R^2 = 0.01$	Inconclusive
	Feeding generalism	Fertilisation reduces plant diversity (Le Provost et al., 2021; Socher et al., 2012), which tends to reduce host availability for specialist species (Chisté et al., 2018).		95 (75-100)	Taxa: 79/97<br><br>Plots: 136/150	Fast	0.30 (0.15 – 0.45)<br><br>$P = 0.00027$<br><br>$R^2 = 0.11$	Yes
Arthropods (primary consumers, aboveground)	Body size	Large resource availability promotes fast pace of life (r strategy), which is usually related to smaller body size (Pianka, 1970).	Smaller body size makes it easier to hide and escape the disturbance (Birkhofer et al., 2015b, 2017; Simons et al., 2016).	100 (0.99-100)	Taxa: 797/803<br><br>Plots: 150/150	Slow	-0.24 (-0.4 – -0.09)<br><br>$P = 0.004$<br><br>$R^2 = 0.06$	Yes
	Feeding generalism	Fertilisation reduces plant diversity (Le Provost et al., 2021; Socher et al., 2012), which tends to reduce host availability for	Generalists are expected to respond less strongly to disturbance in terms of land-	95 (61-99)	Taxa: 621/803	Fast	0.34 (0.21 – 0.47)<br><br>$P < 10^{-8}$	Yes

		specialist species (Chisté et al., 2018).	use intensity (Simons et al., 2016).		Plots: 150/150		$R^2 = 0.15$	
	Dispersal ability		Higher dispersal ability makes it easier to recolonise after disturbance (Birkhofer et al., 2015b, 2017; Simons et al., 2016).	100 (0.99-100)	Taxa: 784/803<br><br>Plots: 150/150	Fast	0.37 (0.25 – 0.49)<br><br>$P < 10^{-8}$<br><br>$R^2 = 0.21$	Yes
	Voltinism	More resources might allow more generations if resources are limiting.	Faster reproduction makes it easier to recover from disturbance	83 (41-98)	Taxa: 202/803<br><br>Plots: 150/150	Fast	0.56 (0.45 – 0.67)<br><br>$P < 10^{-8}$<br><br>$R^2 = 0.40$	Yes
Arthropods (secondary consumers, aboveground)	Body size		Smaller body size makes it easier to hide and escape the disturbance (Birkhofer et al., 2015b, 2017; Simons et al., 2016).	100 (100-100)	Taxa: 240/240<br><br>Plots: 150/150	Slow	0.03 (-0.12 – 0.19)<br><br>$P = 0.76$<br><br>$R^2 = 0$	Inconclusive
	Dispersal ability		Higher dispersal ability makes it easier to recolonise after disturbance (Birkhofer et al., 2015b, 2017; Simons et al., 2016).	100 (0.76-100)	Taxa: 239/240<br><br>Plots: 150/150	Fast	0.33 (0.19 – 0.47)<br><br>$P = 0.00003$<br><br>$R^2 = 0.12$	Yes
Birds (secondary tertiary consumers)	Body mass		Mowing decreases the abundance of large invertebrates on average, but creates resource "flushes" after just after mowing when invertebrate prey are easier to find, resulting in higher foraging efficiency after mowing (Devereux et al., 2006) but overall higher resource availability disparities in time.<br>In addition, mowing also increases mortality and destroys nests of ground-	100 (100-100)	Taxa: 24/24<br><br>Plots: 145/150	Slow	0.23 (0.08 – 0.37)<br><br>$P = 0.005$<br><br>$R^2 = 0.06$	No

			nesting species (Frawley and Best, 1992; MacDonald, 2006).<br>Both mechanisms should promote 'fast' species which are able to recover after disturbance and to rapidly exploit available resources; these are characterised by rapid reproductive traits, typically associated to small size due to energy allocation (Sibly et al., 2012).					
	Incubation time			100 (100-100)	Taxa: 24/24<br><br>Plots: 145/150	Slow	0.12 (-0.03 – 0.27)<br><br>P = 0.20<br><br>R^2 = 0.02	Inconclusive
	Maximum brood per year			100 (100-100)	Taxa: 24/24<br><br>Plots: 145/150	Fast	-0.12 (-0.27 – 0.03)<br><br>P = 0.17<br><br>R^2 = 0.02	Inconclusive
	Generation length			100 (100-100)	Taxa: 24/24<br><br>Plots: 145/150	Slow	0.19 (0.04 – 0.34)<br><br>P = 0.023<br><br>R^2 = 0.04	No
Bats (tertiary consumers, aboveground)	Body mass		Mowing decreases the abundance of large invertebrates on average, but creates resource "flushes" after just after mowing when invertebrate prey are easier to find, resulting in higher foraging efficiency after mowing (Devereux et al., 2006) but overall higher	100 (100-100)	Taxa: 11/11<br><br>Plots: 148/150	Slow	-0.07 (-0.18 – 0.03)<br><br>P = 0.25<br><br>R^2 = 0.01	Inconclusive

			resource availability disparities in time. Both mechanisms should promote 'fast' species which are able to recover after disturbance and to rapidly exploit available resources.					
	Maximum longevity			100 (62-100)	Taxa: 10/11<br><br>Plots: 148/150	Slow	-0.01 (-0.12 – 0.11)<br><br>P = 0.91<br><br>$R^2 = 0$	Inconclusive
	Number of offspring			100 (62-100)	Taxa: 10/11<br><br>Plots: 148/150	Fast	-0.06 (-0.17 – 0.05)<br><br>P = 0.39<br><br>$R^2 = 0.01$	Inconclusive
Protists (plant pathogens i.e. primary consumers)	Relative abundance	High resource availability promotes fast-growing plants which have lower amounts of constitutive defences than slow-growing species; and fast-growing species support higher herbivory rates than slow-growing species (Endara and Coley, 2011). This is likely to promote high abundance of herbivores and pathogens. Empirical evidence supports this; protist pathogens have been shown to respond positively to intensive management (Fiore-Donno et al., 2020).		non applicable	Taxa: 9 genera<br><br>Plots: 150/150	Fast	0.46 (0.32 – 0.59)<br><br>$P < 10^{-8}$<br><br>$R^2 = 0.18$	Yes
Bacteria and fungi	Bacterial cell volume	Alternative hypotheses: a. small cells are more efficient for diffusive uptake; or b. for a given substrate demand, large radius compensates low substrate		42 (27-72)	Taxa: 371/1155*<br><br>Plots: 150/150	fast (a) or slow (b)	-0.30 (-0.41 – -0.19)<br><br>$P < 10^{-8}$<br><br>$R^2 = 0.16$	Yes (hypothesis b)

		concentrations (Westoby et al., 2021).						
	Bacterial oligotroph:copiotroph ratio	By definition, oligotrophic bacteria survive better at low resource availability, while copiotrophic bacteria reproduce faster at high resources availability (Barnett et al., 2021; Fierer et al., 2012; Leff et al., 2015).		50 (29-70)	Taxa: 827/1155*<br><br>Plots: 150/150	slow	-0.14 (-0.27 – -0.01)<br><br>P = 0.022<br><br>$R^2 = 0.03$	Yes
	Bacterial genome size	Bacteria with large genomes are capable to use a larger array of resources in low amounts; thus they are more ecologically successful in environments where resources are scarce but diverse and where there is little penalty for slow growth (Konstantinidis and Tiedje, 2004; Leff et al., 2015).		54 (40-79)	Taxa: 685/1155*<br><br>Plots: 150/150	slow	-0.14 (-0.21 – -0.08)<br><br>P = 0.00015<br><br>$R^2 = 0.11$	Yes
	Fungi:bacteria ratio	Fungi dominate in soils with low resource availability and are associated with “slow” plant communities (Boeddinghaus et al., 2019; de Vries et al., 2012, 2006).		(non applicable)	Taxa: not applicable<br><br>Plots: 150/150	slow	-0.28 (-0.39 – -0.16)<br><br>P = 0.000033<br><br>$R^2 = 0.13$	Yes
	Proportion of fungal pathotrophs among all fungi	High resource availability promote fast-growing plants which have lower amounts of constitutive defences than slow-growing species; and fast-growing species support higher herbivory rates than slow-growing species (Endara and Coley, 2011) which promotes fungal pathogens (Lekberg et al., 2021; Liu et al., 2021).<br><br>Alternatively, pathogens at high intensity might derive from organic amendments such as slurry.		(non applicable)	Taxa: non applicable<br><br>Plots: 150/150	fast	0.37 (0.24 – 0.50)<br><br>$P < 10^{-8}$<br><br>$R^2 = 0.18$	Yes

Protists (bacterivores)	Only one trait, cell size	Low resource availability promotes slow turnover and conservative strategies with larger cell size in protists (Cavalier-Smith, 1980; Lüftenegger et al., 1985). Effect might be indirect via bacterial abundance.		85 (54-98)	Taxa: 26/30 genera<br><br>Plots: 150/150	slow	-0.29 (-0.44 – -0.15)<br><br>P = 0.00028<br><br>R^2 = 0.10	Yes
Protists (secondary consumers)	Only one trait, cell size	Low resource availability promotes slow turnover and conservative strategies with larger cell size in protists (Cavalier-Smith, 1980; Lüftenegger et al., 1985). Effect might be indirect via bacterial and other protists's abundance.		98 (79-100)	Taxa: 31/32 genera<br><br>Plots: 150/150	slow	-0.39 (-0.51 – -0.26)<br><br>$P < 10^{-8}$<br><br>R^2 = 0.20	Yes
Arthropods (primary consumers, belowground)	Body size	Large resource availability promotes fast pace of life (r strategy), which is usually related to smaller body size (Pianka, 1970). Effect might be indirect through consumption of plant roots.	In most arthropods, smaller body size makes it easier to hide and escape the disturbance (Birkhofer et al., 2015b, 2017; Simons et al., 2016).	100 (100-100)	Taxa: 109/109<br><br>Plots: 136/150	slow	-0.24 (-0.41 – -0.11)<br><br>P = 0.002<br><br>R^2 = 0.08	Yes
	Feeding generalism	Fertilisation reduces plant diversity (Le Provost et al., 2021; Socher et al., 2012), which tends to reduce host availability for specialist species (Chisté et al., 2018).		100 (92-100)	Taxa: 107/109<br><br>Plots: 111/150	fast	-0.04 (-0.23 – 0.14)<br><br>P = 0.74<br><br>R^2 = 0.00	Inconclusive
	Dispersal ability		High dispersal ability makes it easier to evade disturbance and to recolonise after disturbance (Birkhofer et al., 2015b, 2017; Simons et al., 2016).	100 (92-100)	Taxa: 107/109<br><br>Plots: 136/150	fast	0.15 (-0.02 – 0.31)<br><br>P = 0.12<br><br>R^2 = 0.02	Inconclusive
Collembola (omnivores, belowground)	Body size	Large resource availability promotes fast pace of life (r strategy), which is usually related to smaller body size (Pianka,	In most arthropods, smaller body size makes it easier to hide and escape the disturbance (Birkhofer et al.,	100 (91-100)	Taxa: 63/64<br><br>Plots: 140/150	Slow	0.02 (-0.13 -- 0.18)<br><br>P = 0.91	Inconclusive

		1970). Effect might be indirect through consumption of plant roots and lower trophic levels.	2015b, 2017; Simons et al., 2016).				$R^2 = 0.00$	
	Depth preference	Species dwelling in deeper horizons are usually considered to have 'faster' traits due to resource availability and soil pore size constraining body size (V. Wolters, and R. Saifutdinov., pers. comm and (Petersen, 1980))		100 (91-100)	Taxa: 63/64<br><br>Plots: 140/150	Fast	-0.01 (-0.16 – 0.14)<br><br>P = 0.91<br><br>$R^2 = 0.00$	Inconclusive
	Voltinism		Fast reproduction (multivoltine species) allows for more reproduction before disturbance and rapid recolonisation after disturbance.	100 (0-100)	Taxa: 55/64<br><br>Plots: 138/150	Fast	-0.09 (-0.24 – 0.06)<br><br>P = 0.34<br><br>$R^2 = 0.01$	Inconclusive
	Reproduction type: sexual		Parthenogenetic species are usually considered as "r" strategists (Petersen, 1980), good at colonising new territories, with especially large population sizes at early succession stages (Chauvat et al., 2007). Indeed, thelytoky (parthenogenetic reproduction of only females) is an advantage for colonization, since there is no need of energy for partner searching.	100 (91-100)	Taxa: 63/64<br><br>Plots: 140/150	Slow	0.00 (-0.15 – 0.16)<br><br>P = 0.96<br><br>$R^2 = 0.00$	Inconclusive
Oribatid mites (omnivores, belowground)	Habitat specificity	More specialised species (in order of specialisation: non-specialised, soil, surface, litter) are usually considered to have 'faster' traits (V. Wolters, and R. Saifutdinov., pers. comm and (Petersen, 1980))		88 (50-100)	Taxa: 51/55<br><br>Plots: 136/150	slow	-0.07 (-0.23 – 0.09)<br><br>P = 0.50<br><br>$R^2 = 0.01$	Inconclusive
	Feeding specialisation	Low resource availability favours high relative fungal availability		88 (50-100)	Taxa: 51/55	slow	0.10 (-0.06 – 0.26)	Inconclusive

		and species richness, providing resources for fungivorous species which are the most specialised (V. Wolters and A Zaytsev, pers. comm).			Plots: 136/150		P = 0.30<br>R^2 = 0.01	
	Reproduction type: sexual		As for Collembola, parthenogenetic species are considered as faster than sexually reproducting species due to higher colonisation abilities and faster reproduction.	88 (50-100)	Taxa: 51/55<br>Plots: 136/150	slow	-0.01 (-0.16 – 0.14)<br>P = 0.91<br>R^2 = 0.00	Inconclusive
	Days to maturity		Shorter maturation time are related to faster reproduction, which makes it easier to recolonise after a disturbance.	88 (50-100)	Taxa: 51/55<br>Plots: 136/150	slow	-0.05 (-0.21 – 0.1)<br>P = 0.60<br>R^2 = 0.00	Inconclusive
	Body mass	Large resource availability promotes fast pace of life (r strategy), which is usually related to smaller body size (Pianka, 1970). Effect might be indirect through consumption of plant roots and lower trophic levels.	In most arthropods, smaller body size makes it easier to hide and escape the disturbance (Birkhofer et al., 2017, 2015a; Simons et al., 2016).	88 (50-100)	Taxa: 51/55<br>Plots: 136/150	slow	-0.18 (-0.32 – -0.04)<br>P = 0.02<br>R^2 = 0.05	Yes
Other arthropods (secondary consumers, belowground)	Body size		In most arthropods, smaller body size makes it easier to hide and escape the disturbance (Birkhofer et al., 2017, 2015a; Simons et al., 2016).	100 (100-100)	Taxa: 205/205<br>Plots: 150/150	slow	-0.02 (-0.16 – 0.1)<br>P = 0.76<br>R^2 = 0.00	Inconclusive
	Dispersal ability		Higher dispersal ability makes it easier to recolonise after disturbance (Birkhofer et al., 2017, 2015a; Simons et al., 2016).	100 (100-100)	Taxa: 205/205<br>Plots: 150/150	fast	0.09 (-0.06 – 0.24)<br>P = 0.32<br>R^2 = 0.01	Inconclusive

Table S 3. Abundance-based nestedness (balanced variation) and turnover (abundance gradient) components of Bray-Curtis dissimilarity. The components of dissimilarity were calculated either across all plots, or by first aggregating the 10 highest- and lowest-LUI plots and calculating the dissimilarity between the two groups. Dissimilarities were then calculated using the beta.multi.abund function (package betapart). Colours indicate trophic level (pale to dark colours) and position above (blue) or belowground (brown).

**Guild**	**Dissimilarity across all plots**			**Dissimilarity between 10 highest- and lowest- LUI plots**		
	Turnover (balanced variation)	Nestedness (abundance gradient)	Overall dissimilarity	Turnover (balanced variation)	Nestedness (abundance gradient)	Overall dissimilarity
Vascular plants (primary producers)	0.967	0.01	0.977	0.679	0.018	0.70
Lepidoptera (primary consumers)	0.957	0.029	0.986	0.457	0.165	0.622
Other arthropods (primary consumers, aboveground)	0.973	0.011	0.984	0.862	0.029	0.892
Arthropods (secondary consumers, aboveground)	0.971	0.014	0.984	0.812	0.001	0.826
Birds (secondary consumers)	0.968	0.020	0.987	0.766	0.060	0.826
Bats	0.912	0.073	0.985	0.518	0.019	0.537
Protists (plant pathogens)	0.884	0.106	0.990	0.894	0.034	0.928
Bacteria and fungi	Non applicable (mix of community- and species-level traits)			Non applicable (mix of community- and species-level traits)		
Protists (bacterivores)	0.960	0.0196	0.980	0.676	0.041	0.717
Protists (secondary consumers)	0.955	0.025	0.980	0.710	0.145	0.855
Arthropods (primary consumers, belowground)	0.971	0.019	0.990	0.864	0.034	0.898
Collembola (omnivores)	0.970	0.017	0.987	0.622	0.169	0.790
Oribatid mites (omnivores)	0.970	0.018	0.987	0.822	0.001	0.824
Arthropods (secondary consumers, belowground)	0.973	0.012	0.984	0.812	0.049	0.861

Table S 4. Comparison of the effect of multiple drivers on the ecosystem functions slow-fast axis, obtained from linear models with the function slow-fast axis as a response and the indicated variables as explanatory variables. P-values (two-sided t-tests) were corrected for multiple testing within these different model (i.e. correction for false discovery rates, R function p.adjust, n = 7).

**Model**	**Slope estimate (+/- standard error)**	**P-value**	**Adj R^2**
Functions slow-fast axis ~ entire community slow-fast axis (based on guild-level PCA)	0.39 (0.04)	$7.0\ 10^{-15}$	34%
Functions slow-fast axis ~ entire community slow-fast axis (based on individual traits)	0.49 (0.05)	$1.2\ 10^{-16}$	38%
Functions slow-fast axis ~ plants slow-fast axis	0.30 (0.05)	$2.6\ 10^{-08}$	19%
Functions slow-fast ~ microbial slow-fast	0.41 (0.06)	$5.2\ 10^{-11}$	25%
Functions slow-fast ~ land-use intensity	0.62 (0.09)	$4.2\ 10^{-11}$	26%
Functions slow-fast ~ taxonomic multidiversity	-0.42 (0.09)	$1.4\ 10^{-05}$	11%
Functions slow-fast ~ Fungal:bacterial ratio	-0.84 (0.07)	$2.0\ 10^{-21}$	47 %

Table S 5. Variance partitioning of land use intensity and multivariate trait community weighted mean across sampling plot and years. The partitioning was done using the varpart function (package vegan). Only groups with more than one sampling year are included. For bacteria and fungi, year 2017 was excluded because only one trait was available (% pathogen fungi) which did not allow us to properly partition the variance. Negative shared variance (a common artifact when using varpart) are shown as 0* (or <0.01* if between 0 and -0.01).

Variable	Variance attributed to the plot only	Variance attributed to the year only	Shared variance between year and plot	Residuals
LUI	0.70	<0.01	0*	0.30
Birds	0.25	0.18	0.02	0.60
Bats	0.43	<0.01	<0.01	0.57
Plants	0.52	0.01	<0.01*	0.47
Arthropods (below-ground, secondary consumers)	0.10	0.03	<0.01	0.87
Arthropods (below-ground, primary consumers)	0.17	0.06	0.02	0.79

Variable	Variance attributed to the plot only	Variance attributed to the year only	Shared variance between year and plot	Residuals
Arthropods (above-ground, secondary consumers)	0.09	0.01	0	0.90
Arthropods (above-ground, primary consumers)	0.23	0.04	<0.01*	0.72
Bacteria and fungi	0.68	0.03	0*	0.24
Protists (secondary consumers)	0.22	<0.01	<0.01	0.78
Protists (bacterivores)	0.15	0.08	0*	0.68
Protists (plant pathogens)	0.38	0.15	0*	0.3

Table S 6. SEM path parameters for the belowground model (fitted with lavaan, boostrapped with 300 iterations). Colours indicate trophic level (pale to dark colours). P-values were extracted using lavaan standardizedSolution function (two-sided t-tests); no further adjustment for multiple testing was made.

**Fit indices**	P-value = 0.98	RMSEA = 0.00	CFI = 1.00	BIC = 2441	
**Regression Slopes**					
**Left-hand side variable**	**Right-hand side variable**	**Estimate**	**Standard error**	**z**	**p**
Plant slow-fast axis	~ land use intensity	0.51	0.07	6.87	.000
Above-ground arthropods (primary consumers) slow-fast axis	~ land use intensity	0.50	0.07	7.61	.000
Above-ground arthropods (primary consumers) slow-fast axis	~ Plant slow-fast axis	0.21	0.06	3.70	.000
Lepidoptera (primary consumers) slow-fast axis	~ land use intensity	0.34	0.09	3.97	.000
Lepidoptera (primary consumers) slow-fast axis	~ Plant slow-fast axis	0.29	0.09	3.08	.002
Above-ground arthropods (secondary consumers) slow-fast axis	~ land use intensity	0.09	0.13	0.67	.503
Above-ground arthropods (secondary consumers) slow-fast axis	~ Plant slow-fast axis	0.00	0.08	0.05	.961
Above-ground arthropods (secondary consumers) slow-fast axis	~ Lepidoptera (primary consumers) slow-fast axis	0.09	0.11	0.84	.399
Above-ground arthropods (secondary consumers) slow-fast axis	~ Above-ground arthropods (primary consumers) slow-fast axis	0.20	0.10	2.04	.041
Birds (tertiary consumers) slow-fast axis	~ land use intensity	-0.25	0.10	-2.46	.014
Birds (tertiary consumers) slow-fast axis	~ Plant slow-fast axis	-0.11	0.10	-1.10	.270
Birds (tertiary consumers) slow-fast axis	~ Lepidoptera (primary consumers) slow-fast axis	-0.07	0.11	-0.61	.542
Birds (tertiary consumers) slow-fast axis	~ Above-ground arthropods (primary consumers) slow-fast axis	0.24	0.14	1.71	.088
Birds (tertiary consumers) slow-fast axis	~ Above-ground arthropods (secondary consumers) slow-fast axis	0.00	0.08	0.03	.974
Bats (tertiary consumers) slow-fast axis	~ land use intensity	0.02	0.12	0.12	.903
Bats (tertiary consumers) slow-fast axis	~ Plant slow-fast axis	0.16	0.10	1.59	.112
Bats (tertiary consumers) slow-fast axis	~ Lepidoptera (primary consumers) slow-fast axis	-0.01	0.09	-0.10	.921
Bats (tertiary consumers) slow-fast axis	~ Above-ground arthropods (primary consumers) slow-fast axis	-0.23	0.11	-2.05	.041
Bats (tertiary consumers) slow-fast axis	~ Above-ground arthropods (secondary consumers) slow-fast axis	-0.05	0.09	-0.50	.618
**Intercepts**					
Plant slow-fast axis		0.00	0.07	0.00	1.000
Above-ground arthropods (primary consumers) slow-fast axis		0.00	0.06	0.00	1.000
Lepidoptera (primary consumers) slow-fast axis		-0.00	0.07	-0.00	.996

Above-ground arthropods (secondary consumers) slow-fast axis		0.00	0.08	0.00	1.000
Birds (tertiary consumers) slow-fast axis		-0.00	0.08	-0.03	.976
Bats (tertiary consumers) slow-fast axis		-0.00	0.09	-0.02	.983
LUI		0 (fixed)			
**Residual Variances**					
Plant slow-fast axis		0.73	0.08	9.05	.000
Above-ground arthropods (primary consumers) slow-fast axis		0.60	0.06	9.54	.000
Lepidoptera (primary consumers) slow-fast axis		0.67	0.09	7.28	.000
Above-ground arthropods (secondary consumers) slow-fast axis		0.89	0.14	6.40	.000
Birds (tertiary consumers) slow-fast axis		0.91	0.22	4.08	.000
Bats (tertiary consumers) slow-fast axis		0.94	0.13	7.45	.000
LUI		1 (fixed)			
**Residual Variances**					
Birds (tertiary consumers) slow-fast axis	Bats (tertiary consumers) slow-fast axis	0.02	0.07	0.27	.789

Table S 7. SEM path parameters for the belowground model (fitted with lavaan, boostrapped with 300 iterations). Colours indicate trophic level (pale to dark colours). P-values were extracted using lavaan standardizedSolution function (two-sided t-tests); no further adjustment for multiple testing was made.

**Fit indices**	P-value = 0.76	RMSEA = 0.00	CFI = 1.00	BIC = 3243	
**Regression Slopes**					
**Left-hand side variable**	**Right-hand side variable**	**Estimate**	**Standard error**	**z**	**p**
Plant slow-fast axis	~ land use intensity	0.51	0.07	6.95	.000
Protists (pathotrophs. i.e.e primary consumers) slow-fast axis	~ land use intensity	0.31	0.08	3.86	.000
Protists (pathotrophs. i.e.e primary consumers) slow-fast axis	~ Plant slow-fast axis	0.34	0.07	4.70	.000
Bacteria and fungi slow-fast axis	~ land use intensity	0.32	0.07	4.84	.000
Bacteria and fungi slow-fast axis	~ Plant slow-fast axis	0.42	0.09	4.82	.000
Protists (bacterivores) slow-fast axis	~ land use intensity	0.40	0.09	4.26	.000
Protists (bacterivores) slow-fast axis	~ Plant slow-fast axis	0.13	0.11	1.20	.231
Protists (bacterivores) slow-fast axis	~ Bacteria and fungi slow-fast axis	-0.28	0.09	-3.16	.002
Protists (secondary consumers) slow-fast axis	~ land use intensity	0.27	0.09	2.97	.003
Protists (secondary consumers) slow-fast axis	~ Plant slow-fast axis	0.03	0.10	0.30	.764
Protists (secondary consumers) slow-fast axis	~ Bacteria and fungi slow-fast axis	0.28	0.11	2.57	.010
Protists (secondary consumers) slow-fast axis	~ Protists (bacterivores) slow-fast axis	0.04	0.07	0.53	.595
Oribatid mites (omnivores) slow-fast axis	~ land use intensity	0.05	0.13	0.42	.677
Oribatid mites (omnivores) slow-fast axis	~ Plant slow-fast axis	0.13	0.09	1.35	.177
Oribatid mites (omnivores) slow-fast axis	~ Bacteria and fungi slow-fast axis	0.07	0.12	0.63	.531
Oribatid mites (omnivores) slow-fast axis	~ Protists (secondary consumers) slow-fast axis	-0.04	0.11	-0.40	.689
Oribatid mites (omnivores) slow-fast axis	~ Protists (bacterivores) slow-fast axis	-0.06	0.08	-0.79	.430
Collembola (omnivores) slow-fast axis	~ land use intensity	-0.05	0.12	-0.44	.658
Collembola (omnivores) slow-fast axis	~ Plant slow-fast axis	0.14	0.10	1.43	.153
Collembola (omnivores) slow-fast axis	~ Bacteria and fungi slow-fast axis	-0.18	0.13	-1.44	.149
Collembola (omnivores) slow-fast axis	~ Protists (bacterivores) slow-fast axis	0.08	0.08	1.07	.285
Collembola (omnivores) slow-fast axis	~ Protists (secondary consumers) slow-fast axis	0.00	0.11	0.01	.992
Arthropods (secondary consumers) slow-fast axis	~ land use intensity	0.16	0.09	1.84	.066
Arthropods (secondary consumers) slow-fast axis	~ Oribatid mites slow-fast axis	0.17	0.09	2.02	.044
Arthropods (secondary consumers) slow-fast axis	~ Collembola slow-fast axis	-0.07	0.08	-0.87	.382
Arthropods (secondary consumers) slow-fast axis	~ Protists (bacterivores) slow-fast axis	-0.13	0.07	-1.76	.079
Arthropods (secondary consumers) slow-fast axis	~ Protists (secondary consumers) slow-fast axis	-0.12	0.08	-1.62	.105
**Intercepts**					
Plant slow-fast axis		0.00	0.07	0.00	1.000
Protists (primary consumers) slow-fast axis		0.00	0.07	0.00	1.000
Bacteria and fungi fast-slow axis		-0.00	0.06	-0.00	1.000
Protists (bacterivores) slow-fast axis		-0.00	0.08	-0.00	1.000
Protists (secondary consumers) slow-fast axis		-0.00	0.06	-0.00	1.000
Oribatid mites (omnivores) slow-fast axis		0.01	0.09	0.09	.925

Collembola (omnivores) slow-fast axis		-0.01	0.09	-0.07	.942
Above-ground arthropods (secondary consumers) slow-fast axis		-0.00	0.08	-0.02	.982
LUI		0 (fixed)			
**Residual Variances**					
Plant slow-fast axis		0.73	0.08	9.53	.000
Protists (primary consumers) slow-fast axis		0.68	0.08	8.97	.000
Bacteria and fungi fast-slow axis		0.58	0.08	7.40	.000
Protists (bacterivores) slow-fast axis		0.85	0.11	7.94	.000
Protists (secondary consumers) slow-fast axis		0.73	0.08	9.36	.000
Oribatid mites (omnivores) slow-fast axis		0.95	0.10	9.45	.000
Collembola (omnivores) slow-fast axis		0.96	0.11	8.62	.000
Above-ground arthropods (secondary consumers) slow-fast axis		0.92	0.13	7.24	.000
LUI		1 (fixed)			
**Residual Variances**					
Protists (primary consumers) slow-fast axis	Bacteria and fungi fast-slow axis	0.16	0.05	3.10	.002
Protists (primary consumers) slow-fast axis	Above-ground arthropods (secondary consumers) slow-fast axis	0 (fixed)			

Table S 8. SEM path parameters for the belowground model (fitted with lavaan, boostrapped with 300 iterations). **All body size- and mass-related data were excluded.** Note that the model fit is low, likely due to the influence of environmental variables that were not accounted for. Colours indicate trophic level (pale to dark colours). P-values were extracted using lavaan standardizedSolution function (two-sided t-tests); no further adjustment for multiple testing was made.

**Fit indices**	P-value = 0.83	RMSEA = 0.	CFI = 1.00	BIC = 2475	
**Regression Slopes**					
**Left-hand side variable**	**Right-hand side variable**	**Estimate**	**Standard error**	**z**	**p**
Plant slow-fast axis	~ land use intensity	0.51	0.07	6.93	.000
Above-ground arthropods (primary consumers) slow-fast axis	~ land use intensity	0.21	0.10	2.15	.032
Above-ground arthropods (primary consumers) slow-fast axis	~ Plant slow-fast axis	0.09	0.09	1.04	.297
Lepidoptera (primary consumers) slow-fast axis	~ land use intensity	0.35	0.08	4.20	.000
Lepidoptera (primary consumers) slow-fast axis	~ Plant slow-fast axis	0.27	0.08	3.22	.001
Above-ground arthropods (secondary consumers) slow-fast axis	~ land use intensity	0.03	0.09	0.40	.686
Above-ground arthropods (secondary consumers) slow-fast axis	~ Plant slow-fast axis	0.29	0.08	3.74	.000
Above-ground arthropods (secondary consumers) slow-fast axis	~ Lepidoptera (primary consumers) slow-fast axis	0.25	0.09	2.65	.008
Above-ground arthropods (secondary consumers) slow-fast axis	~ Above-ground arthropods (primary consumers) slow-fast axis	0.18	0.09	2.13	.033
Birds (tertiary consumers) slow-fast axis	~ land use intensity	-0.10	0.08	-1.25	.213
Birds (tertiary consumers) slow-fast axis	~ Plant slow-fast axis	-0.05	0.09	-0.55	.584
Birds (tertiary consumers) slow-fast axis	~ Lepidoptera (primary consumers) slow-fast axis	-0.03	0.11	-0.29	.774
Birds (tertiary consumers) slow-fast axis	~ Above-ground arthropods (primary consumers) slow-fast axis	-0.08	0.09	-0.91	.364
Birds (tertiary consumers) slow-fast axis	~ Above-ground arthropods (secondary consumers) slow-fast axis	-0.05	0.08	-0.62	.537
Bats (tertiary consumers) slow-fast axis	~ land use intensity	-0.06	0.12	-0.49	.627
Bats (tertiary consumers) slow-fast axis	~ Plant slow-fast axis	0.16	0.11	1.47	.143
Bats (tertiary consumers) slow-fast axis	~ Lepidoptera (primary consumers) slow-fast axis	0.03	0.11	0.28	.778
Bats (tertiary consumers) slow-fast axis	~ Above-ground arthropods (primary consumers) slow-fast axis	-0.18	0.08	-2.15	.032
Bats (tertiary consumers) slow-fast axis	~ Above-ground arthropods (secondary consumers) slow-fast axis	-0.07	0.11	-0.65	.516
**Intercepts**					
Plant slow-fast axis		0.00	0.07	0.00	1.000
Above-ground arthropods (primary consumers) slow-fast axis		0.00	0.08	0.00	1.000
Lepidoptera (primary consumers) slow-fast axis		0.00	0.07	0.01	.994
Above-ground arthropods (secondary consumers) slow-fast axis		-0.00	0.07	-0.00	.998
Birds (tertiary consumers) slow-fast axis		-0.00	0.08	-0.06	.952
Bats (tertiary consumers) slow-fast axis		-0.00	0.07	-0.01	.992

LUI		0 (fixed)			
**Residual Variances**					
Plant slow-fast axis		0.73	0.08	9.60	.000
Above-ground arthropods (primary consumers) slow-fast axis		0.92	0.12	7.50	.000
Lepidoptera (primary consumers) slow-fast axis		0.68	0.09	7.77	.000
Above-ground arthropods (secondary consumers) slow-fast axis		0.69	0.11	6.44	.000
Birds (tertiary consumers) slow-fast axis		0.95	0.25	3.74	.000
Bats (tertiary consumers) slow-fast axis		0.94	0.14	6.62	.000
LUI		1 (fixed)			
**Residual Variances**					
Birds (tertiary consumers) slow-fast axis	Bats (tertiary consumers) slow-fast axis	-0.01	0.07	-0.18	.855

Table S 9. SEM path parameters for the belowground model (fitted with lavaan, boostrapped with 300 iterations). **All body size- and mass-related data were excluded.** Colours indicate trophic level (pale to dark colours). P-values were extracted using lavaan standardizedSolution function (two-sided t-tests); no further adjustment for multiple testing was made.

**Fit indices**	P-value = 0.55	RMSEA = 0.00	CFI = 1.00	BIC = 2009	
**Regression Slopes**					
**Left-hand side variable**	**Right-hand side variable**	**Estimate**	**Standard error**	**z**	**p**
Plant slow-fast axis	~ land use intensity	0.51	0.08	6.34	.000
Protists (pathotrophs. i.e.e primary consumers) slow-fast axis	~ land use intensity	0.31	0.08	3.68	.000
Protists (pathotrophs. i.e.e primary consumers) slow-fast axis	~ Plant slow-fast axis	0.34	0.08	4.04	.000
Bacteria and fungi slow-fast axis	~ land use intensity	0.32	0.07	4.71	.000
Bacteria and fungi slow-fast axis	~ Plant slow-fast axis	0.40	0.09	4.42	.000
Collembola (omnivores) slow-fast axis	~ land use intensity	-0.09	0.11	-0.82	.411
Collembola (omnivores) slow-fast axis	~ Plant slow-fast axis	0.11	0.10	1.10	.270
Collembola (omnivores) slow-fast axis	~ Bacteria and fungi slow-fast axis	-0.04	0.14	-0.26	.798
Arthropods (secondary consumers) slow-fast axis	~ land use intensity	0.09	0.07	1.36	.172
Arthropods (secondary consumers) slow-fast axis	~ Collembola slow-fast axis	-0.10	0.08	-1.23	.218
**Intercepts**					
Plant slow-fast axis		-0.00	0.08	-0.00	1.000
Protists (primary consumers) slow-fast axis		-0.00	0.07	-0.00	1.000
Bacteria and fungi fast-slow axis		-0.00	0.06	-0.00	1.000
Collembola (omnivores) slow-fast axis		-0.00	0.09	-0.05	.958
Above-ground arthropods (secondary consumers) slow-fast axis		-0.00	0.09	-0.01	.996
LUI		0 (fixed)			
**Residual Variances**					
Plant slow-fast axis		0.73	0.08	9.75	.000
Protists (primary consumers) slow-fast axis		0.68	0.07	9.60	.000
Bacteria and fungi fast-slow axis		0.61	0.09	6.46	.000
Collembola (omnivores) slow-fast axis		0.98	0.12	7.97	.000
Above-ground arthropods (secondary consumers) slow-fast axis		0.97	0.12	8.21	.000
LUI		1 (fixed)			
**Residual Coariances**					

Table S 10. Comparison of the effect of multiple drivers on the ecosystem functions slow-fast axis, obtained from linear models with the function slow-fast axis as a response and the indicated variables as explanatory variables. **All body size- and mass-related data were excluded.** P-values (two-sided t-test) were corrected for multiple testing within these different model (i.e. correction for false discovery rates, R function p.adjust, n = 7).

**Model**	**Slope estimate (+/- standard error)**	**P-value**	**Adj R^2**
Functions slow-fast ~ entire community slow-fast	0.41 (0.05)	$9.2\ 10^{-14}$	32 %
Functions slow-fast ~ entire community slow-fast with all traits	0.49 (0.05)	$1.2\ 10^{-16}$	38 %
Functions slow-fast ~ plants slow-fast	0.3 (0.05)	$2.6\ 10^{-8}$	19 %
Functions slow-fast ~ bacteria and fungi slow-fast	0.48 (0.07)	$6.1\ 10^{-11}$	25 %
Functions slow-fast ~ LUI	0.62 (0.09)	$4.2\ 10^{-11}$	26 %
Functions slow-fast ~ taxonomic multidiversity	-0.42 (0.09)	$1.4\ 10^{-05}$	11 %
Functions slow-fast ~ FB ratio	-0.84 (0.07)	$2.0\ 10^{-21}$	47 %

Table S 11. Trait-specific hypothesis testing: expected response of each trait to resource availability (fertilisation) and disturbance (mowing/grazing) (see Table S2 for detailed hypotheses). Expected correlation with land-use intensity; test of the hypothesised response. P-values (two-sided t-test) were corrected for multiple testing (false detection rate). Empty cells indicate that no specific response to the corresponding driver is expected. Number of taxa with available trait data is considered after extrapolation (see Methods). Total number of taxa includes taxa identified only at higher level (e.g. Genus sp.). *for bacteria, includes genera from which data was extrapolated from other genera in the same Order. Colours indicate trophic level (pale to dark colours) and position above (blue) or belowground (brown). **In contrast to the results shown in Table S2, community-level trait data (CWM) was not weighted by taxa abundance.**

Guild	Trait	Expectation: fast or slow trait	Slope estimate (trait CWM ~LUI)	Response as expected?
Vascular plants (primary producers)	Specific Leaf area	Fast	0.36 (0.21 – 0.51)<br>$P < 10^{-6}$<br>$R^2 = 0.14$	Yes
	Seed mass	Slow	-0.21 (-0.35 -- 0.07)<br>$P = 0.01$<br>$R^2 = 0.05$	Yes
	Leaf dry matter content	Slow	-0.31 (-0.45 -- -0.17)<br>$P = 0.00008$<br>$R^2 = 0.12$	Yes
	Leaf nitrogen	Fast	0.42 (0.28 – 0.56)<br>$P < 10^{-6}$<br>$R^2 = 0.19$	Yes
	Leaf phosphorus	Fast	0.51 (0.38 – 0.64)<br>$P < 10^{-6}$<br>$R^2 = 0.28$	Yes
	Root tissue density	Slow	-0.34 (-0.47 -- -0.2)<br>$P < 0.001$<br>$R^2 < 10^{-6}$	Yes
Lepidoptera	Flight period	Fast	0.27 (0.16 – 0.38)<br>$P < 10^{-6}$<br>$R^2 = 0.15$	Yes
	Voltinism	Fast	0.34 (0.19 – 0.49)<br>$P = 0.00008$<br>$R^2 = 0.13$	Yes

	Hibernation stage	Fast	0.27 (0.14 – 0.39)<br>P = 0.0003<br>$R^2 = 0.11$	Yes
	Size (wing size)	Fast or slow	0.15 (0.02 – 0.29)<br>P = 0.05<br>$R^2 = 0.04$	Inconclusive
	Feeding generalism	Fast	0.14 (-0.01 – 0.29)<br>P = 0.11<br>$R^2 = 0$	Inconclusive
Arthropods (primary consumers, aboveground)	Body size	Slow	-0.12 (-0.28 – 0.03)<br>P = 0.20<br>$R^2 = 0.02$	Inconclusive
	Feeding generalism	Fast	0.28 (0.13 – 0.42)<br>P = 0.0009<br>$R^2 = 0.09$	Yes
	Dispersal ability	Fast	0.17 (0.03 – 0.321<br>P = 0.04<br>$R^2 = 0.04$	Yes
	Voltinism	Fast	0.47 (0.35 – 0.59)<br>$P < 10^{-6}$<br>$R^2 = 0.31$	Yes
Arthropods (secondary consumers, aboveground)	Body size	Slow	-0.07 (-0.22 – 0.09)<br>P = 0.52<br>$R^2 = 0.0$	Inconclusive
	Dispersal ability	Fast	0.34 (0.19 – 0.48)<br>P = 0.00005<br>$R^2 = 0.12$	Yes
Birds (secondary consumers)	Body mass	Slow	0.17 (0.02 – 0.32)<br>P = 0.05<br>$R^2 = 0.03$	No

	Incubation time	Slow	0.07 (-0.09 – 0.22)<br>P = 0.51<br>R^2 = 0.01	Inconclusive
	Maximum brood per year	Fast	-0.03 (-0.18 – 0.13)<br>P = 0.80<br>R^2 = 0.0	Inconclusive
	Generation time	Slow	0.13 (-0.02 – 0.28)<br>P = 0.15<br>R^2 = 0.02	Inconclusive
Bats	Body mass	Slow	-0.15 (-0.28 – -0.03)<br>P = 0.04<br>R^2 = 0.04	Yes
	Maximum longevity	Slow	0.02 (-0.13 – 0.17)<br>P = 0.83<br>R^2 =0.0	Inconclusive
	Number of offspring	Fast	-0.08 (-0.22 – 0.05)<br>P = 0.34<br>R^2 = 0.01	Inconclusive
Protists (plant pathogens)	Relative abundance	Fast	Not applicable (based on relative abundances)	
Micro-organisms (bacteria and fungi)	bacterial cell volume	fast (a) or slow (b)	-0.23 (-0.38 -- -0.08)<br>P = 0.008<br>R^2 = 0.06	Yes (hypothesis B)
	Bacterial oligotroph:copiotroph ratio	slow	-0.20 (-0.34 -- -0.08)<br>P = 0.008<br>R^2 = 0.06	Yes
	Bacterial genome size	slow	-0.14 (-0.25 -- -0.03)<br>P = 0.04<br>R^2 = 0.04	Yes
	Fungi:bacteria ratio	slow	Not applicable (based on relative abundances)	

	Proportion of fungal pathotrophs among all fungi	fast	0.37 (0.24 – 0.5)<br>$P < 10^{-6}$<br>$R^2 = 0.18$	Not applicable (based on relative abundances)
Protists (bacterivores)	Only one trait, cell size	slow	-0.19 (-0.33 – -0.05)<br>P = 0.03<br>$R^2 = 0.04$	Yes
Protists (secondary consumers)	Only one trait, cell size	slow	-0.12 (-0.26 -- 0.02)<br>P = 0.16<br>$R^2 = 0.02$	Inconclusive
Arthropods (primary consumers, belowground)	Body size	slow	-0.23 (-0.38 -- -0.07)<br>P = 0.01<br>$R^2 = 0.06$	Yes
	Feeding generalism	fast	-0.06 (-0.24 – 0.12)<br>P = 0.60<br>$R^2 = 0.0$	Inconclusive
	Dispersal ability	fast	0.14 (-0.02 – 0.31)<br>P = 0.15<br>$R^2 = 0.02$	Inconclusive
Collembola	Body size	slow	-0.02 (-0.13 – 0.14)<br>P = 0.83<br>$R^2 = 0.0$	Inconclusive
	Depth preference	fast	-0.00 (-0.16 – 0.15)<br>P = 0.95<br>$R^2 = 0.0$	Inconclusive
	Voltinism	fast	-0.06 (-0.21 – 0.1)<br>P = 0.57<br>$R^2 = 0.0$	Inconclusive
	Reproduction type: sexual	slow	-0.0 (-0.19 – 0.13)<br>P = 0.78<br>$R^2 = 0.0$	Inconclusive

Oribatid mites	Habitat specificity	slow	-0.06 (-0.22 – 0.11)<br>P = 0.55<br>R^2 = 0.0	Inconclusive
	Reproduction type: sexual	slow	-0.04 (-0.2 – 0.11)<br>P = 0.67<br>R^2 = 0.0	Inconclusive
	Days to maturity	slow	-0.10 (-0.25 – 0.05)<br>P = 0.30<br>R^2 = 0.01	Inconclusive
	Body mass	slow	-0.15 (-0.29 – 0.0)<br>P = 0.09<br>R^2 = 0.03	Inconclusive
Arthropods (secondary consumers, belowground)	Body size	slow	0.06 (-0.07 – 0.19)<br>P = 0.51<br>R^2 = 0.01	Inconclusive
	Dispersal ability	fast	0.06 (-0.09 – 0.21)<br>P = 0.56<br>R^2 = 0.0	Inconclusive

Table S 12. SEM path parameters for the belowground model (fitted with lavaan, boostrapped with 300 iterations). **Community-level trait data (CWM) was not weighted by taxa abundance.** Colours indicate trophic level (pale to dark colours). P-values were extracted using lavaan standardizedSolution function (two-sided t-tests); no further adjustment for multiple testing was made.

**Fit indices**	P-value = 0.37	RMSEA = 0.0	CFI = 1.00	BIC = 2443	
**Regression Slopes**					
**Left-hand side variable**	**Right-hand side variable**	**Estimate**	**Standard error**	**z**	**p**
Plant slow-fast axis	~ land use intensity	0.49	0.08	5.91	.000
Above-ground arthropods (primary consumers) slow-fast axis	~ land use intensity	0.38	0.08	4.67	.000
Above-ground arthropods (primary consumers) slow-fast axis	~ Plant slow-fast axis	0.40	0.08	4.96	.000
Lepidoptera (primary consumers) slow-fast axis	~ land use intensity	0.39	0.09	4.37	.000
Lepidoptera (primary consumers) slow-fast axis	~ Plant slow-fast axis	0.11	0.07	1.48	.139
Above-ground arthropods (secondary consumers) slow-fast axis	~ land use intensity	0.27	0.11	2.33	.020
Above-ground arthropods (secondary consumers) slow-fast axis	~ Plant slow-fast axis	-0.00	0.09	-0.05	.962
Above-ground arthropods (secondary consumers) slow-fast axis	~ Lepidoptera (primary consumers) slow-fast axis	0.23	0.11	1.96	.049
Above-ground arthropods (secondary consumers) slow-fast axis	~ Above-ground arthropods (primary consumers) slow-fast axis	-0.07	0.10	-0.71	.480
Birds (tertiary consumers) slow-fast axis	~ land use intensity	-0.07	0.08	-0.88	.378
Birds (tertiary consumers) slow-fast axis	~ Plant slow-fast axis	-0.16	0.08	-2.00	.045
Birds (tertiary consumers) slow-fast axis	~ Lepidoptera (primary consumers) slow-fast axis	-0.10	0.10	-1.03	.305
Birds (tertiary consumers) slow-fast axis	~ Above-ground arthropods (primary consumers) slow-fast axis	0.06	0.08	0.73	.466
Birds (tertiary consumers) slow-fast axis	~ Above-ground arthropods (secondary consumers) slow-fast axis	0.12	0.07	1.59	.113
Bats (tertiary consumers) slow-fast axis	~ land use intensity	-0.13	0.10	-1.22	.223
Bats (tertiary consumers) slow-fast axis	~ Plant slow-fast axis	0.22	0.09	2.48	.013
Bats (tertiary consumers) slow-fast axis	~ Lepidoptera (primary consumers) slow-fast axis	0.12	0.09	1.36	.174
Bats (tertiary consumers) slow-fast axis	~ Above-ground arthropods (primary consumers) slow-fast axis	-0.13	0.10	-1.36	.175
Bats (tertiary consumers) slow-fast axis	~ Above-ground arthropods (secondary consumers) slow-fast axis	-0.19	0.08	-2.26	.024
**Intercepts**					
Plant slow-fast axis		-0.00	0.07	-0.00	1.000
Above-ground arthropods (primary consumers) slow-fast axis		-0.00	0.07	-0.00	1.000
Lepidoptera (primary consumers) slow-fast axis		0.01	0.08	0.11	.913
Above-ground arthropods (secondary consumers) slow-fast axis		-0.00	0.07	-0.03	.980

Birds (tertiary consumers) slow-fast axis		-0.01	0.09	-0.10	.919
Bats (tertiary consumers) slow-fast axis		-0.01	0.09	-0.12	.908
LUI		0 (fixed)			
**Residual Variances**					
Plant slow-fast axis		0.76	0.10	7.28	.000
Above-ground arthropods (primary consumers) slow-fast axis		0.54	0.06	8.61	.000
Lepidoptera (primary consumers) slow-fast axis		0.78	0.14	5.47	.000
Above-ground arthropods (secondary consumers) slow-fast axis		0.85	0.09	9.75	.000
Birds (tertiary consumers) slow-fast axis		0.94	0.16	5.79	.000
Bats (tertiary consumers) slow-fast axis		0.92	0.13	7.23	.000
LUI		1 (fixed)			
**Residual Variances**					
Birds (tertiary consumers) slow-fast axis	Bats (tertiary consumers) slow-fast axis	-0.11	0.08	-1.35	.176

Table S 13. SEM path parameters for the belowground model (fitted with lavaan, boostrapped with 300 iterations). **Community-level trait data (CWM) was not weighted by taxa abundance**. Note the weaker model fit than in the main results. Colours indicate trophic level (pale to dark colours). P-values were extracted using lavaan standardizedSolution function (two-sided t-tests); no further adjustment for multiple testing was made.

**Fit indices**	P-value = 0.29	RMSEA = 0.04	CFI = 0.99	BIC = 412	
**Regression Slopes**					
**Left-hand side variable**	**Right-hand side variable**	**Estimate**	**Standard error**	**z**	**p**
Plant slow-fast axis	~ land use intensity	0.49	0.07	6.75	.000
Protists (pathotrophs. i.e.e primary consumers) slow-fast axis	~ land use intensity	0.07	0.11	0.69	.491
Protists (pathotrophs. i.e.e primary consumers) slow-fast axis	~ Plant slow-fast axis	-0.09	0.12	-0.79	.432
Bacteria and fungi slow-fast axis	~ land use intensity	0.17	0.07	2.50	.012
Bacteria and fungi slow-fast axis	~ Plant slow-fast axis	0.47	0.07	6.48	.000
Protists (bacterivores) slow-fast axis	~ land use intensity	0.18	0.09	2.00	.046
Protists (bacterivores) slow-fast axis	~ Plant slow-fast axis	0.06	0.10	0.60	.550
Protists (bacterivores) slow-fast axis	~ Bacteria and fungi slow-fast axis	0.00	0.10	0.04	.969
Protists (secondary consumers) slow-fast axis	~ land use intensity	0.16	0.11	1.50	.133
Protists (secondary consumers) slow-fast axis	~ Plant slow-fast axis	-0.12	0.09	-1.28	.200
Protists (secondary consumers) slow-fast axis	~ Bacteria and fungi slow-fast axis	0.03	0.10	0.31	.756
Protists (secondary consumers) slow-fast axis	~ Protists (bacterivores) slow-fast axis	0.10	0.08	1.26	.208
Oribatid mites (omnivores) slow-fast axis	~ land use intensity	0.21	0.07	3.00	.003
Oribatid mites (omnivores) slow-fast axis	~ Plant slow-fast axis	0.09	0.09	1.00	.315
Oribatid mites (omnivores) slow-fast axis	~ Bacteria and fungi slow-fast axis	-0.32	0.11	-2.82	.005
Oribatid mites (omnivores) slow-fast axis	~ Protists (secondary consumers) slow-fast axis	-0.03	0.09	-0.37	.710
Oribatid mites (omnivores) slow-fast axis	~ Protists (bacterivores) slow-fast axis	0.02	0.08	0.28	.782
Collembola (omnivores) slow-fast axis	~ land use intensity	-0.06	0.12	-0.52	.606
Collembola (omnivores) slow-fast axis	~ Plant slow-fast axis	0.04	0.10	0.36	.719
Collembola (omnivores) slow-fast axis	~ Bacteria and fungi slow-fast axis	0.03	0.11	0.30	.761
Collembola (omnivores) slow-fast axis	~ Protists (bacterivores) slow-fast axis	0.05	0.09	0.63	.528
Collembola (omnivores) slow-fast axis	~ Protists (secondary consumers) slow-fast axis	0.04	0.07	0.65	.516
Arthropods (secondary consumers) slow-fast axis	~ land use intensity	-0.04	0.08	-0.54	.587
Arthropods (secondary consumers) slow-fast axis	~ Oribatid mites slow-fast axis	0.23	0.08	2.95	.003
Arthropods (secondary consumers) slow-fast axis	~ Collembola slow-fast axis	-0.14	0.08	-1.63	.104
Arthropods (secondary consumers) slow-fast axis	~ Protists (secondary consumers) slow-fast axis	-0.09	0.07	-1.23	.220
Arthropods (secondary consumers) slow-fast axis	~ Protists (bacterivores) slow-fast axis	0.08	0.08	0.96	.339
**Intercepts**					
Plant slow-fast axis		0.00	0.07	0.00	1.000
Protists (primary consumers) slow-fast axis		-0.00	0.09	-0.00	1.000
Bacteria and fungi fast-slow axis		0.00	0.06	0.00	1.000

Protists (bacterivores) slow-fast axis		0.00	0.07	0.00	1.000
Protists (secondary consumers) slow-fast axis		-0.00	0.09	-0.00	1.000
Oribatid mites (omnivores) slow-fast axis		-0.00	0.08	-0.05	.961
Collembola (omnivores) slow-fast axis		0.00	0.09	0.01	.994
Above-ground arthropods (secondary consumers) slow-fast axis		0.00	0.07	0.01	.990
LUI		0 (fixed)			
**Residual Variances**					
Plant slow-fast axis		0.76	0.11	7.12	.000
Protists (primary consumers) slow-fast axis		0.99	0.39	2.55	.011
Bacteria and fungi fast-slow axis		0.66	0.08	7.92	.000
Protists (bacterivores) slow-fast axis		0.95	0.12	7.92	.000
Protists (secondary consumers) slow-fast axis		0.96	0.17	5.51	.000
Oribatid mites (omnivores) slow-fast axis		0.91	0.10	9.44	.000
Collembola (omnivores) slow-fast axis		0.98	0.13	7.54	.000
Above-ground arthropods (secondary consumers) slow-fast axis		0.91	0.10	8.95	.000
LUI		1 (fixed)			
**Residual Coariances**					
Protists (primary consumers) slow-fast axis	Bacteria and fungi fast-slow axis	-0.09	0.05	-1.76	.079
Protists (primary consumers) slow-fast axis		0 (fixed)			

Table S 14. Comparison of the effect of multiple drivers on the ecosystem functions slow-fast axis, obtained from linear models with the function slow-fast axis as a response and the indicated variables as explanatory variables. **Community-level trait data (CWM) was not weighted by taxa abundance.** P-values (two-sided t-tests) were corrected for multiple testing within these different model (i.e. correction for false discovery rates, R function p.adjust, n = 7).

**Model**	**Slope estimate (+/- standard error)**	**P-value**	**Adj R^2**
Functions slow-fast ~ plants slow-fast	0.36 (0.04)	1.9 10^{-14}	32 %
Functions slow-fast ~ microbial slow-fast	0.54 (0.05)	1.3 10^{-21}	46 %
Functions slow-fast ~ whole community slow-fast	0.53 (0.05)	6.7 10^{-23}	48 %
Functions slow-fast ~ whole community slow-fast with all traits	0.61 (0.06)	3.1 10^{-17}	38 %
Functions slow-fast ~ taxonomic multidiversity	-0.42 (0.09)	1.4 10^{-05}	11 %
Functions slow-fast ~ land-use intensity	0.62 (0.09)	2.4 10^{-11}	26 %
Functions slow-fast ~ fungal:bacterial ratio	-0.84 (0.07)	2.8 10^{-22}	47 %

Table S 15. Trait-specific hypothesis testing: expected response of each trait to resource availability (fertilisation) and disturbance (mowing/grazing) (see Table S2 for detailed hypotheses). Expected correlation with land-use intensity; test of the hypothesised response. P-values (two-sided t-test) were corrected for multiple testing (false detection rate). Empty cells indicate that no specific response to the corresponding driver is expected. Number of taxa with available trait data is considered after extrapolation (see Methods). Total number of taxa includes taxa identified only at higher level (e.g. Genus sp.). *for bacteria, includes genera from which data was extrapolated from other genera in the same Order. Colours indicate trophic level (pale to dark colours) and position above (blue) or belowground (brown). **Community-level traits (CWM) were not corrected for environmental covariates.**

**Guild**	**Trait**	**Expectation: fast or slow trait**	**Slope estimate (trait CWM ~LUI)**	**Response as expected?**
Vascular plants (primary producers)	Specific Leaf area	Fast	0.31 (0.16– 0.46)<br>P = 0.0005<br>$R^2 = 0.10$	Yes
	Seed mass	Slow	-0.31 (-0.46 – -0.15)<br>P = 0.0005<br>$R^2 = 0.09$	Yes
	Leaf dry matter content	Slow	-0.29 (-0.45– -0.14)<br>P = 0.0009<br>$R^2 = 0.08$	Yes
	Leaf nitrogen	Fast	0.46 (0.31 – 0.60)<br>$P < 10^{-6}$<br>$R^2 = 0.21$	Yes
	Leaf phosphorus	Fast	0.50 (0.36 – 0.64)<br>$P < 10^{-6}$<br>$R^2 = 0.25$	Yes
	Root tissue density	Slow	-0.28 (-0.44 – -0.13)<br>P = 0.001<br>$R^2 = 0.08$	Yes
Lepidoptera	Flight period	Fast	0.26 (0.1 – 0.42)<br>P = 0.004<br>$R^2 = 0.07$	Yes
	Voltinism	Fast	0.25 (0.1 – 0.41)<br>P = 0.004	Yes

			$R^2 = 0.07$	
	Hibernation stage	Fast	0.25 (0.09 – 0.4)<br>P = 0.006<br>$R^2 = 0.06$	Yes
	Size (wing size)	Fast or slow	-0.12 (-0.28 – 0.04)<br>P = 0.21<br>$R^2 = 0.02$	Inconclusive
	Feeding generalism	Fast	0.31 (0.16 – 0.47)<br>P = 0.0004<br>$R^2 = 0.11$	Yes
Arthropods (primary consumers, aboveground)	Body size	Slow	-0.26 (-0.42 – -0.1)<br>P = 0.003<br>$R^2 = 0.07$	Yes
	Feeding generalism	Fast	0.39 (0.24 – 0.54)<br>$P < 10^{-6}$<br>$R^2 = 0.15$	Yes
	Dispersal ability	Fast	0.28 (0.13 – 0.44)<br>P = 0.001<br>$R^2 = 0.08$	Yes
	Voltinism	Fast	0.54 (0.4 – 0.68)<br>$P < 10^{-6}$<br>$R^2 = 0.29$	Yes
Arthropods (secondary consumers, aboveground)	Body size	Slow	0.08 (-0.08 – 0.25)<br>P = 0.41<br>$R^2 = 0.01$	Inconclusive
	Dispersal ability	Fast	0.32 (0.17 – 0.48)<br>P = 0.0004<br>$R^2 = 0.10$	Yes
Birds (secondary consumers)	Body mass	Slow	0.31 (0.15 – 0.47)<br>P = 0.0005	No

			R^2 = 0.10	
	Incubation time	Slow	0.18 (0.02 – 0.34)<br>P = 0.05<br>R^2 = 0.03	Inconclusive
	Maximum brood per year	Fast	-0.15 (-0.31 – 0.01)<br>P = 0.11<br>R^2 = 0.02	No
	Generation time	Slow	0.27 (0.11 – 0.42)<br>P = 0.004<br>R^2 = 0.07	No
Bats	Body mass	Slow	-0.17 (-0.33 – -0.01)<br>P = 0.064<br>R^2 = 0.03	Inconclusive
	Maximum longevity	Slow	0.06 (-0.1 – 0.23)<br>P = 0.54<br>R^2 = 0.0	Inconclusive
	Number of offspring	Fast	-0.16 (-0.32 – 0.01)<br>P = 0.096<br>R^2 = 0.02	Inconclusive
Protists (plant pathogens)	Relative abundance	Fast	0.31 (0.15 – 0.46)<br>P = 0.0005<br>R^2 = 0.10	Yes
Micro-organisms (bacteria and fungi)	bacterial cell volume	fast (a) or slow (b)	-0.31 (-0.46 – -0.15)<br>P = 0.0005<br>R^2 = 0.09	Yes (hypothesis B)
	Bacterial oligotroph:copiotroph ratio	slow	-0.05 (-0.21 – 0.11)<br>P = 0.64<br>R^2 = 0.0	Inconclusive
	Bacterial genome size	slow	-0.05 (-0.21 – 0.12)<br>P = 0.65	Inconclusive

			$R^2 = 0.0$	
	Fungi:bacteria ratio	slow	-0.24 (-0.4 – -0.08)<br>P = 0.006<br>$R^2 = 0.06$	Yes
	Proportion of fungal pathotrophs among all fungi	fast	0.31 (0.15 – 0.46)<br>P = 0.0005<br>$R^2 = 0.10$	Yes
Protists (bacterivores)	Only one trait, cell size	slow	-0.37 (-0.52 – -0.21)<br>$P < 10^{-6}$<br>$R^2 = 0.13$	Yes
Protists (secondary consumers)	Only one trait, cell size	slow	-0.30 (-0.46 – -0.15)<br>P = 0.0005<br>$R^2 = 0.09$	Yes
Arthropods (primary consumers, belowground)	Body size	slow	-0.25 (-0.41 – -0.09)<br>P = 0.006<br>$R^2 = 0.06$	Yes
	Feeding generalism	fast	-0.09 (-0.28 – 0.1)<br>P = 0.44<br>$R^2 = 0.01$	Inconclusive
	Dispersal ability	fast	0.15 (-0.01 – 0.32)<br>P = 0.11<br>$R^2 = 0.02$	Inconclusive
Collembola	Body size	slow	0.04 (-0.13 – 0.21)<br>P = 0.72<br>$R^2 = 0.0$	Inconclusive
	Depth preference	fast	-0.01 (-0.18 – 0.16)<br>P = 0.94<br>$R^2 = 0.0$	Inconclusive
	Voltinism	fast	-0.09 (-0.26 – 0.08)<br>P = 0.40	Inconclusive

			R^2 = 0.01	
	Reproduction type: sexual	slow	0.01 (-0.16 – 0.18)<br>P = 0.97<br>R^2 = 0.0	Inconclusive
Oribatid mites	Habitat specificity	slow	-0.03 (-0.2 – 0.14)<br>P = 0.78<br>R^2 = 0.0	Inconclusive
	Reproduction type: sexual	slow	0 (-0.17 – 0.17)<br>P = 0.99<br>R^2 = 0.0	Inconclusive
	Days to maturity	slow	-0.06 (-0.23 – 0.11)<br>P = 0.62<br>R^2 = 0.0	Inconclusive
	Body mass	slow	-0.16 (-0.33 – 0)<br>P = 0.10<br>R^2 =0.03	Inconclusive
Arthropods (secondary consumers, belowground)	Body size	slow	-0.01 (-0.17 – 0.15)<br>P = 0.96<br>R^2 = 0.0	Inconclusive
	Dispersal ability	fast	0.11 (-0.05 – 0.27)<br>P = 0.26<br>R^2 = 0.01	Inconclusive

Table S 16. SEM path parameters for the belowground model (fitted with lavaan, boostrapped with 300 iterations). **Community-level traits (CWM) were not corrected for environmental covariates.** Note that the model fit is low, likely due to the influence of environmental variables that were not accounted for. Colours indicate trophic level (pale to dark colours). P-values were extracted using lavaan standardizedSolution function (two-sided t-tests); no further adjustment for multiple testing was made.

**Fit indices**	P-value = 0.02	RMSEA = 0.18	CFI = 0.98	BIC = 2404.58	
**Regression Slopes**					
**Left-hand side variable**	**Right-hand side variable**	**Estimate**	**Standard error**	**z**	**p**
Plant slow-fast axis	~ land use intensity	0.50	0.08	6.34	.000
Above-ground arthropods (primary consumers) slow-fast axis	~ land use intensity	0.36	0.07	5.29	.000
Above-ground arthropods (primary consumers) slow-fast axis	~ Plant slow-fast axis	0.34	0.05	6.23	.000
Lepidoptera (primary consumers) slow-fast axis	~ land use intensity	0.15	0.06	2.37	.018
Lepidoptera (primary consumers) slow-fast axis	~ Plant slow-fast axis	0.32	0.10	3.10	.002
Above-ground arthropods (secondary consumers) slow-fast axis	~ land use intensity	-0.05	0.11	-0.46	.647
Above-ground arthropods (secondary consumers) slow-fast axis	~ Plant slow-fast axis	0.02	0.08	0.30	.764
Above-ground arthropods (secondary consumers) slow-fast axis	~ Lepidoptera (primary consumers) slow-fast axis	0.24	0.08	3.07	.002
Above-ground arthropods (secondary consumers) slow-fast axis	~ Above-ground arthropods (primary consumers) slow-fast axis	0.29	0.10	2.83	.005
Birds (tertiary consumers) slow-fast axis	~ land use intensity	-0.40	0.10	-4.02	.000
Birds (tertiary consumers) slow-fast axis	~ Plant slow-fast axis	-0.09	0.07	-1.20	.228
Birds (tertiary consumers) slow-fast axis	~ Lepidoptera (primary consumers) slow-fast axis	0.08	0.09	0.95	.342
Birds (tertiary consumers) slow-fast axis	~ Above-ground arthropods (primary consumers) slow-fast axis	0.29	0.13	2.19	.028
Birds (tertiary consumers) slow-fast axis	~ Above-ground arthropods (secondary consumers) slow-fast axis	0.06	0.09	0.68	.496
Bats (tertiary consumers) slow-fast axis	~ land use intensity	-0.44	0.09	-4.95	.000
Bats (tertiary consumers) slow-fast axis	~ Plant slow-fast axis	0.16	0.09	1.76	.079
Bats (tertiary consumers) slow-fast axis	~ Lepidoptera (primary consumers) slow-fast axis	0.41	0.07	5.54	.000
Bats (tertiary consumers) slow-fast axis	~ Above-ground arthropods (primary consumers) slow-fast axis	0.12	0.08	1.45	.146
Bats (tertiary consumers) slow-fast axis	~ Above-ground arthropods (secondary consumers) slow-fast axis	0.11	0.07	1.50	.134
**Intercepts**					
Plant slow-fast axis		-0.00	0.07	-0.00	1.000
Above-ground arthropods (primary consumers) slow-fast axis		-0.00	0.06	-0.00	1.000
Lepidoptera (primary consumers) slow-fast axis		0.01	0.08	0.10	.923
Above-ground arthropods (secondary consumers) slow-fast axis		-0.00	0.07	-0.02	.981
Birds (tertiary consumers) slow-fast axis		-0.01	0.08	-0.07	.945
Bats (tertiary consumers) slow-fast axis		-0.01	0.07	-0.19	.850

LUI		0 (fixed)			
**Residual Variances**					
Plant slow-fast axis		0.74	0.09	8.04	.000
Above-ground arthropods (primary consumers) slow-fast axis		0.63	0.06	9.98	.000
Lepidoptera (primary consumers) slow-fast axis		0.80	0.12	6.42	.000
Above-ground arthropods (secondary consumers) slow-fast axis		0.82	0.11	7.23	.000
Birds (tertiary consumers) slow-fast axis		0.85	0.21	4.10	.000
Bats (tertiary consumers) slow-fast axis		0.69	0.08	8.43	.000
LUI		1 (fixed)			
**Residual Variances**					
Birds (tertiary consumers) slow-fast axis	Bats (tertiary consumers) slow-fast axis	0.09	0.06	1.46	.145

Table S 17. SEM path parameters for the belowground model (fitted with lavaan, boostrapped with 300 iterations). **Community-level traits (CWM) were not corrected for environmental covariates.** Colours indicate trophic level (pale to dark colours). P-values were extracted using lavaan standardizedSolution function (two-sided t-tests); no further adjustment for multiple testing was made.

**Fit indices**	P-value = 0.24	RMSEA = 0.04	CFI = 0.99	BIC = 3285	

Regression Slopes

**Left-hand side variable**	**Right-hand side variable**	**Estimate**	**Standard error**	**z**	**p**
Plant slow-fast axis	~ land use intensity	0.50	0.07	6.74	.000
Protists (pathotrophs. i.e.e primary consumers) slow-fast axis	~ land use intensity	0.28	0.09	3.02	.003
Protists (pathotrophs. i.e.e primary consumers) slow-fast axis	~ Plant slow-fast axis	0.36	0.09	4.16	.000
Bacteria and fungi slow-fast axis	~ land use intensity	-0.02	0.07	-0.30	.765
Bacteria and fungi slow-fast axis	~ Plant slow-fast axis	0.56	0.09	6.14	.000
Protists (bacterivores) slow-fast axis	~ land use intensity	0.36	0.09	4.05	.000
Protists (bacterivores) slow-fast axis	~ Plant slow-fast axis	0.14	0.12	1.25	.210
Protists (bacterivores) slow-fast axis	~ Bacteria and fungi slow-fast axis	-0.25	0.08	-3.00	.003
Protists (secondary consumers) slow-fast axis	~ land use intensity	0.33	0.10	3.43	.001
Protists (secondary consumers) slow-fast axis	~ Plant slow-fast axis	-0.06	0.11	-0.55	.582
Protists (secondary consumers) slow-fast axis	~ Bacteria and fungi slow-fast axis	0.20	0.10	2.01	.045
Protists (secondary consumers) slow-fast axis	~ Protists (bacterivores) slow-fast axis	-0.12	0.09	-1.38	.167
Oribatid mites (omnivores) slow-fast axis	~ land use intensity	0.05	0.12	0.44	.658
Oribatid mites (omnivores) slow-fast axis	~ Plant slow-fast axis	0.05	0.10	0.53	.596
Oribatid mites (omnivores) slow-fast axis	~ Bacteria and fungi slow-fast axis	0.14	0.09	1.45	.146
Oribatid mites (omnivores) slow-fast axis	~ Protists (bacterivores) slow-fast axis	-0.05	0.10	-0.48	.632
Oribatid mites (omnivores) slow-fast axis	~ Protists (secondary consumers) slow-fast axis	-0.09	0.08	-1.07	.286
Collembola (omnivores) slow-fast axis	~ land use intensity	-0.10	0.12	-0.84	.399
Collembola (omnivores) slow-fast axis	~ Plant slow-fast axis	0.14	0.10	1.45	.147
Collembola (omnivores) slow-fast axis	~ Bacteria and fungi slow-fast axis	-0.18	0.10	-1.83	.067
Collembola (omnivores) slow-fast axis	~ Protists (bacterivores) slow-fast axis	0.09	0.09	1.01	.314
Collembola (omnivores) slow-fast axis	~ Protists (secondary consumers) slow-fast axis	-0.04	0.10	-0.44	.663
Arthropods (secondary consumers) slow-fast axis	~ land use intensity	0.08	0.08	1.02	.310
Arthropods (secondary consumers) slow-fast axis	~ Oribatid mites slow-fast axis	0.36	0.07	5.30	.000
Arthropods (secondary consumers) slow-fast axis	~ Collembola slow-fast axis	-0.21	0.07	-2.81	.005
Arthropods (secondary consumers) slow-fast axis	~ Protists (secondary consumers) slow-fast axis	-0.04	0.09	-0.46	.647
Arthropods (secondary consumers) slow-fast axis	~ Protists (bacterivores) slow-fast axis	-0.08	0.08	-0.96	.335

**Intercepts**					
Plant slow-fast axis		0.00	0.08	0.00	1.000
Protists (primary consumers) slow-fast axis		-0.00	0.06	-0.00	1.000
Bacteria and fungi fast-slow axis		0.00	0.07	0.00	1.000
Protists (bacterivores) slow-fast axis		-0.00	0.07	-0.00	1.000
Protists (secondary consumers) slow-fast axis		-0.00	0.08	-0.00	1.000
Oribatid mites (omnivores) slow-fast axis		-0.00	0.08	-0.00	.997
Collembola (omnivores) slow-fast axis		-0.01	0.09	-0.10	.921
Above-ground arthropods (secondary consumers) slow-fast axis		-0.00	0.07	-0.02	.982
LUI		0 (fixed)			
**Residual Variances**					
Plant slow-fast axis		0.74	0.09	8.38	.000
Protists (primary consumers) slow-fast axis		0.68	0.07	9.80	.000
Bacteria and fungi fast-slow axis		0.70	0.07	10.48	.000
Protists (bacterivores) slow-fast axis		0.82	0.13	6.48	.000
Protists (secondary consumers) slow-fast axis		0.85	0.10	8.52	.000
Oribatid mites (omnivores) slow-fast axis		0.95	0.09	10.54	.000
Collembola (omnivores) slow-fast axis		0.95	0.11	8.98	.000
Above-ground arthropods (secondary consumers) slow-fast axis		0.80	0.12	6.60	.000
LUI		1 (fixed)			
**Residual Coariances**					
Protists (primary consumers) slow-fast axis	Bacteria and fungi fast-slow axis	0.19	0.05	3.78	.000
Protists (primary consumers) slow-fast axis		0 (fixed)			

Table S 18. Comparison of the effect of multiple drivers on the ecosystem functions slow-fast axis, obtained from linear models with the function slow-fast axis as a response and the indicated variables as explanatory variables. **Functions and community-level trait data were not corrected for environmental covariates.** P-values (two-sided t-test) were corrected for multiple testing within these different model (i.e. correction for false discovery rates, R function p.adjust, n = 7).

**Model**	**Slope estimate (+/- standard error)**	**P-value**	**Adj R^2**
Functions slow-fast ~ entire community slow-fast	0.29 (0.05)	$8.4\ 10^{-8}$	18 %
Functions slow-fast ~ entire community slow-fast with all traits	0.42 (0.06)	$1.4\ 10^{-10}$	26 %
Functions slow-fast ~ plants slow-fast	0.17 (0.06)	$3.1\ 10^{-3}$	5 %
Functions slow-fast ~ bacteria and fungi slow-fast	0.25 (0.06)	$2.3\ 10^{-4}$	9 %
Functions slow-fast ~ LUI	0.35 (0.1)	$4.3\ 10^{-4}$	8 %
Functions slow-fast ~ taxonomic multidiversity	-0.37 (0.1)	$3.4\ 10^{-4}$	8 %
Functions slow-fast ~ FB ratio	-0.59 (0.09)	$2.0\ 10^{-9}$	22 %

Supplementary Figures

List of supplementary Figures

Figure S 1. Variance partitioning of each trait CWM (before environmental correction) between land use intensity and all the environmental covariate considered. Proportion of variance explained by each variable is calculated as the proportion of sum of squares associated to each variable in a linear model, with the trait CWM as response variable and all other as explanatory variables. 48
Figure S 2. Correlation between guild-level PCA axes or single traits and land use intensity. Three axes were retained when more than two traits were available; otherwise only two were retained. For protists, only one trait was available, and the correlation between the CWM of this trait and LUI is shown. The fast-slow axis is always the first PC axis, except for arthropod (secondary consumers) above-ground, for which it was PC axis 2. The axes were transformed were needed (inversed sign) so that higher axis values indicate “faster” strategies. P-values were corrected for false detection rates (***: P < 0.001, **: P < 0.01, *: P< 0.05, n.s.: P > 0.05)........ 49
Figure S 3. Theoretical SEMs for above-and below ground trophic levels........ 50
Figure S 4. Direct and indirect links between land- use intensity, the functional traits slow-fast axis and the ecosystem function slow-fast axis. **Functional trait slow-fast axis was measured as the first axis of a PCA with all selected traits as variables (Figure 3b, PC1), rather than individual guild slow-fast axes (Figure 3a, PC1).**........ 51
Figure S 5. Pearson correlations between individual traits CWM (and overall community slow-fast trait axis) and functions (and overall slow-fast functioning axis). Both functions and traits were corrected for the environment beforehand. Icons were acquired and adapted from Phylopic.org (artists: M. Dahirel, B. Lang, M. Crook, J. A. Venter, H. H. T. Prins, D. A. Balfour, R. Slotow, T. M. Keesey, A. A. Farke, Y. Wong, G. Monger). 52
Figure S 6. Pearson correlations between individual guild slow-fast axis (and overall community slow-fast trait axis) and functions bundles (and overall slow-fast functioning axis). Both functions and traits were corrected for the environment beforehand. 53
Figure S 7. Identification of guild-level slow-fast axes. **Analyses excluded all size- and body mass-related traits.** Icons were acquired and adapted from Phylopic.org (artists: M. Dahirel, B. Lang, M. Crook, J. A. Venter, H. H. T. Prins, D. A. Balfour, R. Slotow, T. M. Keesey, A. A. Farke, Y. Wong, G. Monger). 54
Figure S 8. Synchronised slow-fast trait response of individual guilds is strongly related to land-use intensity. **Analyses excluded all size- and body mass-related traits**. The variables included in the PCA are the slow-fast axes of each guild. Land-use intensity, added as a supplementary variable, was strongly associated with axis 1. Belowground guilds are shown in brown, aboveground guilds in blue. Icons were acquired and adapted from Phylopic.org (artists: M. Dahirel, B. Lang, M. Crook, J. A. Venter, H. H. T. Prins, D. A. Balfour, R. Slotow, T. M. Keesey, A. A. Farke, Y. Wong, G. Monger). 55
Figure S 9. Direct and trophically mediated effects of land-use intensity on the slow-fast axis of different trophic levels**. Analyses excludes size- and body mass- related traits.** a. Full SEMs including all guilds. Two independent models were fitted for below- and aboveground guilds; plants being included in both. b. Average direct, indirect and total LUI effects on each trophic level (averaged from the full SEM). c. Decreasing direct, indirect and total LUI effects with trophic level. Each dot represents the estimated effect (+/- standard error) of an individual guild in the full SEM. Icons were acquired and adapted from Phylopic.org (artists: M. Dahirel, B. Lang, M. Crook, J. A. Venter, H. H. T. Prins, D. A. Balfour, R. Slotow, T. M. Keesey, A. A. Farke, Y. Wong, G. Monger)........ 56
Figure S 10. Identification of guild-level slow-fast axes. In contrast to the results shown as Figure 2**, community-level trait data (CWM) was not weighted by taxa abundance.** Icons were acquired and adapted from Phylopic.org (artists: M. Dahirel, B. Lang, M. Crook, J. A. Venter, H. H. T. Prins, D. A. Balfour, R. Slotow, T. M. Keesey, A. A. Farke, Y. Wong, G. Monger)........ 57
Figure S 11. Correlation between guild-level PCA axes or single traits and land use intensity. Three axes were retained when more than two traits were available; otherwise only two were retained. For protists, only one trait was available, and the correlation between the CWM of this trait and LUI is shown. The fast-slow axis is always the first PC axis, except for arthropod (primary and secondary consumers) above-ground, for which it was PC axis 2. The axes were transformed were needed (inversed sign) so that higher axis values indicate “faster” strategies. P-values were corrected for false detection rates (***: P < 0.001, **: P < 0.01, *: P< 0.05, n.s.: P > 0.05). **Community-level trait data (CWM) was not weighted by taxa abundance.**........ 58
Figure S 12. Synchronised slow-fast trait response of individual guilds is strongly related to land-use intensity. **Community-level trait data (CWM) was not weighted by taxa abundance**. The variables included in the PCA are the slow-fast axes of each guild. Land-use intensity, added as a supplementary variable, was strongly associated with axis 1. Belowground guilds are shown in brown,

aboveground guilds in blue. Icons were acquired and adapted from Phylopic.org (artists: M. Dahirel, B. Lang, M. Crook, J. A. Venter, H. H. T. Prins, D. A. Balfour, R. Slotow, T. M. Keesey, A. A. Farke, Y. Wong, G. Monger)........ 59

Figure S 13. Direct and trophically mediated effects of land-use intensity on the slow-fast axis of different trophic levels. **Community-level trait data (CWM) was not weighted by taxa abundance**. a. Full SEMs including all guilds. Two independent models were fitted for below- and aboveground guilds; plants being included in both. b. Average direct, indirect and total LUI effects on each trophic level (averaged from the full SEM). c. Decreasing direct, indirect and total LUI effects with trophic level. Each dot represents the estimated effect (+/- standard error) of an individual guild in the full SEM. Icons were acquired and adapted from Phylopic.org (artists: M. Dahirel, B. Lang, M. Crook, J. A. Venter, H. H. T. Prins, D. A. Balfour, R. Slotow, T. M. Keesey, A. A. Farke, Y. Wong, G. Monger). 60

Figure S 14. Direct and indirect links between land- use intensity, functional traits slow-fast axis and ecosystem function slow-fast axis. **In contrast to Figure 6, community-level trait data (CWM) was not weighted by taxa abundance.**........ 61

Figure S 15. Identification of guild-level slow-fast axes. **Community-level traits (CWM) were not corrected for environmental covariates**. Different colors show the different regions of the Exploratories. Icons were acquired and adapted from Phylopic.org (artists: M. Dahirel, B. Lang, M. Crook, J. A. Venter, H. H. T. Prins, D. A. Balfour, R. Slotow, T. M. Keesey, A. A. Farke, Y. Wong, G. Monger). 62

Figure S 16. Correlation between guild-level PCA axes or single traits and land use intensity. Three axes were retained when more than two traits were available; otherwise only two were retained. For protists, only one trait was available, and the correlation between the CWM of this trait and LUI is shown. The fast-slow axis is always the first PC axis, except for arthropod (secondary consumers) above-ground, for which it was PC axis 2. The axes were transformed were needed (inversed sign) so that higher axis values indicate "faster" strategies. P-values were corrected for false detection rates (***: P < 0.001, **: P < 0.01, *: P< 0.05, n.s.: P > 0.05). **Community-level traits (CWM) were not corrected for environmental covariates**. 63

Figure S 17. Synchronised slow-fast trait response of individual guilds is strongly related to land-use intensity. **Community-level traits (CWM) were not corrected for environmental covariates**. The variables included in the PCA are the slow-fast axes of each guild. Land-use intensity, added as a supplementary variable, was strongly associated with axis 1. Belowground guilds are shown in brown, aboveground guilds in blue. Icons were acquired and adapted from Phylopic.org (artists: M. Dahirel, B. Lang, M. Crook, J. A. Venter, H. H. T. Prins, D. A. Balfour, R. Slotow, T. M. Keesey, A. A. Farke, Y. Wong, G. Monger)........ 64

Figure S 18. Direct and trophically mediated effects of land-use intensity on the slow-fast axis of different trophic levels**. Community-level traits (CWM) were not corrected for environmental covariates.** a. Full SEMs including all guilds. Two independent models were fitted for below- and aboveground guilds; plants being included in both. b. Average direct, indirect and total LUI effects on each trophic level (averaged from the full SEM). c. Decreasing direct, indirect and total LUI effects with trophic level. Each dot represents the estimated effect (+/- standard error) of an individual guild in the full SEM. Icons were acquired and adapted from Phylopic.org (artists: M. Dahirel, B. Lang, M. Crook, J. A. Venter, H. H. T. Prins, D. A. Balfour, R. Slotow, T. M. Keesey, A. A. Farke, Y. Wong, G. Monger). 65

Figure S 19. Identification of ecosystem functions slow-fast axis. **Functions were not corrected for environmental covariates.** For all functions, high values are expected to be related to "fast" ecosystem functioning. Opposite response of dung decomposition compared to others is explained by the negative effect of land-use intensity (especially mowing) on the abundance and activity of dung beetles 66

Figure S 20. Direct and indirect links between land- use intensity, functional traits slow-fast axis and ecosystem function slow-fast axis. **In contrast to Figure 6, functions and community-level trait data were not corrected for environmental covariates.** 67

Figure S 1. Variance partitioning of each trait CWM (before environmental correction) between land use intensity and all the environmental covariate considered. Proportion of variance explained by each variable is calculated as the proportion of sum of squares associated to each variable in a linear model, with the trait CWM as response variable and all other as explanatory variables.

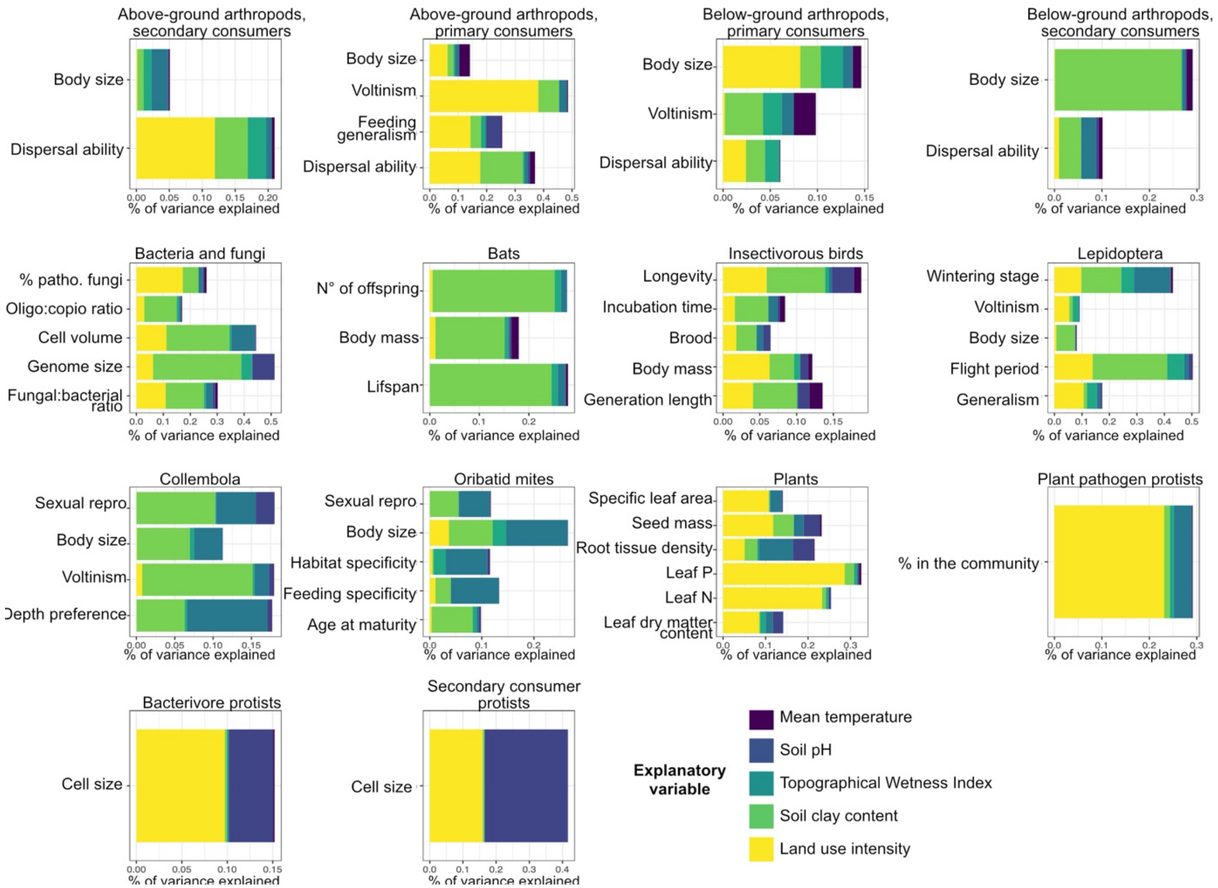

Figure S 2. Correlation between guild-level PCA axes or single traits and land use intensity. Three axes were retained when more than two traits were available; otherwise only two were retained. For protists, only one trait was available, and the correlation between the CWM of this trait and LUI is shown. The fast-slow axis is always the first PC axis, except for arthropod (secondary consumers) above-ground, for which it was PC axis 2. The axes were transformed were needed (inversed sign) so that higher axis values indicate "faster" strategies. P-values were corrected for false detection rates (***: P < 0.001, **: P < 0.01, *: P< 0.05, n.s.: P > 0.05)

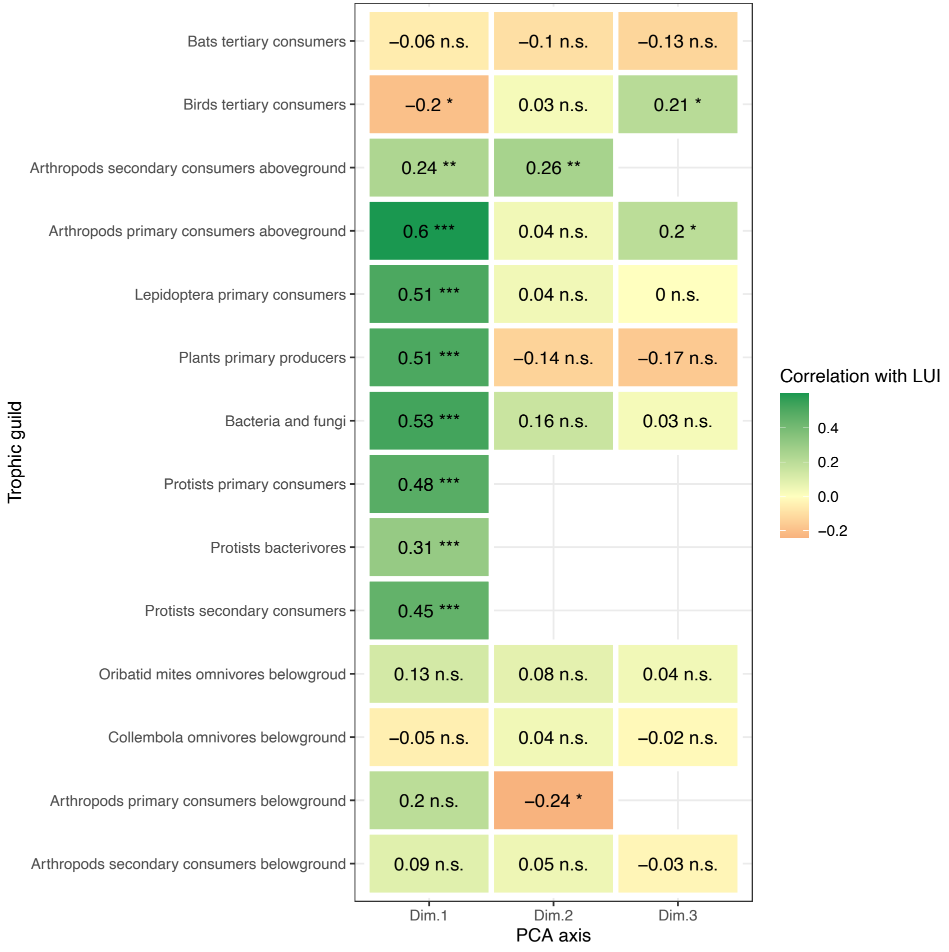

Figure S 3. Theoretical SEMs for above-and below ground trophic levels

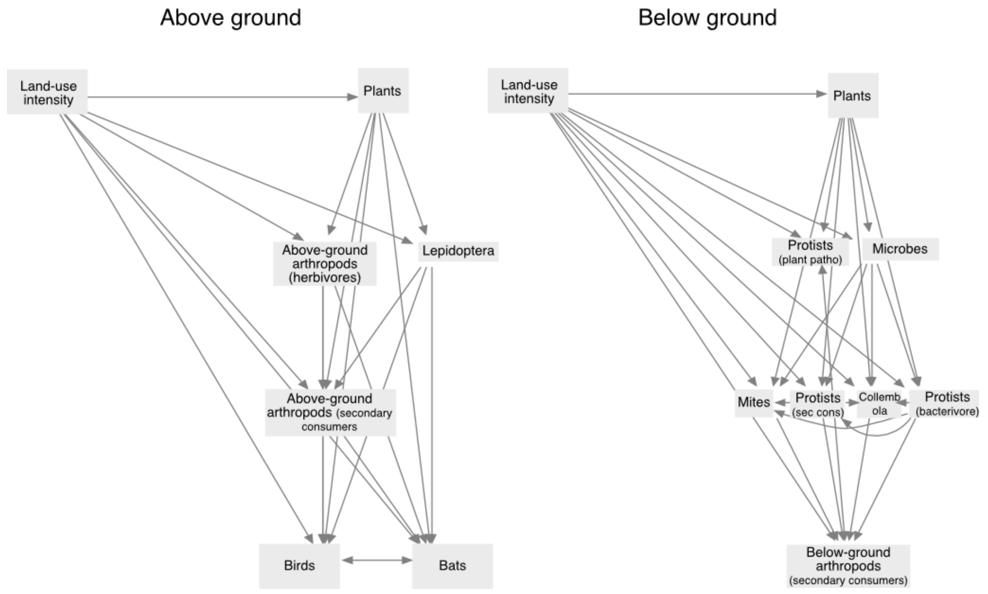

Figure S 4. Direct and indirect links between land- use intensity, the functional traits slow-fast axis and the ecosystem function slow-fast axis. **Functional trait slow-fast axis was measured as the first axis of a PCA with all selected traits as variables (Figure 3b, PC1), rather than individual guild slow-fast axes (Figure 3a, PC1).**

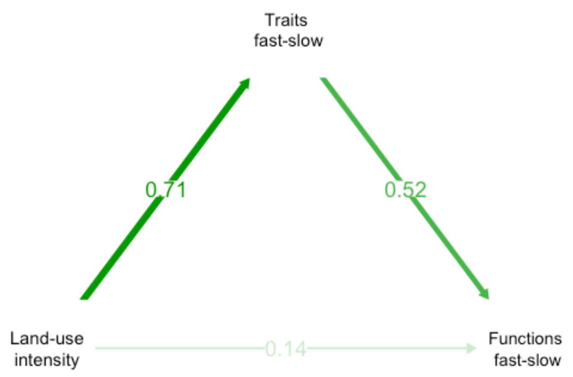

Figure S 5. Pearson correlations between individual traits CWM (and overall community slow-fast trait axis) and functions (and overall slow-fast functioning axis). Both functions and traits were corrected for the environment beforehand. Icons were acquired and adapted from Phylopic.org (artists: M. Dahirel, B. Lang, M. Crook, J. A. Venter, H. H. T. Prins, D. A. Balfour, R. Slotow, T. M. Keesey, A. A. Farke, Y. Wong, G. Monger).

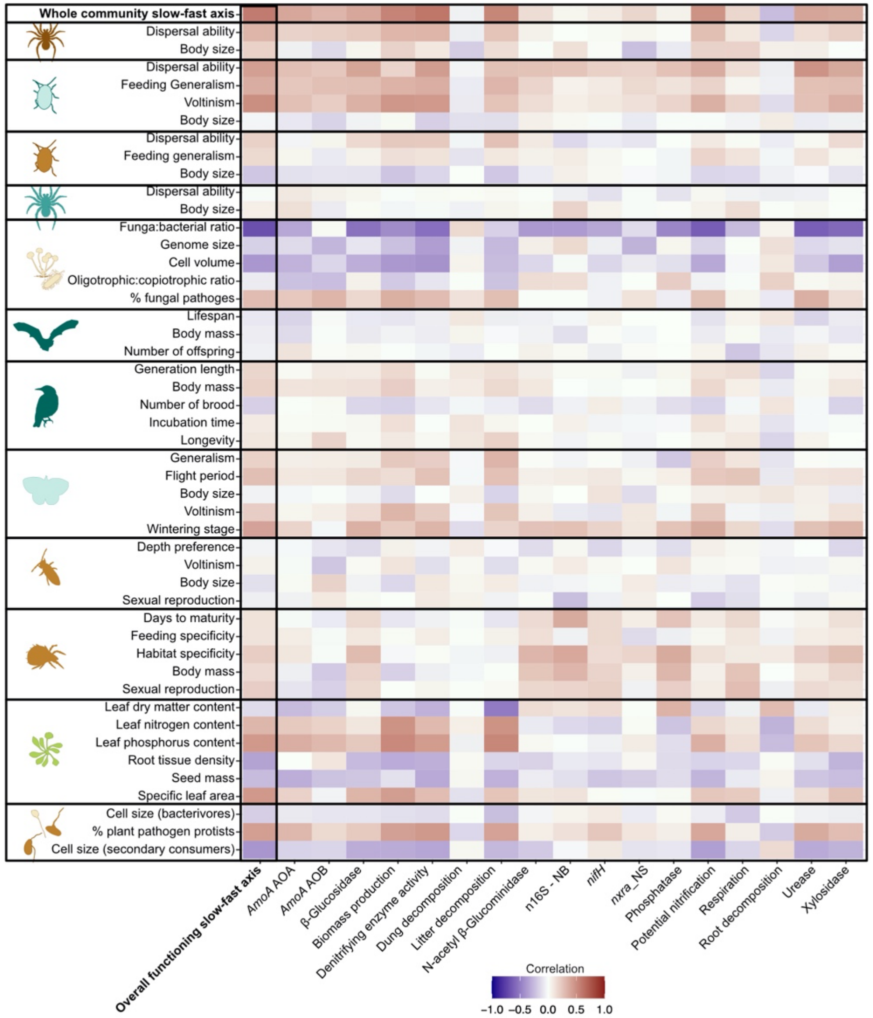

Figure S 6. Pearson correlations between individual guild slow-fast axis (and overall community slow-fast trait axis) and functions bundles (and overall slow-fast functioning axis). Both functions and traits were corrected for the environment beforehand.

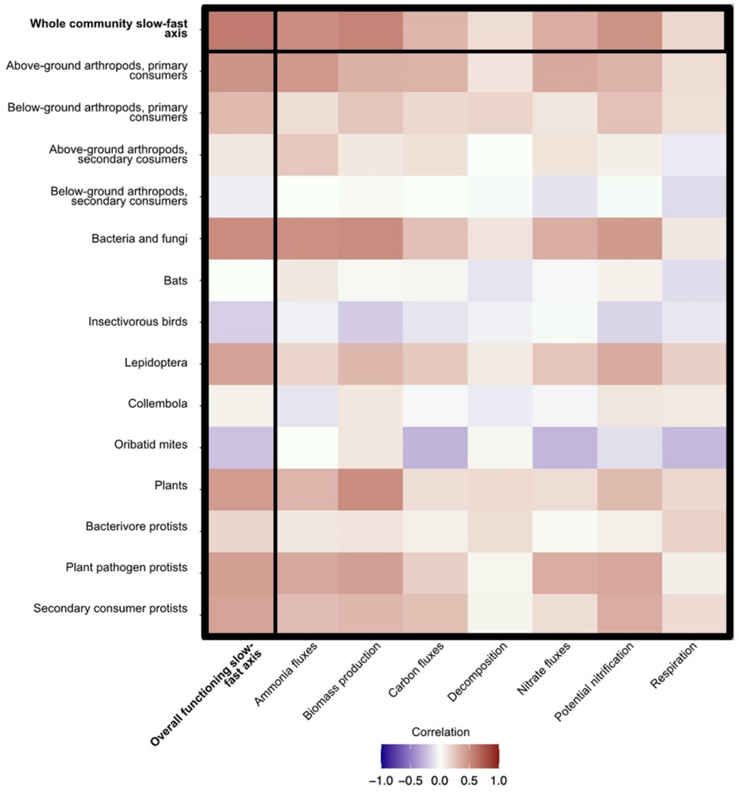

Figure S 7. Identification of guild-level slow-fast axes. **Analyses excluded all size- and body mass-related traits.** Icons were acquired and adapted from Phylopic.org (artists: M. Dahirel, B. Lang, M. Crook, J. A. Venter, H. H. T. Prins, D. A. Balfour, R. Slotow, T. M. Keesey, A. A. Farke, Y. Wong, G. Monger).

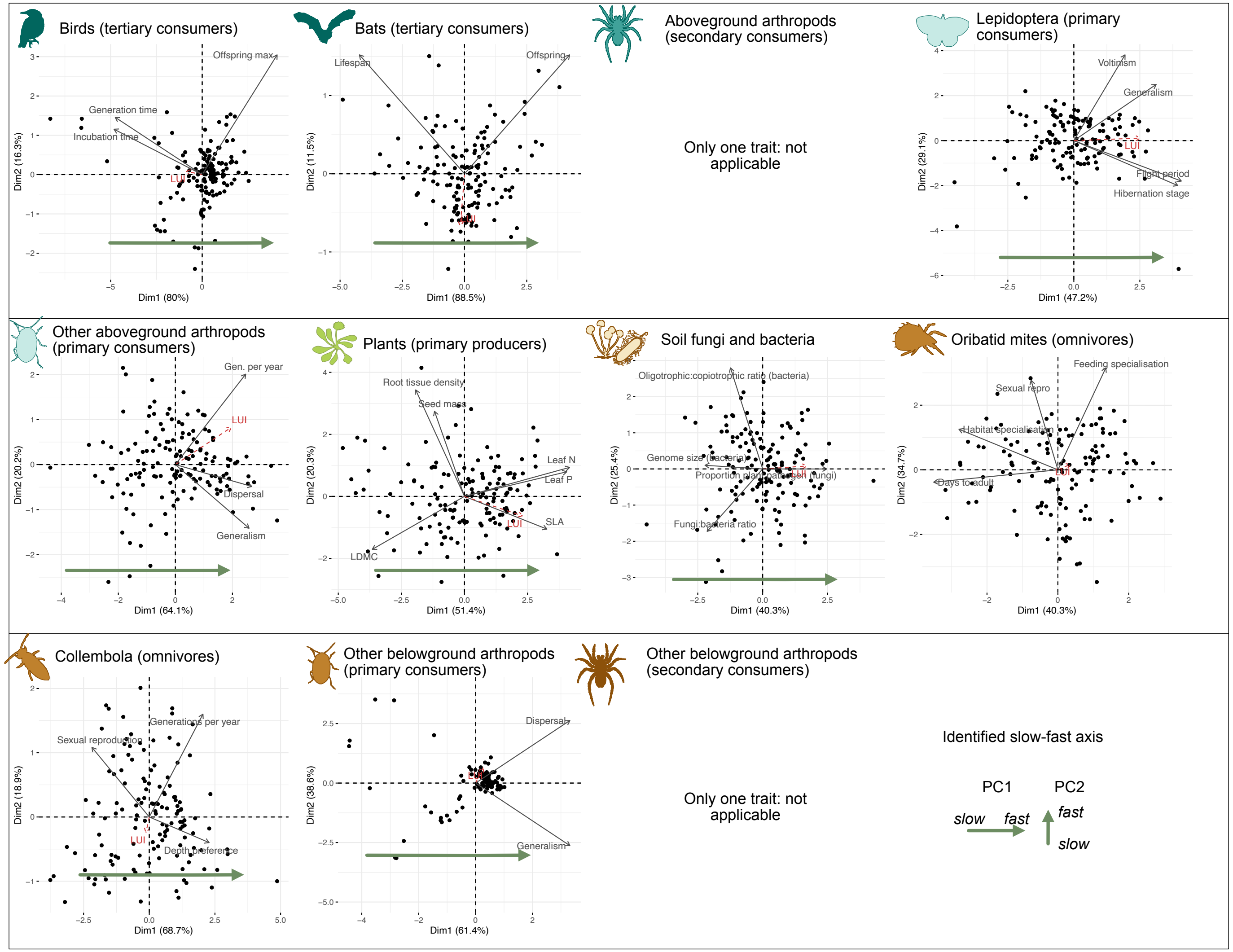

Figure S 8. Synchronised slow-fast trait response of individual guilds is strongly related to land-use intensity. **Analyses excluded all size- and body mass-related traits**. The variables included in the PCA are the slow-fast axes of each guild. Land-use intensity, added as a supplementary variable, was strongly associated with axis 1. Belowground guilds are shown in brown, aboveground guilds in blue. Icons were acquired and adapted from Phylopic.org (artists: M. Dahirel, B. Lang, M. Crook, J. A. Venter, H. H. T. Prins, D. A. Balfour, R. Slotow, T. M. Keesey, A. A. Farke, Y. Wong, G. Monger).

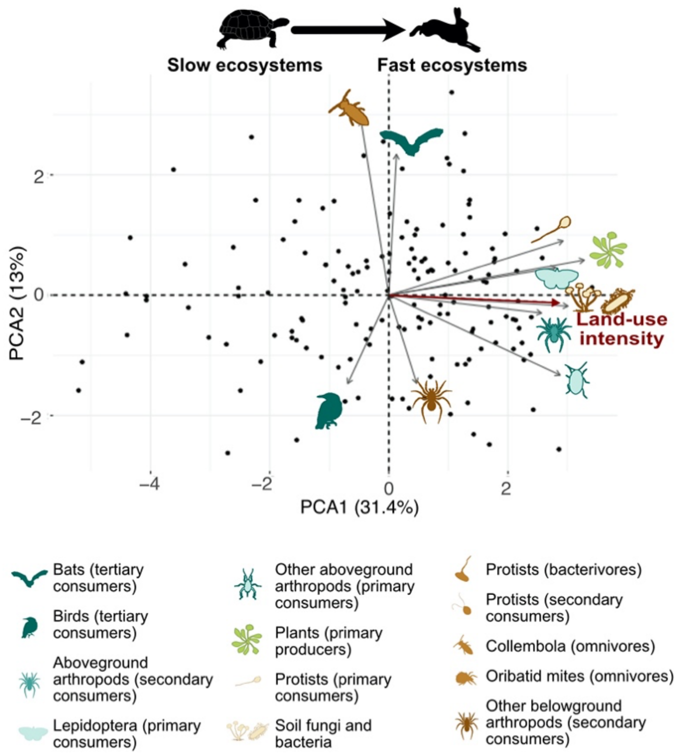

Figure S 9. Direct and trophically mediated effects of land-use intensity on the slow-fast axis of different trophic levels**. Analyses excludes size- and body mass- related traits.** a. Full SEMs including all guilds. Two independent models were fitted for below- and aboveground guilds; plants being included in both. b. Average direct, indirect and total LUI effects on each trophic level (averaged from the full SEM). c. Decreasing direct, indirect and total LUI effects with trophic level. Each dot represents the estimated effect (+/- standard error) of an individual guild in the full SEM. Icons were acquired and adapted from Phylopic.org (artists: M. Dahirel, B. Lang, M. Crook, J. A. Venter, H. H. T. Prins, D. A. Balfour, R. Slotow, T. M. Keesey, A. A. Farke, Y. Wong, G. Monger).

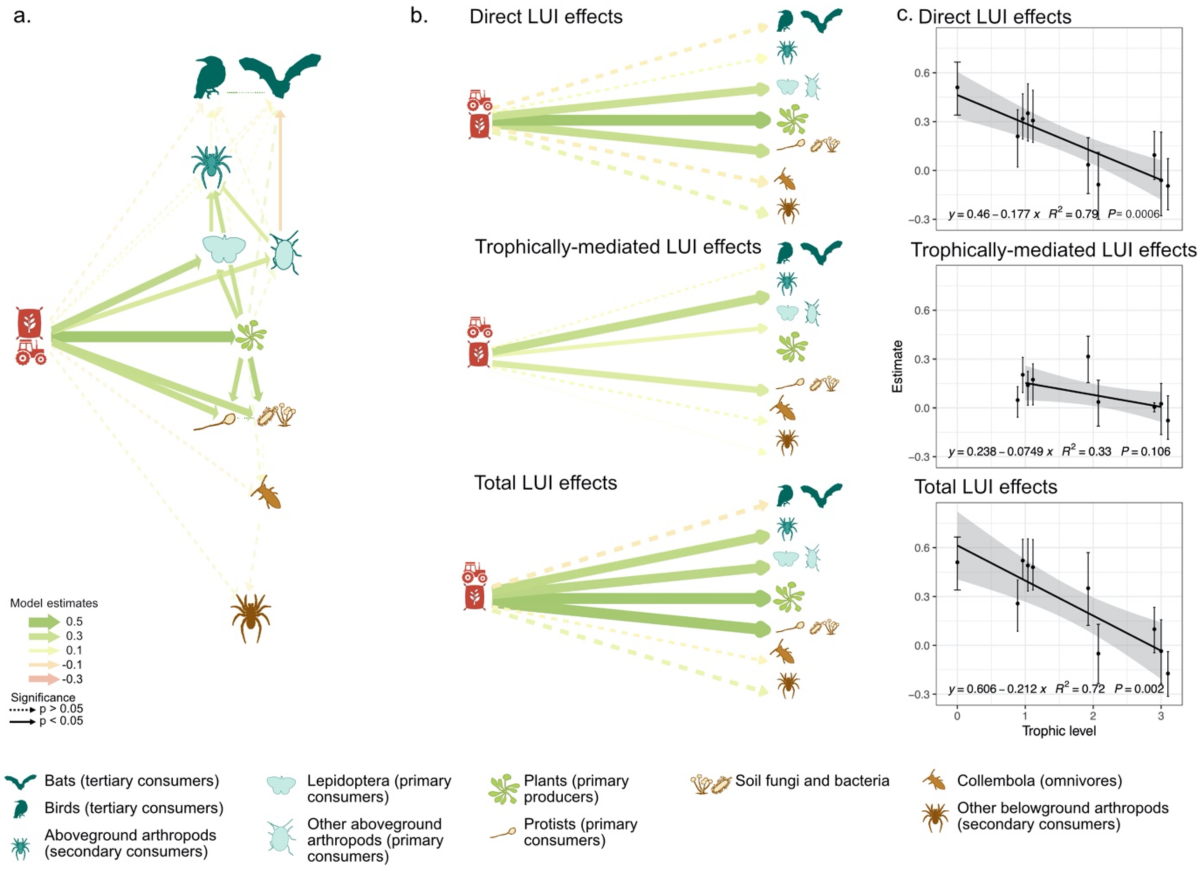

Figure S 10. Identification of guild-level slow-fast axes. In contrast to the results shown as Figure 2**, community-level trait data (CWM) was not weighted by taxa abundance.** Icons were acquired and adapted from Phylopic.org (artists: M. Dahirel, B. Lang, M. Crook, J. A. Venter, H. H. T. Prins, D. A. Balfour, R. Slotow, T. M. Keesey, A. A. Farke, Y. Wong, G. Monger).

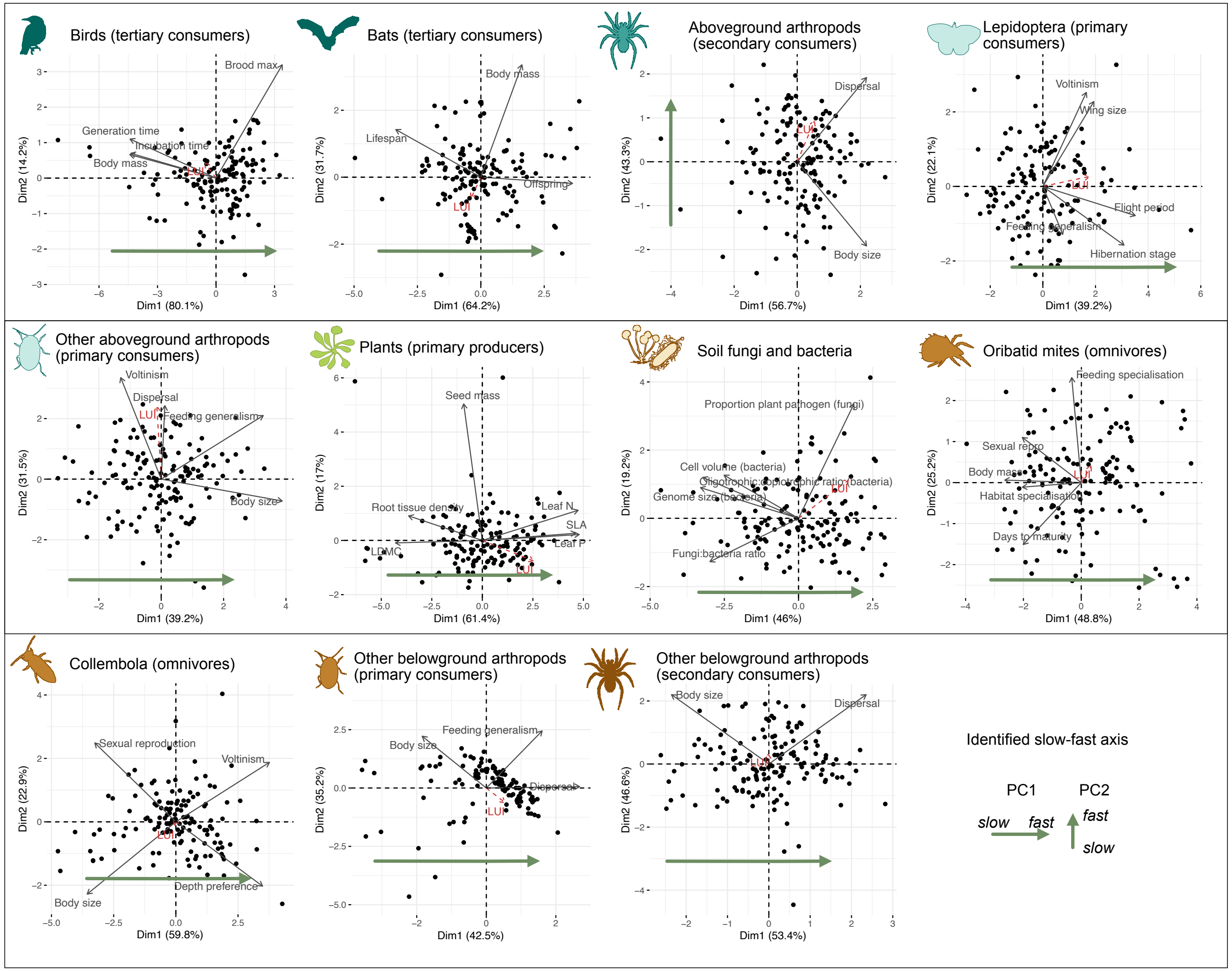

Figure S 11. Correlation between guild-level PCA axes or single traits and land use intensity. Three axes were retained when more than two traits were available; otherwise only two were retained. For protists, only one trait was available, and the correlation between the CWM of this trait and LUI is shown. The fast-slow axis is always the first PC axis, except for arthropod (primary and secondary consumers) above-ground, for which it was PC axis 2. The axes were transformed were needed (inversed sign) so that higher axis values indicate "faster" strategies. P-values were corrected for false detection rates (***: P < 0.001, **: P < 0.01, *: P< 0.05, n.s.: P > 0.05). **Community-level trait data (CWM) was not weighted by taxa abundance.**

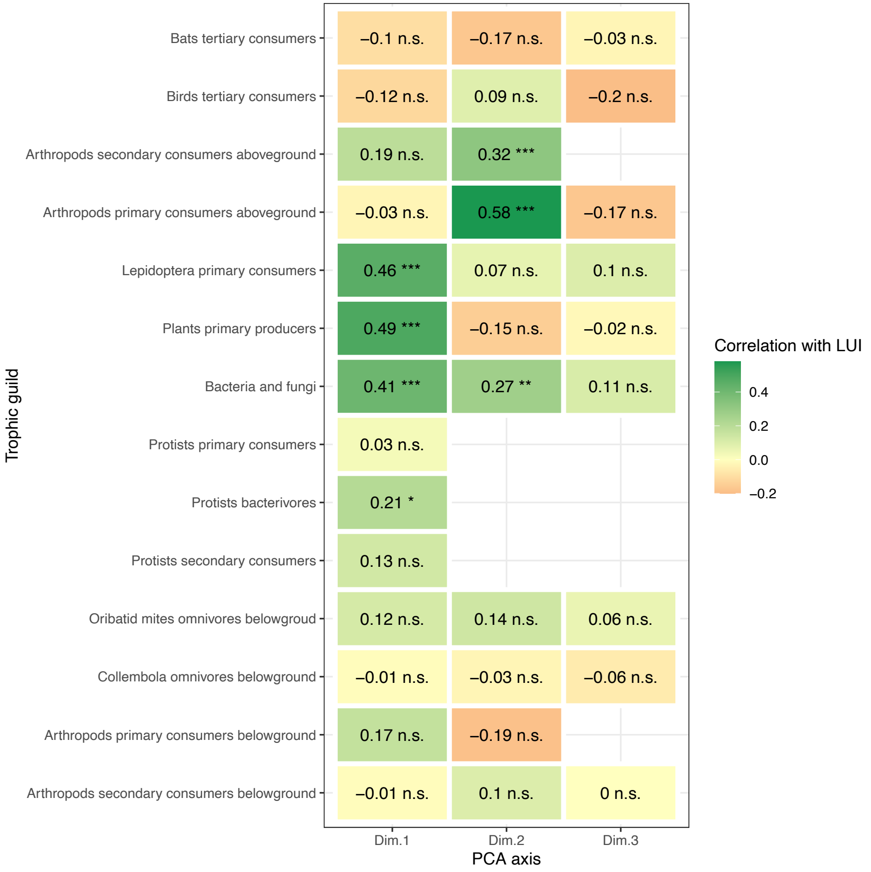

Figure S 12. Synchronised slow-fast trait response of individual guilds is strongly related to land-use intensity. **Community-level trait data (CWM) was not weighted by taxa abundance**. The variables included in the PCA are the slow-fast axes of each guild. Land-use intensity, added as a supplementary variable, was strongly associated with axis 1. Belowground guilds are shown in brown, aboveground guilds in blue. Icons were acquired and adapted from Phylopic.org (artists: M. Dahirel, B. Lang, M. Crook, J. A. Venter, H. H. T. Prins, D. A. Balfour, R. Slotow, T. M. Keesey, A. A. Farke, Y. Wong, G. Monger).

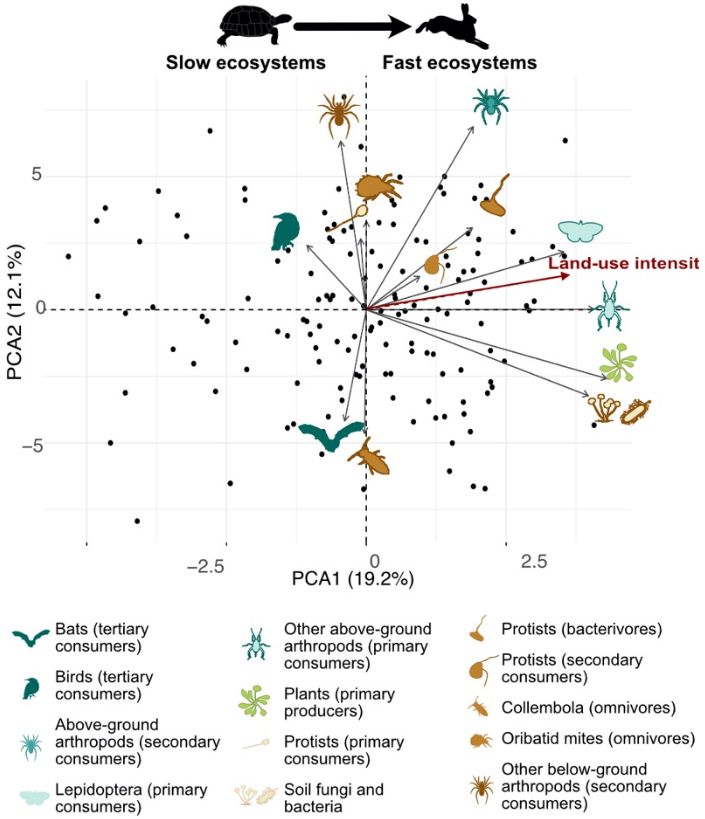

Figure S 13. Direct and trophically mediated effects of land-use intensity on the slow-fast axis of different trophic levels. **Community-level trait data (CWM) was not weighted by taxa abundance**. a. Full SEMs including all guilds. Two independent models were fitted for below- and aboveground guilds; plants being included in both. b. Average direct, indirect and total LUI effects on each trophic level (averaged from the full SEM). c. Decreasing direct, indirect and total LUI effects with trophic level. Each dot represents the estimated effect (+/- standard error) of an individual guild in the full SEM. Icons were acquired and adapted from Phylopic.org (artists: M. Dahirel, B. Lang, M. Crook, J. A. Venter, H. H. T. Prins, D. A. Balfour, R. Slotow, T. M. Keesey, A. A. Farke, Y. Wong, G. Monger).

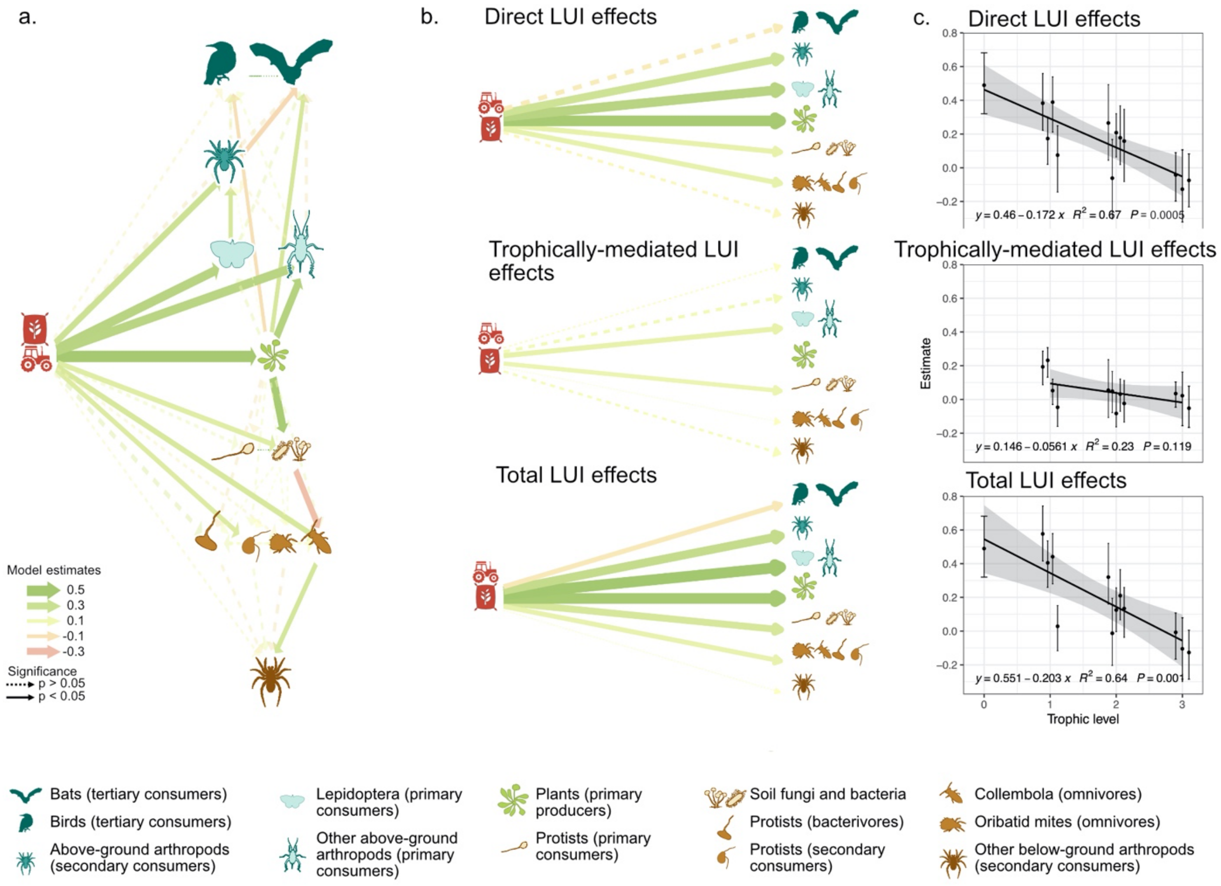

Figure S 14. Direct and indirect links between land- use intensity, functional traits slow-fast axis and ecosystem function slow-fast axis. **In contrast to Figure 6, community-level trait data (CWM) was not weighted by taxa abundance.**

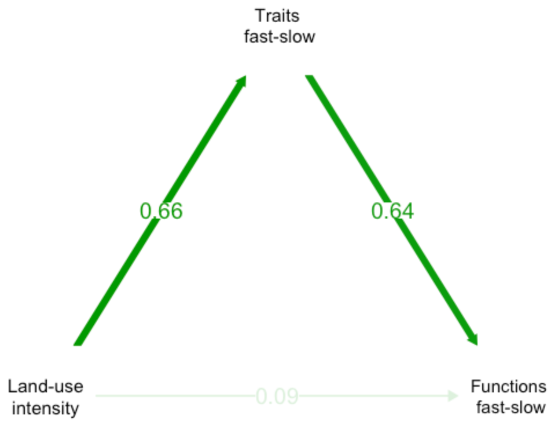

Figure S 15. Identification of guild-level slow-fast axes. **Community-level traits (CWM) were not corrected for environmental covariates**. Different colors show the different regions of the Exploratories. Icons were acquired and adapted from Phylopic.org (artists: M. Dahirel, B. Lang, M. Crook, J. A. Venter, H. H. T. Prins, D. A. Balfour, R. Slotow, T. M. Keesey, A. A. Farke, Y. Wong, G. Monger).

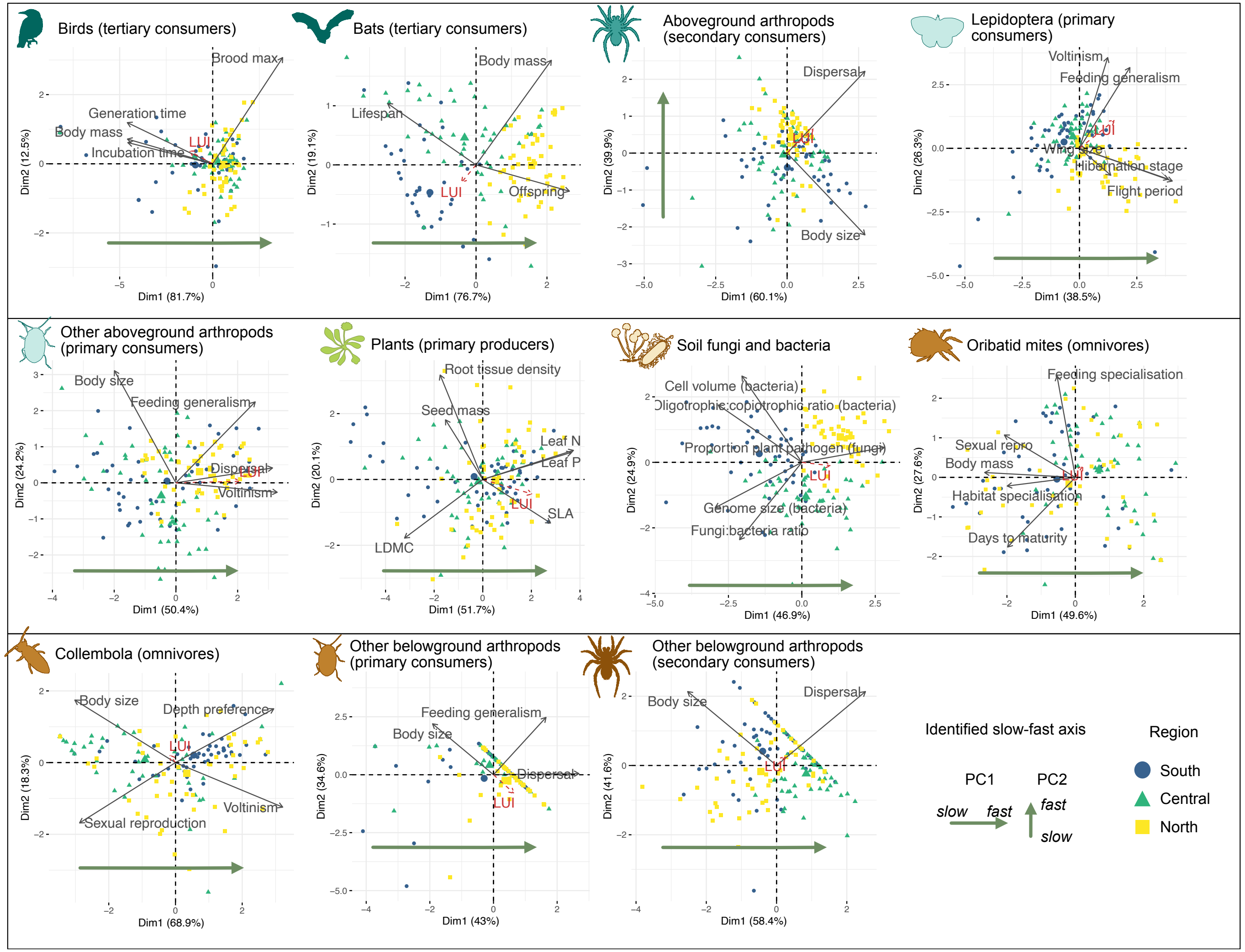

Figure S 16. Correlation between guild-level PCA axes or single traits and land use intensity. Three axes were retained when more than two traits were available; otherwise only two were retained. For protists, only one trait was available, and the correlation between the CWM of this trait and LUI is shown. The fast-slow axis is always the first PC axis, except for arthropod (secondary consumers) above-ground, for which it was PC axis 2. The axes were transformed were needed (inversed sign) so that higher axis values indicate "faster" strategies. P-values were corrected for false detection rates (***: P < 0.001, **: P < 0.01, *: P< 0.05, n.s.: P > 0.05). **Community-level traits (CWM) were not corrected for environmental covariates**.

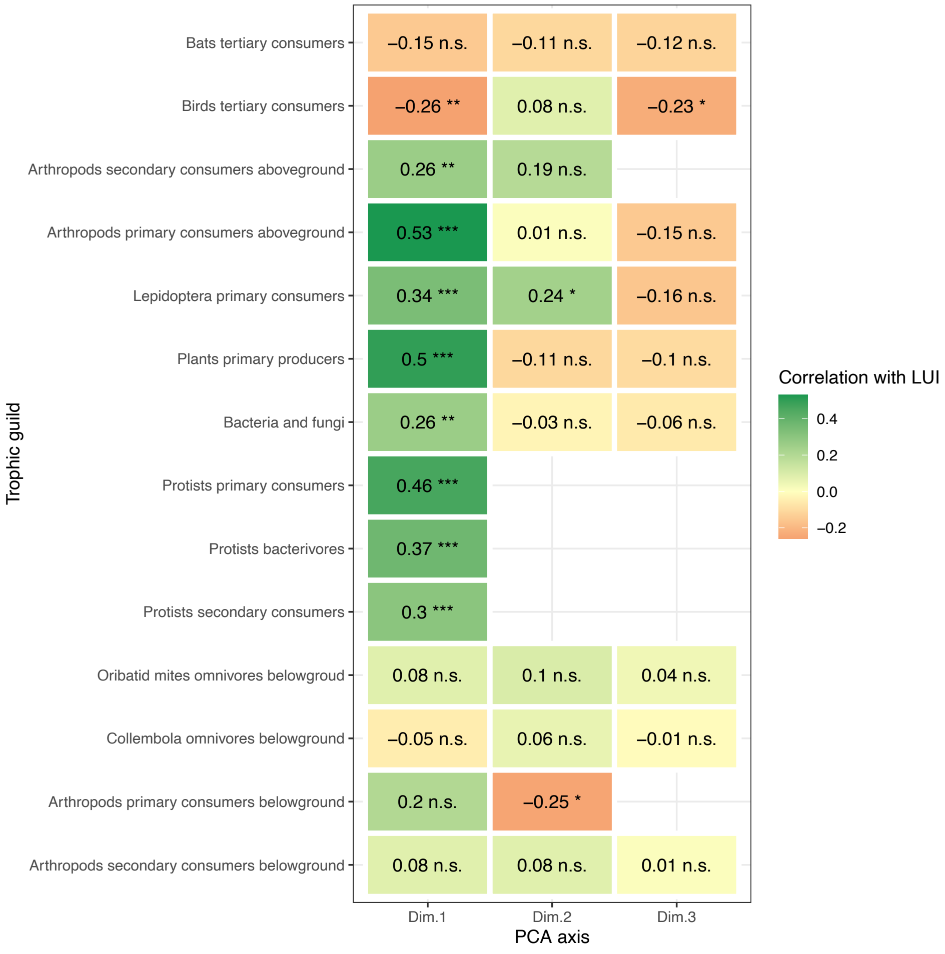

Figure S 17. Synchronised slow-fast trait response of individual guilds is strongly related to land-use intensity. **Community-level traits (CWM) were not corrected for environmental covariates**. The variables included in the PCA are the slow-fast axes of each guild. Land-use intensity, added as a supplementary variable, was strongly associated with axis 1. Belowground guilds are shown in brown, aboveground guilds in blue. Icons were acquired and adapted from Phylopic.org (artists: M. Dahirel, B. Lang, M. Crook, J. A. Venter, H. H. T. Prins, D. A. Balfour, R. Slotow, T. M. Keesey, A. A. Farke, Y. Wong, G. Monger).

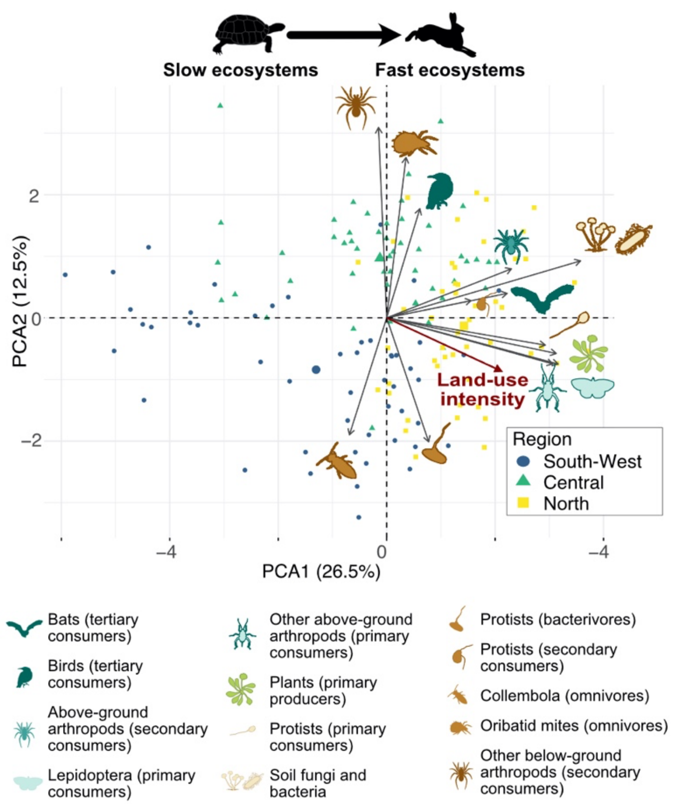

Figure S 18. Direct and trophically mediated effects of land-use intensity on the slow-fast axis of different trophic levels**. Community-level traits (CWM) were not corrected for environmental covariates.** a. Full SEMs including all guilds. Two independent models were fitted for below- and aboveground guilds; plants being included in both. b. Average direct, indirect and total LUI effects on each trophic level (averaged from the full SEM). c. Decreasing direct, indirect and total LUI effects with trophic level. Each dot represents the estimated effect (+/- standard error) of an individual guild in the full SEM. Icons were acquired and adapted from Phylopic.org (artists: M. Dahirel, B. Lang, M. Crook, J. A. Venter, H. H. T. Prins, D. A. Balfour, R. Slotow, T. M. Keesey, A. A. Farke, Y. Wong, G. Monger).

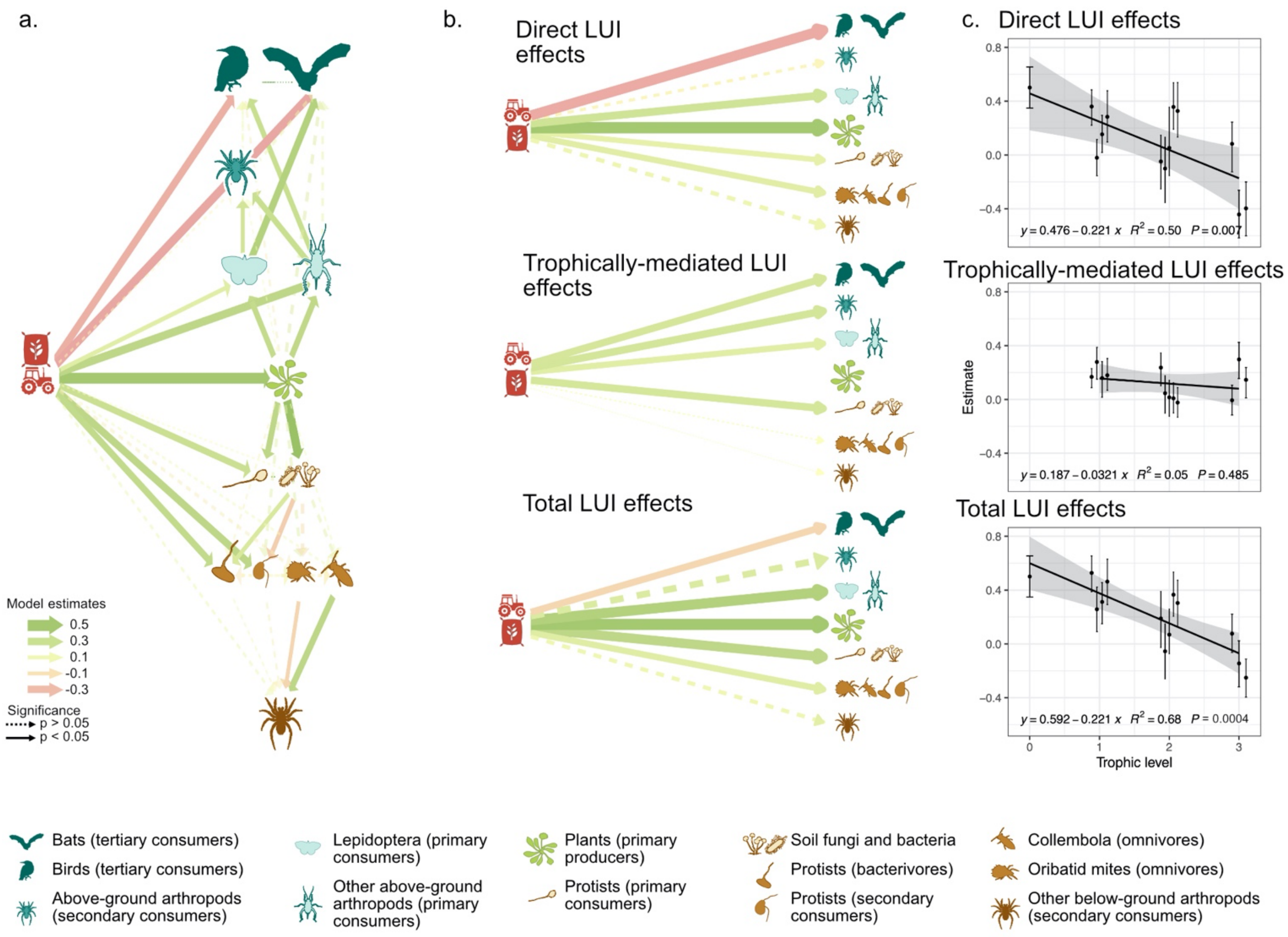

Figure S 19. Identification of ecosystem functions slow-fast axis. **Functions were not corrected for environmental covariates.** For all functions, high values are expected to be related to “fast” ecosystem functioning. Opposite response of dung decomposition compared to others is explained by the negative effect of land-use intensity (especially mowing) on the abundance and activity of dung beetles (Frank et al., 2017).

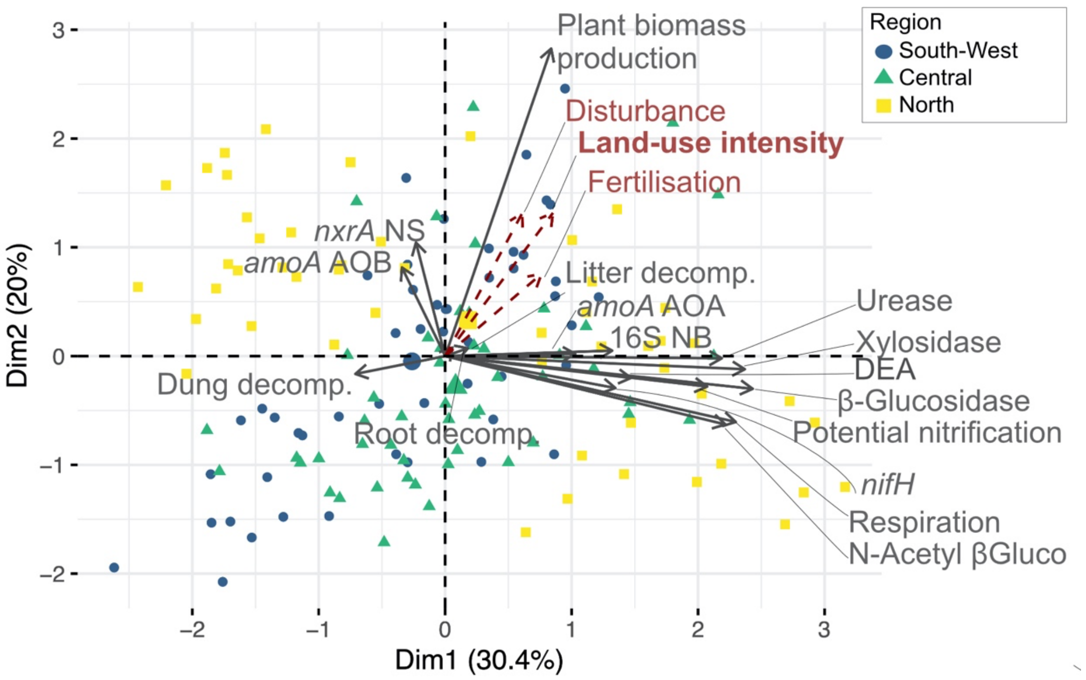

Figure S 20. Direct and indirect links between land- use intensity, functional traits slow-fast axis and ecosystem function slow-fast axis. **In contrast to Figure 6, functions and community-level trait data were not corrected for environmental covariates.**

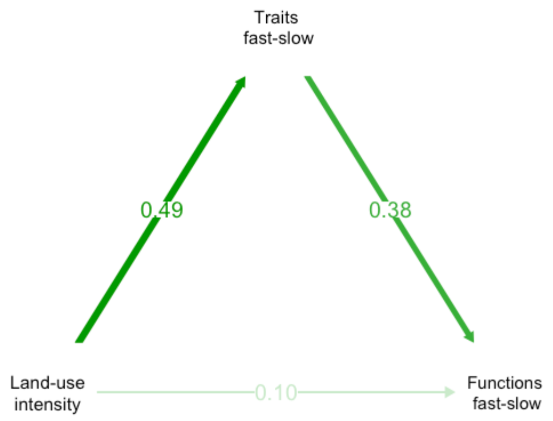

Supplementary references

Barnett, S.E., Youngblut, N.D., Koechli, C.N., Buckley, D.H., 2021. Multisubstrate DNA stable isotope probing reveals guild structure of bacteria that mediate soil carbon cycling. Proc. Natl. Acad. Sci. 118, e2115292118. https://doi.org/10.1073/pnas.2115292118

Bergmann, J., Weigelt, A., Plas, F. van der, Laughlin, D.C., Kuyper, T.W., Guerrero-Ramirez, N., Valverde-Barrantes, O.J., Bruelheide, H., Freschet, G.T., Iversen, C.M., Kattge, J., McCormack, M.L., Meier, I.C., Rillig, M.C., Roumet, C., Semchenko, M., Sweeney, C.J., Ruijven, J. van, York, L.M., Mommer, L., 2020. The fungal collaboration gradient dominates the root economics space in plants. Sci. Adv. https://doi.org/10.1126/sciadv.aba3756

Birkhofer, K., Diekötter, T., Meub, C., Stötzel, K., Wolters, V., 2015a. Optimizing arthropod predator conservation in permanent grasslands by considering diversity components beyond species richness. Agric. Ecosyst. Environ. 211, 65–72. https://doi.org/10.1016/j.agee.2015.05.014

Birkhofer, K., Gossner, M.M., Diekötter, T., Drees, C., Ferlian, O., Maraun, M., Scheu, S., Weisser, W.W., Wolters, V., Wurst, S., Zaitsev, A.S., Smith, H.G., 2017. Land-use type and intensity differentially filter traits in above- and below-ground arthropod communities. J. Anim. Ecol. 86, 511–520. https://doi.org/10.1111/1365-2656.12641

Birkhofer, K., Smith, H.G., Weisser, W.W., Wolters, V., Gossner, M.M., 2015b. Land-use effects on the functional distinctness of arthropod communities. Ecography 38, 889–900. https://doi.org/10.1111/ecog.01141

Boeddinghaus, R.S., Marhan, S., Berner, D., Boch, S., Fischer, M., Hölzel, N., Kattge, J., Klaus, V.H., Kleinebecker, T., Oelmann, Y., Prati, D., Schäfer, D., Schöning, I., Schrumpf, M., Sorkau, E., Kandeler, E., Manning, P., 2019. Plant functional trait shifts explain concurrent changes in the structure and function of grassland soil microbial communities. J. Ecol. 107, 2197–2210. https://doi.org/10.1111/1365-2745.13182

Börschig, C., Klein, A.-M., von Wehrden, H., Krauss, J., 2013. Traits of butterfly communities change from specialist to generalist characteristics with increasing land-use intensity. Basic Appl. Ecol. 547–554. https://doi.org/10.1016/j.baae.2013.09.002

Cavalier-Smith, T., 1980. r- and K-tactics in the evolution of protist developmental systems: Cell and genome size, phenotype diversifying selection, and cell cycle patterns. Biosystems 12, 43–59. https://doi.org/10.1016/0303-2647(80)90037-4

Chauvat, M., Wolters, V., Dauber, J., 2007. Response of collembolan communities to land-use change and grassland succession. Ecography 30, 183–192. https://doi.org/10.1111/j.0906-7590.2007.04888.x

Chisté, M.N., Mody, K., Kunz, G., Gunczy, J., Blüthgen, N., 2018. Intensive land use drives small-scale homogenization of plant- and leafhopper communities and promotes generalists. Oecologia 186, 529–540. https://doi.org/10.1007/s00442-017-4031-0

de Vries, F.T., Hoffland, E., van Eekeren, N., Brussaard, L., Bloem, J., 2006. Fungal/bacterial ratios in grasslands with contrasting nitrogen management. Soil Biol. Biochem. 38, 2092–2103. https://doi.org/10.1016/j.soilbio.2006.01.008

de Vries, F.T., Manning, P., Tallowin, J.R.B., Mortimer, S.R., Pilgrim, E.S., Harrison, K.A., Hobbs, P.J., Quirk, H., Shipley, B., Cornelissen, J.H.C., Kattge, J., Bardgett, R.D., 2012. Abiotic drivers and plant traits explain landscape-scale patterns in soil microbial communities. Ecol. Lett. 15, 1230–1239. https://doi.org/10.1111/j.1461-0248.2012.01844.x

Devereux, C., Whittingham, M., KREBS, J., Fernández-Juricic, E., Vickery, J., 2006. What attracts birds to newly mown pasture? Decoupling the action of mowing from the provision of short swards. Ibis 148, 302–306. https://doi.org/10.1111/j.1474-919X.2006.00533.x

Díaz, S., Kattge, J., Cornelissen, J.H.C., Wright, I.J., Lavorel, S., Dray, S., Reu, B., Kleyer, M., Wirth, C., Colin Prentice, I., Garnier, E., Bönisch, G., Westoby, M., Poorter, H., Reich, P.B., Moles, A.T., Dickie, J., Gillison, A.N., Zanne, A.E., Chave, J., Joseph Wright, S., Sheremet'ev, S.N., Jactel, H., Baraloto, C., Cerabolini, B., Pierce, S., Shipley, B., Kirkup, D., Casanoves, F., Joswig, J.S., Günther, A., Falczuk, V., Rüger, N., Mahecha, M.D., Gorné, L.D., 2016. The global spectrum of plant form and function. Nature 529, 167–171. https://doi.org/10.1038/nature16489

Endara, M.-J., Coley, P.D., 2011. The resource availability hypothesis revisited: a meta-analysis. Funct. Ecol. 25, 389–398. https://doi.org/10.1111/j.1365-2435.2010.01803.x

Fierer, N., Leff, J.W., Adams, B.J., Nielsen, U.N., Bates, S.T., Lauber, C.L., Owens, S., Gilbert, J.A., Wall, D.H., Caporaso, J.G., 2012. Cross-biome metagenomic analyses of soil microbial communities and their functional attributes. Proc. Natl. Acad. Sci. 109, 21390–21395. https://doi.org/10.1073/pnas.1215210110

Fiore-Donno, A.M., Richter-Heitmann, T., Bonkowski, M., 2020. Contrasting Responses of Protistan Plant Parasites and Phagotrophs to Ecosystems, Land Management and Soil Properties. Front. Microbiol. 11.

Frank, K., Hülsmann, M., Assmann, T., Schmitt, T., Blüthgen, N., 2017. Land use affects dung beetle communities and their ecosystem service in forests and grasslands. Agric. Ecosyst. Environ. 243, 114–122. https://doi.org/10.1016/j.agee.2017.04.010

Frawley, B.J., Best, L.B., 1992. Effects of mowing on breeding bird abundance and species composition in alfalfa fields. Biol. Conserv. 60, 222. https://doi.org/10.1016/0006-3207(92)91269-X

Grime, J.P., 1979. Plant Strategies and Vegetation Processes. John Wiley & Sons Ltd, Chichester.

Hanson, H.I., Palmu, E., Birkhofer, K., Smith, H.G., Hedlund, K., 2016. Agricultural Land Use Determines the Trait Composition of Ground Beetle Communities. PLOS ONE 11, e0146329. https://doi.org/10.1371/journal.pone.0146329

Hodgson, J.G., Montserrat-Martí, G., Charles, M., Jones, G., Wilson, P., Shipley, B., Sharafi, M., Cerabolini, B.E.L., Cornelissen, J.H.C., Band, S.R., Bogard, A., Castro-Díez, P., Guerrero-Campo, J., Palmer, C., Pérez-Rontomé, M.C., Carter, G., Hynd, A., Romo-Díez, A., de Torres Espuny, L., Royo Pla, F., 2011. Is leaf dry matter content a better predictor of soil fertility than specific leaf area? Ann. Bot. 108, 1337–1345. https://doi.org/10.1093/aob/mcr225

Konstantinidis, K.T., Tiedje, J.M., 2004. Trends between gene content and genome size in prokaryotic species with larger genomes. Proc. Natl. Acad. Sci. 101, 3160–3165. https://doi.org/10.1073/pnas.0308653100

Lavorel, S., Garnier, E., 2002. Predicting changes in community composition and ecosystem functioning from plant traits: revisiting the Holy Grail. Funct. Ecol. 16, 545–556. https://doi.org/10.1046/j.1365-2435.2002.00664.x

Le Provost, G., Thiele, J., Westphal, C., Penone, C., Allan, E., Neyret, M., van der Plas, F., Ayasse, M., Bardgett, R.D., Birkhofer, K., Boch, S., Bonkowski, M., Buscot, F., Feldhaar, H., Gaulton, R., Goldmann, K., Gossner, M.M., Klaus, V.H., Kleinebecker, T., Krauss, J., Renner, S., Scherreiks, P., Sikorski, J., Baulechner, D., Blüthgen, N., Bolliger, R., Börschig, C., Busch, V., Chisté, M., Fiore-Donno, A.M., Fischer, M., Arndt, H., Hoelzel, N., John, K., Jung, K., Lange, M., Marzini, C., Overmann, J., Paŝalić, E., Perović, D.J., Prati, D., Schäfer, D., Schöning, I., Schrumpf, M., Sonnemann, I., Steffan-Dewenter, I., Tschapka, M., Türke, M., Vogt, J., Wehner, K., Weiner, C., Weisser, W., Wells, K., Werner, M., Wolters, V., Wubet, T., Wurst, S., Zaitsev, A.S., Manning, P., 2021. Contrasting responses of above- and belowground diversity to multiple components of land-use intensity. Nat. Commun. 12, 3918. https://doi.org/10.1038/s41467-021-23931-1

Leff, J.W., Jones, S.E., Prober, S.M., Barberán, A., Borer, E.T., Firn, J.L., Harpole, W.S., Hobbie, S.E., Hofmockel, K.S., Knops, J.M.H., McCulley, R.L., La Pierre, K., Risch, A.C., Seabloom, E.W., Schütz, M., Steenbock, C., Stevens, C.J., Fierer, N., 2015. Consistent responses of soil microbial communities to elevated nutrient inputs in grasslands across the globe. Proc. Natl. Acad. Sci. 112, 10967–10972. https://doi.org/10.1073/pnas.1508382112

Lekberg, Y., Arnillas, C.A., Borer, E.T., Bullington, L.S., Fierer, N., Kennedy, P.G., Leff, J.W., Luis, A.D., Seabloom, E.W., Henning, J.A., 2021. Nitrogen and phosphorus fertilization consistently favor pathogenic over mutualistic fungi in grassland soils. Nat. Commun. 12, 3484. https://doi.org/10.1038/s41467-021-23605-y

Liu, X., Zhang, L., Huang, M., Zhou, S., 2021. Plant diversity promotes soil fungal pathogen richness under fertilization in an alpine meadow. J. Plant Ecol. 14, 323–336. https://doi.org/10.1093/jpe/rtaa099

Lüftenegger, G., Foissner, W., Adam, H., 1985. r-and K-selection in soil ciliates: a field and experimental approach. Oecologia 66, 574–579. https://doi.org/10.1007/BF00379352

MacDonald, M.A., 2006. The indirect effects of increased nutrient inputs on birds in the UK: a review 220.

Petersen, H., 1980. Population dynamic and metaboliccharacterization of Collembola species in a beechforest ecosystem., in: Soil Biology Asrelated to Land Use Practices. Presented at the roceedings of the VIIinternational soil zoology colloquium of the ISSS, D indal, D.L. (Ed), Syracuse, pp. 806-833Pe.

Pianka, E.R., 1970. On r- and K-Selection. Am. Nat. 104, 592–597.

Sibly, R.M., Witt, C.C., Wright, N.A., Venditti, C., Jetz, W., Brown, J.H., 2012. Energetics, lifestyle, and reproduction in birds. Proc. Natl. Acad. Sci. 109, 10937–10941. https://doi.org/10.1073/pnas.1206512109

Simons, N.K., Weisser, W.W., Gossner, M.M., 2016. Multi-taxa approach shows consistent shifts in arthropod functional traits along grassland land-use intensity gradient. Ecology 15-0616.1. https://doi.org/10.1890/15-0616.1

Socher, S.A., Prati, D., Boch, S., Müller, J., Klaus, V.H., Hölzel, N., Fischer, M., 2012. Direct and productivity-mediated indirect effects of fertilization, mowing and grazing on grassland species richness. J. Ecol. 100, 1391–1399. https://doi.org/10.1111/j.1365-2745.2012.02020.x

Weigelt, A., Mommer, L., Andraczek, K., Iversen, C.M., Bergmann, J., Bruelheide, H., Fan, Y., Freschet, G.T., Guerrero-Ramírez, N.R., Kattge, J., Kuyper, T.W., Laughlin, D.C., Meier, I.C., van der Plas, F., Poorter, H., Roumet, C., van Ruijven, J., Sabatini, F.M., Semchenko, M., Sweeney, C.J., Valverde-Barrantes, O.J., York, L.M., McCormack, M.L., 2021. An integrated framework of plant form and function: The belowground perspective. New Phytol. nph.17590. https://doi.org/10.1111/nph.17590

Westoby, M., Gillings, M.R., Madin, J.S., Nielsen, D.A., Paulsen, I.T., Tetu, S.G., 2021. Trait dimensions in bacteria and archaea compared to vascular plants. Ecol. Lett. ele.13742. https://doi.org/10.1111/ele.13742

Wright, I.J., Reich, P.B., Westoby, M., Ackerly, D.D., Baruch, Z., Bongers, F., Cavender-Bares, J., Chapin, T., Cornelissen, J.H., Diemer, M., 2004. The worldwide leaf economics spectrum. Nature 428, 821–827.
