# Supplementary material for: A slow-fast trait continuum at the whole community level in relation to land-use intensification
Source: Nat Commun. 2024 Feb 10;15:1251. doi: 10.1038/s41467-024-45113-5 (PMC10858939; doi:10.1038/s41467-024-45113-5)
Supplement: Supplementary file 3 — Reporting Summary [file 41467_2024_45113_MOESM3_ESM.pdf]

nature portfolio

Corresponding author(s): Margot Neyret

Last updated by author(s): Dec 8, 2023

Reporting Summary

Nature Portfolio wishes to improve the reproducibility of the work that we publish. This form provides structure for consistency and transparency in reporting. For further information on Nature Portfolio policies, see our Editorial Policies and the Editorial Policy Checklist.

Statistics

For all statistical analyses, confirm that the following items are present in the figure legend, table legend, main text, or Methods section.

n/a	Confirmed	
☐	☒	The exact sample size (*n*) for each experimental group/condition, given as a discrete number and unit of measurement
☐	☒	A statement on whether measurements were taken from distinct samples or whether the same sample was measured repeatedly
☐	☒	The statistical test(s) used AND whether they are one- or two-sided<br>*Only common tests should be described solely by name; describe more complex techniques in the Methods section.*
☐	☒	A description of all covariates tested
☐	☒	A description of any assumptions or corrections, such as tests of normality and adjustment for multiple comparisons
☐	☒	A full description of the statistical parameters including central tendency (e.g. means) or other basic estimates (e.g. regression coefficient) AND variation (e.g. standard deviation) or associated estimates of uncertainty (e.g. confidence intervals)
☐	☒	For null hypothesis testing, the test statistic (e.g. *F*, *t*, *r*) with confidence intervals, effect sizes, degrees of freedom and *P* value noted<br>*Give P values as exact values whenever suitable.*
☒	☐	For Bayesian analysis, information on the choice of priors and Markov chain Monte Carlo settings
☒	☐	For hierarchical and complex designs, identification of the appropriate level for tests and full reporting of outcomes
☐	☒	Estimates of effect sizes (e.g. Cohen's *d*, Pearson's *r*), indicating how they were calculated

Our web collection on statistics for biologists contains articles on many of the points above.

Software and code

Policy information about availability of computer code

Data collection	Mothur v. 39.5: Protist data. Avisoft SAS Lab Pro, Version 5.0.24: Bat data
Data analysis	All the data analyses were conducted using the R software v. 4.0.3. The full code to replicate the analyses is available on github (Doi: 10.5281/zenodo.10286644)<br><br>R packages used: ade4 (v 1.7-22), betapart (v 1.6), car (v 3.1-1), data.table (v 1.14.8), data.table (v 1.14.8), dplyr (v 1.1.0), factoextra (v 1.0.7), FactoMineR (v 2.7), FD (v 1.0-12.1), fishmethods (v 1.12-0), forcats (v 1.0.0), ggcorrplot (v 0.1.4), ggfortify (v 0.4.15), ggh4x (v 0.2.3), ggnetwork (v 0.5.12), ggpmisc (v 0.5.2), ggraph (v 2.1.0), Hmisc (v 5.0-1), kableExtra (v 1.3.4), lavaan (v 0.6-14), mice (v 3.15.0), parameters (v 0.21.3), phyloseq (v 1.42.0), plyr (v 1.1.0), readr (v 2.1.4), readxl (v 1.4.2), reshape2 (v 1.4.4), rsq (v 2.5), scales (v 1.2.1), semPlot (v 1.1.6), semTable (v 1.8), stringr (v 1.5.0), taxize (v 0.9.100), Taxonstand (v 2.4), textclean (v 0.9.3), tidygraph (v 1.2.3), traitdataform (v 0.6.8), vegan (v 2.6-4), viridis (v 0.6.2)

For manuscripts utilizing custom algorithms or software that are central to the research but not yet described in published literature, software must be made available to editors and reviewers. We strongly encourage code deposition in a community repository (e.g. GitHub). See the Nature Portfolio guidelines for submitting code & software for further information.

Data

Policy information about availability of data

All manuscripts must include a data availability statement. This statement should provide the following information, where applicable:

- Accession codes, unique identifiers, or web links for publicly available datasets
- A description of any restrictions on data availability
- For clinical datasets or third party data, please ensure that the statement adheres to our policy

This work is based on data elaborated by several projects of the Biodiversity Exploratories program (DFG Priority Program 1374). Most datasets from the Biodiversity Exploratories are publicly available in the Biodiversity Exploratories Information System (http://doi.org/10.17616/R32P9Q). The Community Weighted Mean data generated in this study have been deposited under accession code 31516 https://www.bexis.uni-jena.de/ddm/data/Showdata/31516. The raw trait and abundance, and ecosystem functions datasets are detailed below, many of which are publicly available. To give data owners and collectors time to perform their analysis the Biodiversity Exploratories' data and publication policy includes by default an embargo period of three years from the end of data collection/data assembly. Both the CWM and remaining raw data datasets are thus available under restricted access, access can be obtained by contacting data owners (listed on Bexis). At the end oft he embargo period these datasets will be made publicly available via the same data repository.

Full list of used datasets (both from the Biodiversity Exploratories and external, previously published datasets):

Apostolakis, A., Schöning, I., Schrumpf, M., Klötzing, T. & Trumbore, S. (2020) MinSoil 2018-19: Soil respiration in forests and grasslands. v4. Biodiversity Exploratories Information System. Dataset. 2020. https://www.bexis.uni-jena.de/ddm/data/Showdata/26908.

Bakewell, A.T., Davis, K.E., Freckleton, R.P., Isaac, N.J.B. & Mayhew, P.J. (2020) Comparing Life Histories across Taxonomic Groups in Multiple Dimensions: How Mammal-Like Are Insects? The American Naturalist. 195 (1), 70–81. doi:10.1086/706195.

Barbara Stempfhuber, Gerhard Welzl, Tesfaye Wubet, Ingo Schöning, Sven Marhan, François Buscot, Ellen Kandeler, & Michael Schloter (2014) Drivers for ammonia-oxidation along a land-use gradient in grassland soils. Soil Biology and Biochemistry. 69, 179–186. doi:10.1016/j.soilbio.2013.11.007.

Baulechner, D., Wolters, V., John, K. & Zaytsev, A. (n.d.) Acari abundance (Oribatida to species level) on all 150 grassland EPs from 2019. v4. Biodiversity Exploratories Information System. Dataset. https://www.bexis.uni-jena.de/ddm/data/Showdata/27406.

Baulechner, D., Wolters, V., Zaytsev, A. & John, K. (2020) Collembolan species abundance on all 150 grassland EPs from 2019. v4. Biodiversity Exploratories Information System. Dataset. 2020. https://www.bexis.uni-jena.de/ddm/data/Showdata/27007.

Berner, D., Marhan, S., Keil, D., Poll, C., Schuetzenmeister, A., Piepho, H.-P. & Kandeler, E. (2011) Land-use intensity modifies spatial distribution and function of soil microorganisms in grasslands. Pedobiologia. 54, 341–351. doi:10.1016/j.pedobi.2011.08.001.

Bird, J.P., Martin, R., Akçakaya, H.R., Gilroy, J., Burfield, I.J., Garnett, S.T., Symes, A., Taylor, J., Şekercioğlu, Ç.H. & Butchart, S.H.M. (2020) Generation lengths of the world's birds and their implications for extinction risk. Conservation Biology. 34 (5), 1252–1261. doi:10.1111/cobi.13486.

Blüthgen, N., Mangels, J. & Schneider, F. (2018) Morphological traits of moths (Standardized). v2. Biodiversity Exploratories Information System. Dataset. 2018. https://www.bexis.uni-jena.de/ddm/data/Showdata/23926.

Boch, S., Mueller, J., Socher, S., Prati, D. & Fischer, M. (2017) Measurement of biomass (2009, all grassland EPs). v2. Biodiversity Exploratories Information System. Dataset. 2017. https://www.bexis.uni-jena.de/ddm/data/Showdata/16209.

Boeddinghaus, R.S., Marhan, S., Berner, D., Boch, S., Fischer, M., Hölzel, N., Kattge, J., Klaus, V.H., Kleinebecker, T., Oelmann, Y., Prati, D., Schäfer, D., Schöning, I., Schrumpf, M., Sorkau, E., Kandeler, E. & Manning, P. (2019) Plant functional trait shifts explain concurrent changes in the structure and function of grassland soil microbial communities F. Vries (ed.). Journal of Ecology. 107 (5), 2197–2210. doi:10.1111/1365-2745.13182.

Bolliger, R., Prati, D. & Fischer, M. (2021) Vegetation Records for Grassland EPs, 2008 - 2020. v2. Biodiversity Exploratories Information System. Dataset. 2021. https://www.bexis.uni-jena.de/ddm/data/Showdata/27386.

Börschig, C., Klein, A.-M., von Wehrden, H. & Krauss, J. (2013) Traits of butterfly communities change from specialist to generalist characteristics with increasing land-use intensity. Basic and Applied Ecology. 547–554. doi:https://doi.org/10.1016/j.baae.2013.09.002.

Börshig, C. & Krauss, J. (2011) Diversity and abundance of day active butterflies and day-active moths along a land use intensity gradient (2008). v2. Biodiversity Exploratories Information System. Dataset. 2011. https://www.bexis.uni-jena.de/ddm/data/Showdata/12526.

Buscot, F., Goldmann, K. & Wubet, T. (2021) Abundant soil fungi on all 150 grassland EPs (from Soil Sampling Campain 2011; Illumina MiSeq) - ASV abundances. v3. Biodiversity Exploratories Information System. Dataset. 2021. https://www.bexis.uni-jena.de/ddm/data/Showdata/26470.

Buscot, F., Goldmann, K. & Wubet, T. (2020) Abundant soil fungi on all 150 grassland EPs (from Soil Sampling Campain 2014; Illumina MiSeq) - ASV abundances. v3. Biodiversity Exploratories Information System. Dataset. 2020. https://www.bexis.uni-jena.de/ddm/data/Showdata/26471.

Conenna, I., Santini, L., Rocha, R., Monadjem, A., Cabeza, M. & Russo, D. (2021) Global patterns of functional trait variation along aridity gradients in bats. Global Ecology and Biogeography. 30 (5), 1014–1029. doi:10.1111/geb.13278.

Cook, P.M., Tordoff, G.M., Davis, A.M., Parsons, M.S., Dennis, E.B., Fox, R., Botham, M.S. & Bourn, N.A.D. (2021) Traits data for the butterflies and macro-moths of Great Britain and Ireland. NERC EDS Environmental Information Data Centre. doi:https://doi.org/10.5285/5b5a13b6-2304-47e3-9c9d-35237d1232c6.

Dumack, K., Fiore-Donno, A.M., Bass, D. & Bonkowski, M. (2020) Making sense of environmental sequencing data: Ecologically important functional traits of the protistan groups Cercozoa and Endomyxa (Rhizaria). Molecular Ecology Resources. 20 (2), 398–403. doi:10.1111/1755-0998.13112.

Fiore-Donno, A.M. & Bonkowski, M. (2019a) Cercozoa and Endomyxa (Rhizaria, protists), Illumina Sequences, all EPs, grassland and forest, 2011. v3. Biodiversity Exploratories Information System. Dataset. 2019. https://www.bexis.uni-jena.de/ddm/data/Showdata/24426.

Fiore-Donno, A.M. & Bonkowski, M. (2019b) Cercozoa and Endomyxa (Rhizaria, protists), Illumina Sequences, all EPs, grassland and forest, 2017. v3. Biodiversity Exploratories Information System. Dataset. 2019. https://www.bexis.uni-jena.de/ddm/data/Showdata/24466.

Fischer, M. & Grassein, F. (2015) Litter decomposition (2013, all grassland EPs). v3. Biodiversity Exploratories Information System. Dataset. 2015. https://www.bexis.uni-jena.de/ddm/data/Showdata/18926.

Fischer, M., Schäfer, D., Boch, S. & Prati, D. (2017) Vegetation Records for 150 Grassland EPs in 2016, Header Data without Species Identities (incl. biomass). v2. Biodiversity Exploratories Information System. Dataset. 2017. https://www.bexis.uni-jena.de/ddm/data/Showdata/21187.

Frank, K. & Blüthgen, N. (2017) Decomposition (dung removal in g on 300 EPs, season 2014/2015), Taxon: dung beetles (Invertebrates, Scarabaeoidea) - Dungwebs. v3. Biodiversity Exploratories Information System. Dataset. 2017. https://www.bexis.uni-jena.de/ddm/data/Showdata/21206.

Frank, K., Hülsmann, M., Assmann, T., Schmitt, T. & Blüthgen, N. (2017) Land use affects dung beetle communities and their ecosystem service in forests and grasslands. Agriculture, Ecosystems & Environment. 243, 114–122. doi:10.1016/j.agee.2017.04.010.

Frostegård, Å., Bååth, E. & Tunlio, A. (1993) Shifts in the structure of soil microbial communities in limed forests as revealed by phospholipid fatty acid analysis. Soil Biology and Biochemistry. 25 (6), 723–730. doi:10.1016/0038-0717(93)90113-P.

Frostegård, Å., Tunlid, A. & Bååth, E. (1991) Microbial biomass measured as total lipid phosphate in soils of different organic content. Journal of Microbiological Methods. 14 (3), 151–163. doi:10.1016/0167-7012(91)90018-L.

Frostegård, Å., Tunlid, A. & Bååth, E. (1993) Phospholipid Fatty Acid Composition, Biomass, and Activity of Microbial Communities from Two Soil Types Experimentally Exposed to Different Heavy Metals. Applied and Environmental Microbiology. doi:10.1128/aem.59.11.3605-3617.1993.

Goldmann, K., Buscot, F. & Wubet, T. (2018) Abundant fungi on all EPs (from Soil Sampling Campain 2011) - merged grassland and forest data: taxonomic look-up

table. Version 3. Biodiversity Exploratories Information System. (Dataset). 2018. https://www.bexis.uni-jena.de/ddm/data/Showdata/24306.
Grogan, P. (1998) Co2 Flux Measurement Using Soda Lime: Correction for Water Formed During Co2 Adsorption. Ecology. 79 (4), 1467–1468. doi:10.1890/0012-9658(1998)079[1467:CFMUSL]2.0.CO;2.
Hänsel, F., Forteva, S., Wöllauer, S. & Nauss, T. (2019) Öffentlich verfügbare Klimadaten der Exploratorien / Open Climate Data of the Exploratories Project. v4. Biodiversity Exploratories Information System. Dataset. 2019. https://www.bexis.uni-jena.de/tcd/PublicClimateData/Index.
Hoffmann, H., Schloter, M. & Wilke, B.-M. (2007) Microscale-scale measurement of potential nitrification rates of soil aggregates. Biology and Fertility of Soils. 44 (2), 411–413. doi:10.1007/s00374-007-0227-5.
Jung, K. & Tschapka, M. (2016a) Bat activity in all Exploratories, summer 2009, using acoustic monitoring. v2. Biodiversity Exploratories Information System. Dataset. 2016. https://www.bexis.uni-jena.de/ddm/data/Showdata/19849.
Jung, K. & Tschapka, M. (2016b) Bat activity in all Exploratories, summer 2010, using acoustic monitoring. v2. Biodiversity Exploratories Information System. Dataset. 2016. https://www.bexis.uni-jena.de/ddm/data/Showdata/19850.
Kandeler, E., Berner, D., Marhan, S. & Boeddinghaus, R.S. (2017a) Soil enzyme activities of all grassland EPs, soil sampling campaign (SSC) 2011, SCALEMIC. v4. Biodiversity Exploratories Information System. Dataset. 2017. https://www.bexis.uni-jena.de/ddm/data/Showdata/20246.
Kandeler, E., Marhan, S., Berner, D. & Boeddinghaus, R.S. (2017b) Microbial soil properties of all grassland EPs, soil sampling campaign (SSC) 2011, SCALEMIC. v3. Biodiversity Exploratories Information System. Dataset. 2017. https://www.bexis.uni-jena.de/ddm/data/Showdata/20250.
Kandeler, E., Marhan, S., Berner, D. & Boeddinghaus, R.S. (2017c) Microbial soil properties of all grassland EPs, soil sampling campaign (SSC) 2014, SCALEMIC. v3. Biodiversity Exploratories Information System. Dataset. 2017. https://www.bexis.uni-jena.de/ddm/data/Showdata/20251.
Kattge, J., Díaz, S., Lavorel, S., Prentice, I.C., Leadley, P., et al. (2011) TRY – a global database of plant traits. Global Change Biology. 17 (9), 2905–2935. doi:10.1111/j.1365-2486.2011.02451.x.
Keil, D., Niklaus, P.A., von Riedmatten, L.R., Boeddinghaus, R.S., Dormann, C.F., Scherer-Lorenzen, M., Kandeler, E. & Marhan, S. (2015) Effects of warming and drought on potential N2O emissions and denitrifying bacteria abundance in grasslands with different land-use. FEMS Microbiology Ecology. 91 (7), fiv066. doi:10.1093/femsec/fiv066.
Keith, H. & Wong, S.C. (2006) Measurement of soil CO2 efflux using soda lime absorption: both quantitative and reliable. Soil Biology and Biochemistry. 38 (5), 1121–1131. doi:10.1016/j.soilbio.2005.09.012.
Kovacevic, V., Schulz, S. & Schloter, M. (2019) Nitrifiers Abundances at Grassland EPs. v4. Biodiversity Exploratories Information System. Dataset. 2019. https://www.bexis.uni-jena.de/ddm/data/Showdata/21547.
Le Provost, G., Manning, P., Schöning, I., Schrumpf, M., Weisser, W.W., Lorenzen, K., Vogt, J., Thiele, J., Westphal, C. & Dewenter, I.S. (2021) Aggregated environmental and land-use covariates of the 150 grassland EPs used in ‘Contrasting responses of above- and belowground diversity to multiple components of land-use intensity’. v5. Biodiversity Exploratories Information System. Dataset. 2021. https://www.bexis.uni-jena.de/ddm/data/Showdata/31018.
Madin, J.S., Nielsen, D.A., Brbic, M., Corkrey, R., Danko, D., et al. (2020) A synthesis of bacterial and archaeal phenotypic trait data. Scientific Data. 7 (1), 170. doi:10.1038/s41597-020-0497-4.
Mangels, J. & Blüthgen, N. (2017) Life history traits of moths. v2. Biodiversity Exploratories Information System. Dataset. 2017. https://www.bexis.uni-jena.de/ddm/data/Showdata/21228.
Mangels, J., Fiedler, K., Schneider, F.D. & Blüthgen, N. (2017) Diversity and trait composition of moths respond to land-use intensification in grasslands: generalists replace specialists. Biodiversity and Conservation. 26 (14), 3385–3405. doi:10.1007/s10531-017-1411-z.
Marx, M.-C., Wood, M. & Jarvis, S.C. (2001) A microplate fluorimetric assay for the study of enzyme diversity in soils. Soil Biology and Biochemistry. 33 (12), 1633–1640. doi:10.1016/S0038-0717(01)00079-7.
McMahon, B.J., Doyle, S., Gray, A., Kelly, S.B.A. & Redpath, S.M. (2020) European bird declines: Do we need to rethink approaches to the management of abundant generalist predators? Journal of Applied Ecology. 57 (10), 1885–1890. doi:10.1111/1365-2664.13695.
Middleton-Welling, J., Dapporto, L., García-Barros, E., Wiemers, M., Nowicki, P., Plazio, E., Bonelli, S., Zaccagno, M., Šašić, M., Liparova, J., Schweiger, O., Harpke, A., Musche, M., Settele, J., Schmucki, R. & Shreeve, T. (2020) A new comprehensive trait database of European and Maghreb butterflies, Papilionoidea. Scientific Data. 7 (1), 351. doi:10.1038/s41597-020-00697-7.
Neff, F., Resch, M.C., Marty, A., Rolley, J.D., Schütz, M., Risch, A.C. & Gossner, M.M. (2020) Long-term restoration success of insect herbivore communities in semi-natural grasslands: a functional approach. Ecological Applications. 30 (6). doi:doi.org/10.1002/eap.2133.
Neyret, M., Lachaise, T., van Kleunen, M., Bergmann, J. & Manning, P. (2021) Community-weighted mean for plant below-ground traits - 300 forest and grassland EPs - 2008-2018. v3. Biodiversity Exploratories Information System. Dataset. 2021. https://www.bexis.uni-jena.de/ddm/data/Showdata/27608.
Nickel, H. & Remane, R. (2002) Check list of the planthoppers and leafhoppers of Germany, with notes on food plants, diet width, life cycles, geographic range and conservation status (Hemiptera, Fulgoromorpha and Cicadomorpha). Beiträge zur Zikadenkunde. 5.
Obermann, J. & Sikorski, J. (2019) 16S rRNA gene (V3 region) RNA-based analysis of microbial soil communities at sequence variant level in 148 grassland EP plots, using QIIME2-based bioinformatics, 2011. v2. Biodiversity Exploratories Information System. Dataset. 2019. https://www.bexis.uni-jena.de/ddm/data/Showdata/24866.
Ollivier, J., Schacht, D., Kindler, R., Groeneweg, J., Engel, M., Wilke, B.-M., Kleineidam, K. & Schloter, M. (2013) Effects of repeated application of sulfadiazine-contaminated pig manure on the abundance and diversity of ammonia and nitrite oxidizers in the root-rhizosphere complex of pasture plants under field conditions. Frontiers in Microbiology. 4, 22. doi:10.3389/fmicb.2013.00022.
Penone, C., Renner, S., Teuscher, M. & Fischer, M. (2022) Bird traits for all species in the Exploratories EPs. 2022. https://www.bexis.uni-jena.de/ddm/data/Showdata/31368.
Pigot, A.L., Sheard, C., Miller, E.T., Bregman, T.P., Freeman, B.G., Roll, U., Seddon, N., Trisos, C.H., Weeks, B.C. & Tobias, J.A. (2020) Macroevolutionary convergence connects morphological form to ecological function in birds. Nature Ecology & Evolution. 4 (2), 230–239. doi:10.1038/s41559-019-1070-4.
Prati, D., Fischer, M., Minker, J. & Schmitt, B. (2013) Measurement of biomass (2013, all grassland EPs). v2. Biodiversity Exploratories Information System. Dataset. 2013. https://www.bexis.uni-jena.de/ddm/data/Showdata/16786.
Renner, S.C. & Hoesel, W. van (2017) Ecological and Functional Traits in 99 Bird Species over a Large-Scale Gradient in Germany. Data. 2 (2), 12. doi:10.3390/data2020012.
Ruess, L. & Chamberlain, P.M. (2010) The fat that matters: Soil food web analysis using fatty acids and their carbon stable isotope signature. Soil Biology and Biochemistry. 42 (11), 1898–1910. doi:10.1016/j.soilbio.2010.07.020.
Saulich, A. & Musolin, D. (2021) Seasonal Development of Plant Bugs (Heteroptera, Miridae): Subfamily Mirinae, Tribe Stenodemini. Entomological Review. 101, 147–161. doi:10.1134/S0013873821020019.
Schäfer, D., Fischer, M., Klaus, V. & Busch, V. (2016a) Vegetation Records for 150 Grassland EPs in 2014, Header Data without Species Identities (incl. biomass). v2. Biodiversity Exploratories Information System. Dataset. 2016. https://www.bexis.uni-jena.de/ddm/data/Showdata/19807.
Schäfer, D., Fischer, M., Klaus, V. & Busch, V. (2016b) Vegetation Records for 150 Grassland EPs in 2015, Header Data without Species Identities (incl. biomass). v2. Biodiversity Exploratories Information System. Dataset. 2016. https://www.bexis.uni-jena.de/ddm/data/Showdata/19809.
Schäfer, D., Prati, D. & Fischer, M. (2018) Vegetation Records for 150 Grassland EPs in 2017, Header Data without Species Identities (incl. biomass). v2. Biodiversity Exploratories Information System. Dataset. 2018. https://www.bexis.uni-jena.de/ddm/data/Showdata/23486.
Schall, O. & Petermann, R. (2014) National Report on Bat Conservation in the Federal Republic of Germany 2010-2013. https://www.eurobats.org/sites/default/files/documents/pdf/National_Reports/Inf.MoP7_.20-National%20Implementation%20Report%20of%20Germany.pdf.
Schinner, F., Öhlinger, R., Kandeler, E. & Margesin, R. (1996) Methods in Soil Biology. 1st edition. Berlin Heidelberg, Springer.
Schmitt, B., Prati, D. & Fischer, M. (2011) Measurement of biomass (2010, all grassland EPs). v2. Biodiversity Exploratories Information System. Dataset. 2011.

https://www.bexis.uni-jena.de/ddm/data/Showdata/12706.
Schmitt, B., Prati, D., Fischer, M. & Minker, J. (2012a) Measurement of biomass (2011, all grassland EPs). v3. Biodiversity Exploratories Information System. Dataset. 2012. https://www.bexis.uni-jena.de/ddm/data/Showdata/14346.
Schmitt, B., Prati, D., Fischer, M. & Minker, J. (2012b) Measurement of biomass (2012, all grassland EPs). v2. Biodiversity Exploratories Information System. Dataset. 2012. https://www.bexis.uni-jena.de/ddm/data/Showdata/14986.
Schöning, I., Heublein, J., Klötzing, T., Schrumpf, M. & Trumbore, S. (2013) MinSoil 2011 - Soil Texture. v3. Biodiversity Exploratories Information System. Dataset. 2013. https://www.bexis.uni-jena.de/ddm/data/Showdata/14686.
Schöning, I., Solly, E., Klötzing, T., Schrumpf, M. & Trumbore, S. (2015a) Mineral soil pH values of all experimental plots (EP) of the Biodiversity Exploratories project from 2011, Soil (core project). v8. Biodiversity Exploratories Information System. Dataset. 2015. https://www.bexis.uni-jena.de/ddm/data/Showdata/14447.
Schöning, I., Ying Gan, H., Heublein, J., Klötzing, T., Schrumpf, M. & Trumbore, S. (2015b) MinSoil 2014 - Soil pH. v3. Biodiversity Exploratories Information System. Dataset. 2015. https://www.bexis.uni-jena.de/ddm/data/Showdata/19067.
Sikorski, J., Overmann, J. & Marzini, C. (2019) 16S rRNA gene (V3 region) RNA-based analysis of microbial soil communities at sequence variant level in 150 grassland EP plots, using QIIME2-based bioinformatics, 2014. v3. Biodiversity Exploratories Information System. Dataset. 2019. https://www.bexis.uni-jena.de/ddm/data/Showdata/25066.
Smith, M.S. & Tiedje, J.M. (1979) Phases of denitrification following oxygen depletion in soil. Soil Biology and Biochemistry. 11 (3), 261–267. doi:10.1016/0038-0717(79)90071-3.
Solly, E. & Schöning, I. (2013) MinSoil 2011 Root decomposition. v2. Biodiversity Exploratories Information System. Dataset. 2013. https://www.bexis.uni-jena.de/ddm/data/Showdata/16666.
Staab, M., Simons, N.K., Gossner, M.M., Weisser, W.W. & Blüthgen, N. (2022) Body size and life-history traits of arthropod species. v7. Biodiversity Exploratories Information System. Dataset. 2022. https://www.bexis.uni-jena.de/ddm/data/Showdata/31122.
Stempfhuber, B. & Schloter, M. (2017) Abundance of nitrogen fixing microbes and ammonia oxidizers in grassland. v2. Biodiversity Exploratories Information System. Dataset. 2017. https://www.bexis.uni-jena.de/ddm/data/Showdata/13986.
Stempfhuber, B. & Schloter, M. (2016) Nitrogen Pools and Potential Nitrification taken at Soil Sampling Campaign 2014. v3. Biodiversity Exploratories Information System. Dataset. 2016. https://www.bexis.uni-jena.de/ddm/data/Showdata/19847.
Teuscher, M. & Fischer, M. (2021) Bird survey and trait data on all grassland and forest EPs 2018. v3. Biodiversity Exploratories Information System. Dataset. 2021. https://www.bexis.uni-jena.de/ddm/data/Showdata/25306.
Tobias, J.A., Sheard, C., Pigot, A.L., Devenish, A.J.M., Yang, J., et al. (2022) AVONET: morphological, ecological and geographical data for all birds. Ecology Letters. 25 (3), 581–597. doi:10.1111/ele.13898.
Tschapka, M., Renner, S. & Jung, K. (2017a) Bird survey data 2008, all 300 EPs. v2. Biodiversity Exploratories Information System. Dataset. 2017. https://www.bexis.uni-jena.de/ddm/data/Showdata/21446.
Tschapka, M., Renner, S. & Jung, K. (2017b) Bird survey data 2009, all 300 EPs. v2. Biodiversity Exploratories Information System. Dataset. 2017. https://www.bexis.uni-jena.de/ddm/data/Showdata/21447.
Tschapka, M., Renner, S. & Jung, K. (2017c) Bird survey data 2010, all 300 EPs. v2. Biodiversity Exploratories Information System. Dataset. 2017. https://www.bexis.uni-jena.de/ddm/data/Showdata/21448.
Tschapka, M., Renner, S. & Jung, K. (2017d) Bird survey data 2011, all 300 EPs. v2. Biodiversity Exploratories Information System. Dataset. 2017. https://www.bexis.uni-jena.de/ddm/data/Showdata/21449.
Weisser, W., Gossner, M., Pasalic, E., Lange, M., Türke, M., Gallenberger, I., Simons, N. & Staab, M. (2019) Sweep net samples from grasslands since 2008: Araneae, Coleoptera, Hemiptera, Orthoptera. v4. Biodiversity Exploratories Information System. Dataset. 2019. https://www.bexis.uni-jena.de/ddm/data/Showdata/21969.
Wilkinson, G.S. & South, J.M. (2002) Life history, ecology and longevity in bats. Aging Cell. 1 (2), 124–131. doi:10.1046/j.1474-9728.2002.00020.x.

Research involving human participants, their data, or biological material

Policy information about studies with human participants or human data. See also policy information about sex, gender (identity/presentation), and sexual orientation and race, ethnicity and racism.

Reporting on sex and gender	Not applicable
Reporting on race, ethnicity, or other socially relevant groupings	Not applicable
Population characteristics	Not applicable
Recruitment	Not applicable
Ethics oversight	Not applicable

Note that full information on the approval of the study protocol must also be provided in the manuscript.

Field-specific reporting

Please select the one below that is the best fit for your research. If you are not sure, read the appropriate sections before making your selection.

☐ Life sciences ☐ Behavioural & social sciences ☒ Ecological, evolutionary & environmental sciences

For a reference copy of the document with all sections, see nature.com/documents/nr-reporting-summary-flat.pdf

Ecological, evolutionary & environmental sciences study design

All studies must disclose on these points even when the disclosure is negative.

Study description	The study was conducted as part of the long-term Biodiversity Exploratories project (www.biodiversity-exploratories.de). Data was collected in 150 grassland plots in three regions of Germany: the Schwabische Alb plateau and UNESCO Biosphere Reserve in southwestern<br>Germany; the Hainich National Park and surrounding areas in central Germany (both are hilly regions with calcareous<br>bedrock) and the UNESCO Biosphere Reserve Schorfheide-Chorin in the post-glacial lowlands of north-eastern Germany. The three regions differ in climate, geology and topography, but each is characterised by a gradient of grassland land-use intensity that is typical for large parts of temperate Europe44. In each region, 50 plots (50 m x 50 m) were chosen in secondary wet, mesic and dry grasslands by stratified random sampling from a total of 500 candidate plots on which initial vegetation, soil and land-use surveys were conducted. This ensured that plots covered the whole range of land-use intensities and management types, while minimising confounding factors such as spatial position or soil type.<br>At each plot, we measured the species or family richness of these trophic groups using standard methodology (data available for 111 to 150 plots depending on the group and trait considered). The 150 agricultural grassland<br>plots vary strongly in their local land-use intensity (quantified as a compound indices based on grazing, mowing and fertilization intensity), and are situated in landscapes of varying complexity and management history. In summary, there are 150 replicates, nested within three regions, each containing 50 replicates.
Research sample	The sample unit is the community of a grassland plot, considering 14 above and belowground trophic guilds: primary<br>producers (plants), fungi and bacteria, collembola, mites, other belowground arthropod primary consumers, belowground arthropod secondary consumers, pathogenic protists, bacterivorous protists, secondary consumer protists, Lepidoptera (primary consumers), other aboveground arthropod primary consumers, aboveground arthropod secondary consumers, birds (tertiary consumers) and bats (tertiary consumers). Abundance was characterized by measures of cover,<br>species, family number, amplicon sequence variants or operational taxonomic units numbers, and abundance. A sample unit is considered to represent the population of the different trophic groups at our study sites which measured 50 m x 50 m. Data from different years and traps were pooled per plot.
Sampling strategy	In each region, 50 plots (50 m x 50 m) were chosen in secondary wet, mesic and dry grasslands by stratified random sampling from a total of 500 candidate plots on which initial vegetation, soil and land-use surveys were conducted. This ensured that plots covered the whole range of land-use intensities and management types, while minimising confounding factors such as spatial position or soil type.
Data collection	Land use intensity was assessed annually via questionnaires sent to land managers in which they reported the level of fertilisation (kg N ha 1 yr 1), the number of mowing events per year (from one to three cuts), and the number and type of livestock and their duration of grazing (number of livestock units ha 1 yr 1).<br>Mowing and grazing intensities determine the frequency, and intensity, at which aboveground biomass is removed; and thus represent the intensity of disturbance in the plot. Fertilisation provides additional nutrients, and is typically mostly applied in naturally productive plots: it thus represents a resource availability gradient. In our study system, mowing and fertilisation intensities are positively correlated ($r = 0.70$), while grazing and mowing intensities are negatively correlated ($r = 0.61$). Thus, independent effects of each land-use component, and the respective effect of resource availability and disturbance, cannot be reliably estimated. We therefore used a compound index of land-use intensity, characterising a combined resource availability and disturbance gradient. The land-use intensity index (LUI) was calculated as the square-root-transformed sum of standardised measures of global mowing, fertilisation and grazing intensities across the three regions for each year. We calculated the mean LUI for each plot over the years 2008-2018 because this reflects the average LUI around the years when most of the data was collected.<br>In each plot, we measured the relative abundance of multiple guilds using standard methodology. We sampled vascular plants in an area of 4 m x 4 m on each plot, and estimated the percentage cover of each occurring species every year from 2008 to 2019. Aranaea, Coleoptera, Hemiptera and Orthoptera were sampled by conducting 60 double sweeps along three 50-m plot-border transects. Lepidoptera were recorded along three 300-m transects, each during 30 min. Birds were recorded using audio-visual point counts, at the centre of each respective grassland plot (50 m x 50 m). Bats were sampled along two 200-m plot-border transects: acoustic recordings were taken in real time with a Pettersson-D1000x bat detector (Pettersson Electronic AG, Uppsala, Sweden). To sample belowground bacterial, fungal and protist communities, fourteen soil cores (diameter 4.8 cm) were taken from a 20 m x 20 m subarea of each grassland plot, and soil from the upper 10 cm of soil was homogenised after removal of root material. The bulk sample was split into representative subsamples to analyse each group. Oribatid mites and Collembola were sampled using a Kempson extraction from four soil cores of 4.5 cm x 10 cm per plot. Details of each survey can be found in Extended Methods Table 2.<br>Trait data was obtained from multiple sources. Abundance data and trait databases were matched using the GBIF taxonomy (package traitdataform) when necessary. Guild-specific details on trait data acquisition and treatment can be found in Extended Methods Table 3. Briefly, aboveground plant functional traits were extracted from the TRY database, aggregated from Central European measurements. Belowground plant trait data was determined in pot experiments for most plant grassland species, and this represented at least 90% cover of all plots. Bird trait data was assembled from the literature Bat trait data was extracted from published datasets. We used data from Gossner et al. 2016 completed using additional sources, for Aranaea, Coleoptera, Hemiptera and Orthoptera; the traits were recoded to allow ranking of some traits (e.g. dispersal ability) across these four orders. Day-flying moths and butterfly traits were combined from previous data syntheses). Oribatid mites and Collembola traits were collated from the literature and completed with expert assessments for voltinism. Protist traits were extracted from literature reviews, completed with expert assessments for size data. Microbial (fungi and bacteria) traits covered both community-level properties that serve as trait proxies (C:N and fungal:bacteria ratio, based on PFLA (phospholipid fatty acids) ratio in a mixed sample from each plot; proportion of parasitic fungi, identified from Illumina MiSeq ASV abundances, functionally assigned through the FUNGuild (version 1.0;85) and individual bacterial traits from a published database, aggregated at the genus or order level when possible.<br><br>Considering the large number of data collectors involved, they are not listed here, but for all Biodiversity Exploratories datasets then

can be found in the metadata (all datasets linked in the Data availability section).

Timing and spatial scale	The timing of the sampling was selected to coincide with the annual peak of biological activity for each of the different trophic groups and thus varies from group to group.<br>Plants were sampled from mid-May to mid-June, 2008-2018.<br>Lepidoptera were sampled 3 times between May and August, 2008.<br>Aboveground arthropods were sampled annually from 2009 to 2017 in June and August.<br>Birds and bats were sampled annually from March to June, from 2008 to 2012.<br>Protists DNA was extracted from soil cores sampled from July to August in 2011 and 2017.<br>Fungi and bacteria were sampled from soil cores from July to August in 2011, 2014, 2017.<br>Mites and collembola were sampled in 2019.
Data exclusions	For analyses across guilds, we excluded one guild (belowground primary consumer arthropods) as more than 20% of plot data were missing due to complete absence of this functional guild in some plots
Reproducibility	There are no experiments in the study. Our data were collected as part of a monitoring over several years and which cannot be repeated.
Randomization	Study plots were selected from 3000 candidate plots. Surveys of initial vegetation and land use were conducted on candidate plots by stratified random sampling to ensured that the selected plots covered the whole range of land-use intensity and to minimize confounding effects of spatial position or soil type.
Blinding	Investigators were not aware of the land-use intensity of the plot were they worked, but they could not otherwise be blinded during data collection and analyses for example with respect to the year a sample came from.

Did the study involve field work? ☒ Yes ☐ No

Field work, collection and transport

Field conditions	For aboveground arthropods, the sampling was carried out during the day, when the vegetation was dry (no rainfall) and wind speed was low.<br>For birds, the sampling was carried out during the morning chorus (sunrise-11:00h) when the wind speed was low. In exceptional cases, observations were made during the evening chorus (last 3 hours before sunset).<br>For all other organisms, the sampling was operated at all weather conditions.
Location	Our data were collected in three German regions: (1) Schw5bische Alb in south-western Germany (420 km2, 460-860 m above sea level (a.s.l.)); (2) Hainich-Dun in central Germany (1560 km2, 285-550 m a.s.l.); and (3) Schorfheide-Chorin in northeastern Germany (1300 km2, 3-140 m a.s.l.).
Access & import/export	Fieldwork permits were issued were issued from 2008 tb 2021 by the responsible state environmental offices of Baden-Wurttemberg, Thuringen, and Brandenburg.
Disturbance	Activity of investigators was spatially limited to the 50 m x 50 m grassland plots. We used small paths to access the plots and carefully ensure to avoid any damage to the habitat. Destructive sampling was minimized by using protocols described in the Methods section.

Reporting for specific materials, systems and methods

We require information from authors about some types of materials, experimental systems and methods used in many studies. Here, indicate whether each material, system or method listed is relevant to your study. If you are not sure if a list item applies to your research, read the appropriate section before selecting a response.

Materials & experimental systems

n/a	Involved in the study	
☒	☐	Antibodies
☒	☐	Eukaryotic cell lines
☒	☐	Palaeontology and archaeology
☐	☒	Animals and other organisms
☒	☐	Clinical data
☒	☐	Dual use research of concern
☐	☒	Plants

Methods

n/a	Involved in the study	
☒	☐	ChIP-seq
☒	☐	Flow cytometry
☒	☐	MRI-based neuroimaging

Animals and other research organisms

Policy information about studies involving animals; ARRIVE guidelines recommended for reporting animal research, and Sex and Gender in Research

Laboratory animals	No laboratory animals
Wild animals	In order to quantify the diversity of belowground microorganisms, soil samples were collected in the field and then transport to the lab were the DNA or RNA was isolated from soil and sequenced. Arthropods were collected in the field and killed using ethanol. Identification of arthropods requires killing and transport to the lab were microscopes can be used.
Reporting on sex	Sampled and observed animals were not split according to sex; both males and females were recorded.
Field-collected samples	Aboveground arthropod samples were stored in 93% ethanol at 7°C except for short time periods during transport, sorting and identification.<br>Belowground insect herbivores', predators' and decomposers' samples were stored in plastic cups and transported as soon as possible to the laboratory in cooled boxes (10°C), extracted in 70% diethylene glycole and stored in 70% ethanol. Soil samples for DNA extraction were transported in cooled boxes (4-8°C) to the field where within few hours after soil excavation the soil was sieved and roots were removed. These samples were then frozen at 20°C. Soil samples for RNA extraction were flash-frozen directly after excavation either in liquid nitrogen or dry ice. Soil samples were shipped from the field labs to the institute laboratories on dry ice. Until nucleid acid extraction, soil samples were stored either at -20°C (for DNA extraction) or liquid nitrogen (for RNA extraction). The DNA and RNA nucleic acid extracts were stored at -20°C or -80°C, respectively.
Ethics oversight	It could not be ruled out that threatened or protected arthropod species would be collected and killed. Thus, permission was required from the authorities which was granted for scientific reasons. The responsible state environmental offices of Baden-Württemberg (Regierungspräsidium Tübingen), Thüringen (Thüringer Landesverwaltungsamt) and Brandenburg (Landesumweltamt Brandenburg) approved the study protocol.

Note that full information on the approval of the study protocol must also be provided in the manuscript.

Dual use research of concern

Policy information about dual use research of concern

Hazards

Could the accidental, deliberate or reckless misuse of agents or technologies generated in the work, or the application of information presented in the manuscript, pose a threat to:

No	Yes	
☒	☐	Public health
☒	☐	National security
☒	☐	Crops and/or livestock
☒	☐	Ecosystems
☒	☐	Any other significant area

Experiments of concern

Does the work involve any of these experiments of concern:

No	Yes	
☒	☐	Demonstrate how to render a vaccine ineffective
☒	☐	Confer resistance to therapeutically useful antibiotics or antiviral agents
☒	☐	Enhance the virulence of a pathogen or render a nonpathogen virulent
☒	☐	Increase transmissibility of a pathogen
☒	☐	Alter the host range of a pathogen
☒	☐	Enable evasion of diagnostic/detection modalities
☒	☐	Enable the weaponization of a biological agent or toxin
☒	☐	Any other potentially harmful combination of experiments and agents

Plants

Seed stocks	Not applicable (no seed used)
Novel plant genotypes	Not applicable
Authentication	Not applicable
